# Medicinal plants administered to control hypertension in Ethiopia: ethnomedicine, pharmacology, nutraceutical, phytochemistry, toxicology, and policy perspectives

**DOI:** 10.3389/fcvm.2025.1514911

**Published:** 2025-09-05

**Authors:** Dejen Nureye, Getnet Tadege, Silesh Dubale, Dereje Kebebe, Sultan Suleman, Elvine Pami Nguelefack-Mbuyo

**Affiliations:** ^1^ Research Unit of Neuro-Inflammatory and Cardiovascular Pharmacology, Faculty of Science, University of Dschang, Dschang, Cameroon; ^2^ Department of Pharmacy, College of Health Sciences, Debre Markos University, Debre Markos, Amhara, Ethiopia; ^3^ Department of Pharmacy, Mettu University, Mettu, Oromia, Ethiopia; ^4^ School of Pharmacy, Institute of Health, Jimma University, Jimma, Oromia, Ethiopia; ^5^ Jimma University Laboratory of Drug Quality (JuLaDQ), School of Pharmacy, Institute of Health, Jimma University, Jimma, Oromia, Ethiopia

**Keywords:** hypertension, medicinal plants, pharmacological, anti-hypertensive, nutraceuticals, compounds, policy, Ethiopia

## Abstract

In traditional folk medicine, medicinal plants are widely employed. High blood pressure, a major cause of morbidity and mortality in healthcare settings, is linked to the risk of cardiovascular illnesses and many other serious health issues that can develop from it. This review provides background regarding hypertension, including introductory concepts, risk factors, and treatment approaches. Hypertension may not be effectively treated with the use of diuretics, ACE inhibitors, beta-blockers, alpha-blockers, calcium channel blockers, direct vasodilators, renin inhibitors, etc. These drugs’ side effects include intolerance, weakened disease control, and improper therapy management. Therefore, an approach for extracting new therapeutic chemicals from medicinal plants is receiving attention today. As a result, this article provides a list of 85 plant species from 40 families, compiling data on ethnobotanical claims, plant parts used to make extracts, different types of extracts and study animals, nutracuticals’ intended use, the antihypertensive effect of the extracts, their mode of action, clinical trials, toxicity profile, etc. It also mentions 55 specific chemical compounds that have shown potential to lower blood pressure in lab tests and live subjects, along with their dosage and how they work, based on online searches of published studies from different sources. Researchers looking into and developing new anti-hypertensive therapies to treat hypertension would benefit from our current work. We also tried to address the policy implications.

## 1 Introduction

Cardiovascular diseases (CVDs) are a major health problem worldwide, affecting both developed and developing nations. They're the leading cause of illness and death (1). For example, in Taiwan, CVDs are the second-most common cause of death (2). The term CVD refers to disorders of the heart and blood vessels. The most common types include hypertension (HTN), atherosclerosis (AS), coronary heart disease (CHD), cerebrovascular disease (CBVD), peripheral vascular disease (PVD), heart failure (HF), cardiac arrhythmia (CA), thrombosis, and dyslipidemia (DL) (1).

HTN is the primary cause of cardiovascular (CV) and kidney diseases, but diabetes mellitus (DM), smoking, and DL are also significant risk factors (3). DL involves abnormal levels of cholesterol (high total cholesterol (TC) and low-density lipoprotein cholesterol (LDLC) but low high-density lipoprotein cholesterol (HDLC)) and abnormalities in different lipoprotein particles, which can increase the risk of HTN and heart attacks (HAs). Conditions like nephrotic syndrome (NS), hypothyroidism, obesity, and DM can lead to DL (1).

Metabolic syndrome (MS), characterized by a cluster of conditions like impaired glucose tolerance (GT), DL, obesity, and HTN, is a major risk factor for CVDs. Central obesity, which is particularly dangerous, can lead to insulin resistance (IR), pre-diabetes, type 2 diabetes (T2DM), DL, high blood pressure (BP), AS, increased blood clotting, and inflammation. Hypercholesterolemia (HC) contributes to AS, a condition where cholesterol buildup in artery walls can lead to diseases like CHD, ischemic CBVD, and PVD (4). LDLC is particularly linked to AS and CHD. Both high BP and high cholesterol increase the risk of AS, a complex inflammatory condition that damages artery walls (5).

High BP, also referred to as arterial HTN, is a chronic and progressive condition that affects people across the globe, regardless of their background (6–8). It is characterized by a persistent rise in arterial BP, leading to serious health problems such as CHD, stroke, sudden cardiac death, congestive heart failure (CHF), renal insufficiency (RI), and dissecting aortic aneurysm (9, 10). HTN is typically diagnosed when arterial BP consistently measures at or above 140 mmHg systolic and/or 90 mmHg diastolic on two or more occasions, averaged over two separate visits that include both in-office and out-of-office readings (11, 12). If high BP is recorded three times within a month, HTN is diagnosed (13). Many people with HTN may be unaware of their condition because it often presents no early symptoms, earning it the nickname “the silent killer” (14). This is particularly true for mild-to-moderate primary HTN, which can remain symptomless for years. HTN significantly increases morbidity and mortality rates among adults and imposes a considerable financial burden on healthcare systems (11). It is generally classified into two types: primary/idiopathic/essential HTN, which accounts for 90%–95% of cases, and secondary HTN, which is characterised by elevated systemic BP due to identifiable causes. While essential HTN cannot be cured, it can be managed (4, 12).

HTN is becoming more common worldwide. This is due to factors like population growth, aging populations, and unhealthy lifestyles (9, 14, 15). Around 1.28 billion people between the ages of 30 and 79 have HTN, and two-thirds of them live in low- and middle-income countries (16). HTN contributes to a significant number of deaths worldwide, particularly in low- and middle-income countries (8, 12). The prevalence of HTN varies by region. High-income countries have lower rates of HTN compared to low-income countries (9). In Africa, the prevalence of HTN is high, especially in sub-Saharan Africa (18). Many people in these regions are unaware of their HTN and don't receive treatment (Rx). HTN is a major cause of death in Africa, particularly among adults. The number of deaths related to HTN has increased significantly in recent decades (6, 12). It is prevalent in Ethiopia, though the exact prevalence varies depending on the study (4, 12). Some studies suggest that up to 30% of the population may have HTN (19, 20). HTN has become a major public health concern in Ethiopia, contributing to a significant number of deaths from CVDs and strokes (21). The increasing prevalence of HTN is linked to factors like sedentary lifestyles, smoking and alcohol consumption, employment in manufacturing industries, increased stress, unhealthy diets, and rising life expectancy (12).

Various factors can increase the risk of developing HTN. Since the exact causes of primary HTN are unknown, the term “risk factors” is considered appropriate. These risk factors are classified into modifiable ones (such as a high body mass index (BMI) or obesity, sedentary lifestyle, stress, high salt and/or fat intake, deficiencies in micronutrients like calcium (Ca) and potassium (K), excessive alcohol consumption, and smoking) and non-modifiable ones (including genetic factors, age, sex, and race) (4, 22).

Research in Ethiopia has identified factors such as residence, khat chewing, limited consumption of fruits and vegetables, and physical inactivity as significant contributors to HTN. The lifestyle of the Ethiopian population has evolved in recent years due to urbanization and demographic changes, possibly leading to a rise in HTN prevalence (23). Globally, alcohol consumption is widespread, but its misuse poses public health concerns. Numerous studies have shown that long-term excessive drinking often leads to CV damage and physiological dysfunction, with chronic alcohol use raising BP and contributing to HTN. Excessive salt consumption is also a global issue, linked by several epidemiological studies to elevated BP and an increased risk of HTN, which can eventually lead to heart, liver, and kidney problems. Prolonged alcohol and salt consumption can have detrimental effects on these organs, as well as increase oxidative stress (OS) and DL associated with AS, which are risk factors for HTN (9).

Maintaining normal BP is a complex physiological process that relies on the coordinated function of several control systems, including the cardiovascular, renal, neurological, endocrine, and local tissue systems. It's widely recognized that these hemodynamics are closely linked to BP regulation. Arterial BP is positively correlated with cardiac output (CO) and peripheral vascular resistance (PVR). CO is directly influenced by heart rate (HR) and stroke volume (SV) (24). HR is determined by the rate of action potentials generated by the heart's primary pacemaker cells, with the sympathetic nervous system (SNS) increasing HR and the parasympathetic nervous system (PNS) decreasing it. Key factors that regulate left ventricular SV include preload (filling pressure or venous return (VR) or end-diastolic volume (EDV)), afterload [pressure caused by arteriolar resistance (AR)], HR, and myocardial contractility (MC). PVR is mainly determined by the diameter of arterioles, which in turn is controlled by the constriction of vascular smooth muscle cells (VSMCs) around them. Vasodilator and vasoconstrictor factors simultaneously influence VSMCs within any given tissue. As a result, normal BP is maintained through the interplay of these interconnected systems (25).

Ethiopia has a long history of using herbal therapy; numerous traditional cultures employ medicinal herbs to treat a variety of diseases, including HTN. Examining these plants’ traditional applications, methods of preparation, and cultural significance is necessary to comprehend their ethnopharmacology. The preservation of traditional Rx approaches and their integration into contemporary healthcare systems both greatly benefit from this information. Understanding the pathophysiology of HTN facilitates the identification of specific factors that influence disease development. It also clarifies the approaches by which traditional medicine handles HTN. Ethiopian traditional healers are well-versed in the uses and benefits of medicinal plants. By tracing their practices and Rxs for HTN, we can learn more about these culturally and traditionally based alternative healthcare philosophies. This viewpoint is essential for bridging the gap between traditional healing methods and Western medicine.

Scientific support for the safety and effectiveness of medicinal plants (MPs) used to treat HTN is provided by their pharmacological effects. Showing the active ingredients causing their therapeutic effects may result in the development of novel medicines or dietary supplements for the Rx of HTN. In addition to their therapeutic uses, several of the MPs used in Ethiopia are also sources of nutraceuticals, which are bioactive substances with beneficial health benefits. Exploring these plants’ nutritional and functional properties highlights the significance of diet in the prevention and Rx of chronic diseases including HTN.

Information on the bioactive components and modes of action of MPs can be gleaned from their chemical makeup. This view is crucial for discovering possible drug interactions or negative effects, standardizing herbal formulations, and guaranteeing their quality and consistency. Encouraging their sustainable use, promoting their therapeutic potential, and incorporating them into conventional healthcare practices are all dependent upon a comprehensive review of the MPs used in Ethiopia to control HTN from a variety of angles, including ethnopharmacology, ethnomedicine, pharmacology, nutraceuticals, and phytochemistry. This is necessary to address the increasing prevalence of HTN.

## 2 Current options in treating hypertension

Rx and management approaches for HTN differ greatly between countries. Natural resources were utilized in Africa for therapeutic reasons (6). Two well-established practices for lowering BP are lifestyle changes and prescription medications. Device-based management is growing in acceptance, but it hasn't been proven to be a trustworthy Rx alternative (24, 26, 27). Unless there is a special indication, monotherapies (single-drug Rx) are often advised as the first line of Rx for HTN. The use of a second medicine, if necessary, or switching to a different drug are the next steps after monotherapy in an effort to determine the best therapy for each patient. However, in the USA, the amount of anti-hypertensive medication to be given to drug-naive individuals depends on how much their BP is elevated. Two-drug Rx, either as a single tablet combination or two separate drugs, should be started in individuals whose BP is more than 20/10 mmHg over the goal of therapy (28). With the argument that BP is a multi-regulated variable dependent on several compensatory pathways, combination Rx (acting in multiple mechanisms) usage is now encouraged in the European Union for the majority of patients (26). This action helps to speed up the early management of BP and helps to maintain proper long-term BP control.

Different drugs are used to treat HTN. Diuretics are essential drugs for HTN-Rx. While loop diuretics (high-ceiling diuretics) such as furosemide inhibit the Na^+^/K^+^/Cl^−^ co-transporter in the ascending loop of Henle, potassium-sparing diuretics (acting in the collecting tubule) such as spironolactone correct K^+^ loss during the Rx with thiazide and loop diuretics. Na^+^ and Cl^−^ reabsorption is hindered in the kidney's distal tubules by thiazide diuretics like hydrochlorothiazide. The class of drugs known as diuretics also includes osmotic diuretics and carbonic anhydrase inhibitors (CAIs) (4, 24, 29, 30).

Another class of anti-HTN drugs is angiotensin-coverting enzyme inhibitors (ACEIs), which include enalapril and captopril. In addition to inhibiting Ang-II production, ACEIs also dilate blood vessels and lower BP by inhibiting the kininase 2 enzymes and reducing bradykinin (BK) degradation. Angiotensin receptor blockers (ARBs) like losartan and valsartan have a mostly similar action to ACEI; nevertheless, ACEIs only partially prevent the production of ang-II. ARBs can inhibit the actions of Ang-II on the AT_1_-receptor, whether it is produced by ACE or another enzyme such as cardiac chymase (4, 24, 31).

Calcium channel blockers (CCBs) are divided into two groups: dihydropyridines (such as nifedipine and amlodipine), which produce excellent BP control by directly relaxing the smooth muscles (SMs) surrounding arteries, and non-dihydropyridines (such as verapamil and diltiazem), which lower BP by simultaneously inducing vasodilation and reducing MC. CCBs prevent the passage of extracellular Ca^2+^ through ion-specific channels. The L-type channels in humans are inhibited by the CCBs that are now on the market, despite the fact that several varieties of these channels have been discovered. CCBs cause natriuresis by raising glomerular filtration pressure, dilating afferent arterioles, and increasing renal blood flow in the kidney (4, 24).

Beta-blockers (BBs) are among the drugs that prevent the effects of endogenous catecholamine on β-adrenergic receptors. By inhibiting β-receptors in the brainstem and in peripheral tissues, including the heart, BBs improve HTN and decrease SNS activity. These drugs work well on young people and white people. BBs are divided into first, second, and third generations because of differences in their selectivity for β_1_/β_2_-adrenergic receptors and vasodilatory effects. Miscellaneous agents comprise alpha-1 blockers (prazosin, terazosin, and doxazosin) that block α_1_-adrenergic receptors, thereby inhibiting vasoconstriction induced by catecholamines; centrally acting α_2_-agonists (methyldopa and clonidine) that stimulate α_2_-receptors at the vasomotor center; and direct vasodilators (such as hydralazine and minoxidil). Hydralazine (used to treat hypertensive crises) inhibits inositol-1,4,5-triphosphate (IP_3_)-induced release of Ca^2+^, whereas the minoxidil mechanism is via modulating K^+^ _ATP_‒channels in the VSMCs, allowing K^+^ efflux and SM relaxation. Additionally, as their vascular effects appear to be connected to the formation of nitric oxide (NO) gas as a consequence of their metabolic breakdown, nitrates and sodium nitroprusside may be considered as NO donors. The 2007 approval of the novel medicine aliskiren functions as a direct renin inhibitor (4, 24).

Modern drugs significantly decrease CVD-related mortality. However, most of the population, particularly in third-world nations, cannot afford the long-term use of these drugs. Additionally, some patients still have a long way to go before reaching their goal BP and decreasing CV problems. One of the major contributing factors to poor response is thought to be medication-related issues or polypharmacy, especially in older individuals and those with concomitant illnesses. Poor adherence and Rx failure may result from this (7, 24). Drugs made from synthetic materials are pricey and can lead to the appearance of new diseases, among other adverse outcomes. Additionally, there is a decrease in patient compliance with taking more than a single tablet daily. With this in mind, HTN patients, particularly those living in rural regions, look for alternate methods, such as herbal Rxs, to treat their HTN and other conditions (1, 3).

## 3 Significance of medicinal plants in managing hypertension

Herbal medicines are gaining increasing importance in the Rx of HTN due to their broad therapeutic benefits, high safety profile, natural compatibility with the human body, cultural acceptance, wide availability, and lower cost. For example, natural diuretics from plants are expected not to cause K-depletion since many plants contain K along with other minerals such as Na, Mg, Ca, and zinc (Zn) (14, 15, 32). Beneficial plant-derived cations like K, Mg, and Ca contribute to anti-HTN effects by promoting endothelial-dependent vasorelaxation through various mechanisms, including CCB, reducing Na^+^ reabsorption, inhibiting Ang-II secretion, and stabilizing vascular cell membranes (33).

With the growing population, trends towards holistic health, and rising cost of living and chronic diseases, there is an increasing demand for herbal medicines. Researchers are turning to natural resources for the Rx of HTN and other conditions (34). Natural ingredients have played a crucial role in the development of CV drugs. Ethiopia, with its rich ethnobotanical knowledge and vast biodiversity, offers a unique opportunity to explore the medicinal potential of plant-based compounds. The country is home to around 6,500–7,000 species of higher plants, 12% of which are endemic. Ethiopia is recognised as one of six countries worldwide where 60% of the plants are believed to be native and possess medicinal properties. Ethiopian MPs are reported to have significant hypotensive effects (7, 10, 24, 35).

MPs represent the earliest form of healthcare known to humanity and have been instrumental in the development of modern civilization. According to the World Health Organization (WHO), 80% of the global population continues to rely on traditional or herbal remedies for their basic healthcare needs. Today's pharmacopoeia includes 7,000 medicinal substances, which account for about 25% of all medications. The WHO also notes that approximately 34% of plant-derived drugs directly align with their traditional uses in indigenous cultures (36, 37). Of the 252 medicines deemed essential by the WHO, 11% are exclusively plant-based, while many others are synthetic drugs derived from natural sources. Over 2,000 plants have been identified with therapeutic properties for treating HTN, offering cardioprotective, cardioactive, cardiotonic, or circulatory-stimulating effects (38). For example, reserpine, an alkaloid from the root extract of *Rauwolfia serpentina*, is highly effective as a first-line Rx for lowering systolic blood pressure (SBP). The anti-HTN effects of MPs are attributed to their tannins, galloyl derivatives, flavonols, flavones, phenylpropanoids, proanthocyanidins, and flavonoid glucosides (39). In several African countries, particularly Ethiopia, up to 80% of the rural population relies on MPs for Rx. MPs have long played a crucial role in both healthcare and diet in global societies (10).

Natural antioxidants found in plants are generally responsible for preventing or slowing the harmful effects of oxidative stress (OS), which is considered the root cause of aging and various human diseases such as AS, stroke, HTN, DM, cancer, and neurological disorders (37). In certain HTN models, antioxidants not only help reduce elevated BP but also mitigate inflammation, fibrosis, sclerosis, and dysfunction of the kidneys, heart, and other organs. Numerous plant-derived enzymes and non-enzymatic secondary metabolites can scavenge reactive oxygen species (ROS), thereby protecting the body from oxidative damage (10). Polyphenols and flavonoids have been shown to possess free radical-scavenging and renoprotective effects. They can be used as adjunctive Rx in hypertensive patients with impaired renal function (40, 41).

Polyphenols have vasorelaxing properties and promote NO production, leading to a reduction in BP (40). Flavonoids can enhance endothelial function (EF) and prevent platelet aggregation, thereby reducing the risk of CVD (42). Tannins, which are polyphenolic compounds, also possess vasorelaxant effects, similar to acetylcholine (Ach), with most of their effects being endothelium-dependent. Additional mechanisms for the vasorelaxant effects of polyphenols include the inhibition of protein kinase C (PKC), cyclic nucleotide phosphodiesterases (PDEs), and/or reduced Ca^2+^ uptake (43). Terpenoids derived from various plants have been shown to lower BP primarily by inhibiting L-type calcium channels (CCs) (44). Certain plant extracts have been found to inhibit angiotensin-converting enzyme (ACE). The bioactive components (phenolic acids, alkaloids, polyphenols, flavonoids, tannins, polysaccharides, and sterols) of these medicinal plants have demonstrated ACEI activity (38). Similarly, soybean saponins have been shown to lower BP by inhibiting renin and the renin-angiotensin-aldosterone system (RAAS) pathway (43). Flavonoids have been shown to reduce the production of aldosterone by the kidneys and antidiuretic hormone (ADH) by the pituitary gland (42). Alkaloids exhibit effects similar to BB drugs (42).

Research indicates that certain components in plants, such as saponins, K, and phenolic compounds, can promote diuresis, saluresis, and natriuresis either individually or in combination by (a) disrupting the reabsorption of water and electrolytes (Na^+^ and Cl^−^) in the renal tubules or (b) inhibiting Na^+^/K^+^ -ATPase activity in the kidneys by directly binding to the enzyme, impairing its function, or altering membrane fluidity. Some theories also suggest that these compounds may influence the interaction between membrane phospholipids and Na^+^/K^+^ -ATPase pumps (45, 46). Flavonoids promote vasodilation in the afferent arterioles of the renal vasculature, which increases the glomerular filtration rate (GFR), leading to greater water excretion and lower BP (46). Another mechanism by which flavonoids exert their diuretic effects involves interaction with adenosine A_1_-receptors. Plant alkaloids are also recognized for their diuretic properties, particularly those containing benzyl isoquinoline-type alkaloids (47). Saponins contribute to diuresis by promoting the excretion of Na^+^ and other electrolytes along with water, which decreases plasma volume and, in turn, CO (42). Plant extracts and their metabolites can also lower HTN through their anti-inflammatory effects (48).

Unlike DNA sequences, epigenetic modifications are potentially reversible, making them attractive targets for modern or personalized medicine. By maintaining the balance between histone acetyltransferases and histone deacetylases/lysine deacetylases and preventing hypermethylation, CVD-causing factors such as OS, cell proliferation, and inflammation can be mitigated. Notably, research is advancing on the use of plants and plant-derived compounds to influence histone structure. Although there are no reports from Ethiopia, Chinese traditional MPs have shown potential interactions with human enzymes that modify histones. Some of these medicinal compounds may promote histone condensation, a process with significant implications for the pathology of various diseases, including CVDs. Hesperidin from citrus fruits and lycopene from tomatoes are known to inhibit methylation. Catechins from tea, curcumin from turmeric, and coumaric acid from cinnamon act as inhibitors of acetylation and methylation. Additionally, allyl sulfides found in garlic can inhibit histone deacetylases. Recent findings suggest that a polyherbal blend may regulate class I and II histone deacetylases. Tanshinone 1, a compound from *Salvia miltiorrhiza*, has been shown to inhibit histone H3 acetylation. Rosmarinic acid also epigenetically influences peroxisome proliferator-activated receptors (PPAR), which play a key role in CV physiology, including BP regulation. Recent research indicates that herbs and their components may play a crucial role in diseases and health benefits related to microRNA (miRNA). For example, grape extract has been shown to downregulate miRNAs associated with inflammation, providing a positive immunomodulatory effect in individuals with HTN (49).

Nutraceuticals have been gaining significant attention (50), particularly for their potential role in managing HTN through the regulation of OS. Antioxidants have been shown to mitigate HTN-related changes caused by OS, leading to numerous studies focused on improving this chronic condition through increased intake of antioxidant-rich foods (51). Fruits, vegetables, and other plant-based foods high in phenolics, especially flavonoids, have been identified as offering substantial health benefits due to their oxidative damage reduction properties. These benefits are attributed to their ability to bind metal ions, scavenge free radicals, activate endogenous antioxidant enzymes, and inhibit oxidative chain reactions (52). In individuals with normal or stage-1 HTN, a diet rich in vegetables and fruits ensures sufficient intake of Mg and K, which has been shown to significantly lower diastolic blood pressure (DBP) by 3.1/2.1 mmHg (53). A diet high in K and an increase in serum K^+^ levels, even within the physiological range, promote endothelium-dependent vasodilation by hyperpolarization (HP) via activation of Na^+^ -pumps and opening of K^+^ -channels. The HP is transmitted from the endothelium to VSMCs, leading to a reduction in cytosolic Ca^2+^ and resulting in vasodilation. High K intake may also reduce stroke risk by inhibiting VSMC proliferation, free radical production, and arterial thrombosis. Experimental evidence suggests that K can decrease macrophage adhesion to the vascular wall, which is critical in the development of arterial lesions, endothelial OS, and the production of vascular eicosanoids (54).

Peptides, phenolic compounds, and flavonoid-rich foods (edible plant materials) have been shown to lower BP and inhibit ACE (38). Among the bioactive peptides, ACEI peptides are particularly notable. To be effective, they must be stable in the gastrointestinal tract and capable of being delivered to the cardiovascular system (CVS) (31). Consuming phytochemicals—particularly a diet rich in methyl donors—can help regulate DNA hypomethylation levels and reduce CVD risk factors. Folate plays a key role in DNA synthesis and regulation as a methyl donor. In fact, a diet low in folate during pregnancy can negatively impact the health of the unborn child, leading to an obese phenotype and increasing the risk of HTN in adulthood. Conversely, a diet high in methyl donors during pregnancy can protect against obesity in offspring (49).

This review seeks to connect the pathogenesis of HTN and the pharmacology of antihypertensive drugs with the ethnomedicinal practices, potential mechanisms of action, and clinical relevance of Ethiopian MPs used in HTN Rx. It provides ethnobotanical information on the MPs utilized in Ethiopia for managing HTN. The review also presents *in vitro*, *in vivo*, and *ex vivo* experimental evidence supporting the pharmacological effectiveness of these MPs in reducing HTN in various animal models. Along with preclinical research, it discusses clinical studies and anti-HTN compounds isolated from these ethnobotanically recognized plants. Additionally, the paper highlights the unexplored areas and opportunities for developing a strong foundation for current alternatives and future research aimed at discovering new plant-based anti-HTN drugs.

## 4 Sources of data and search methodologies

Ethnobotanical information about the claimed MPs found in Ethiopia and their extracts, metabolites, and active compounds, as well as preclinical and clinical efficacy studies done anywhere, was searched and downloaded from global databases such as PubMed, Web of Science, Scopus, SciELO, ResearchGate, Google, Google Scholar, ScienceDirect, and AJOL. For all ethnomedicinal, pharmacological, and phytochemistry information, original articles and published papers, including conference proceedings written in English, were compiled and examined based on their categories, and the data were summarized in tables and figures. The papers are considered without restriction of publication or online access time. Absence of a full scientific name is the exclusion criteria for ethnobotanical studies. Duplicated papers are excluded in the ethnobotanical and experimental studies. Unpublished data (data from MSc theses and PhD dissertations) is also excluded. Review articles are considered in the discussion part. The keywords utilised during retrieval of ethnobotanical sources include “ethnobotanical survey”, “medical plants”, “medicinal herbs”, “ethnobotanical study” + “Ethiopia”, “ethnobotanical study” + “hypertension”, “medicinal plants” + “blood pressure”, “traditional medicine”, “ethnomedicine” + “Ethiopia”, “ethnobotany” + “medicinal plants” + “hypertension”, “indigenous knowledge” + “hypertension” + “Ethiopia”, “folk medicine” + “blood pressure” + “Ethiopia. Reports made on the medical use of plants for HTN, or high BP, were presented in terms of local name, family name, additives used, parts used, preparation methods, etc. Taking the ethnobotanical information into account, a combination of keywords such as “scientific name of the plants” + “*in vivo*”, “*in vitro*”, “antihypertensive activity”, “antihypertensive effect”, “blood pressure”, “hypertension”, “hypotensive activity”, “hypotensive effect”, “ethnopharmacological”, “pharmacological studies”, “vasorelaxant effect”, “vasodilator”, “diuretic activity”, “diuretic effect”, preclinical study, “ACE inhibitory activity”, “clinical study”, “clinical trial”, “patients”, “hypertensive patients”, “common name of the plant+antihypertensive activity”, bioactive compounds, metabolites, etc. were used to search and collect relevant data to present findings of preclinical and clinical studies as well as experiments on phytochemistry of those plants in terms of extract/Rx type, isolated compounds, study model, route of administration, doses administered, possible mechanism of action/s, study design, patient types, duration of Rx, results, etc.

## 5 Results and discussion

### 5.1 Ethnomedicine of medical plants used to control raised blood pressure in Ethiopia

#### 5.1.1 Diversity of medical plants

This review identified 85 MP species from 70 genera across various regions of Ethiopia that are traditionally used to treat HTN (Supplementary Material 1). This number is higher than reports from Iran (22 species), the Tenggerese society (41 species), and Ghana (39 species) (111–113). The higher number of documented species may reflect the strong reliance of Ethiopians on traditional medicine (TM), likely due to the high cost of modern drugs, limited availability and accessibility of modern healthcare services, and the cultural acceptance of herbal remedies (114). Conversely, the number is lower than reports from Guinea (97 species), Morocco (104 species), and South Africa (117 species) (115–117), which could suggest either a lack of extensive research in Ethiopia or differences in research methodologies. As shown in Table 1, the most commonly reported plant species used to manage HTN in Ethiopia are *Thymus schimperi* (11.7%), *Rumex abyssinicus* (9.0%), *Moringa stenopetala* (8.1%), *Lupinus albus* (4.5%), *Ajuga integrifolia* (3.6%), and *Calpurnea aurea* (3.6%). The frequent citation of specific plant species or families may indicate a higher potential for bioactive compounds, making them priority candidates for future pharmacological study (118).

**Table 1:** Frequently reported medicinal plant species used to control hypertension in Ethiopia.

Plant species	Citation no.	Percentage	Plant species	Citation no.	Percentage
*Thymus schimperi*	13	11.7	*Catha edulis*	2	1.8
*Rumex abyssinicus*	10	9.0	*Carica papaya*	2	1.8
*Moringa stenopetala*	9	8.1	*Citrus aurantium*	2	1.8
*Lupinus albus*	5	4.5	*Citrus aurantiifolia*	2	1.8
*Ajuga integrifolia*	4	3.6	*Citrus medica*	2	1.8
*Calpurnea aurea*	4	3.6	*Hibiscus sabdariffa*	2	1.8
*Citrus limon*	3	2.7	*Meriandra dianthera*	2	1.8
*Foeniculum vulgare*	3	2.7	*Otostegia integrifolia*	2	1.8
*Allium sativum*	3	2.7	*Passiflora edulis*	2	1.8
*Artemisia absinthium*	3	2.7	*Persea americana*	2	1.8
*Bersama abyssinica*	3	2.7	*Rosa abyssinica*	2	1.8
*Dovyalis abyssinica*	3	2.7	*Rosmarinus officonalis*	2	1.8
*Hagenia abyssinica*	3	2.7	*Ruta chalapensis*	2	1.8
*Mentha×piperita*	3	2.7	*Satureja punctata*	2	1.8
*Ocimum lamiifolium*	3	2.7	*Thymus serrulatus*	2	1.8
*Rumex nepalensis*	3	2.7	*Trigonella foenum-graecum*	2	1.8
*Allium cepa*	2	1.8	*Zingiber officinale*	2	1.8

**Figure 1:**
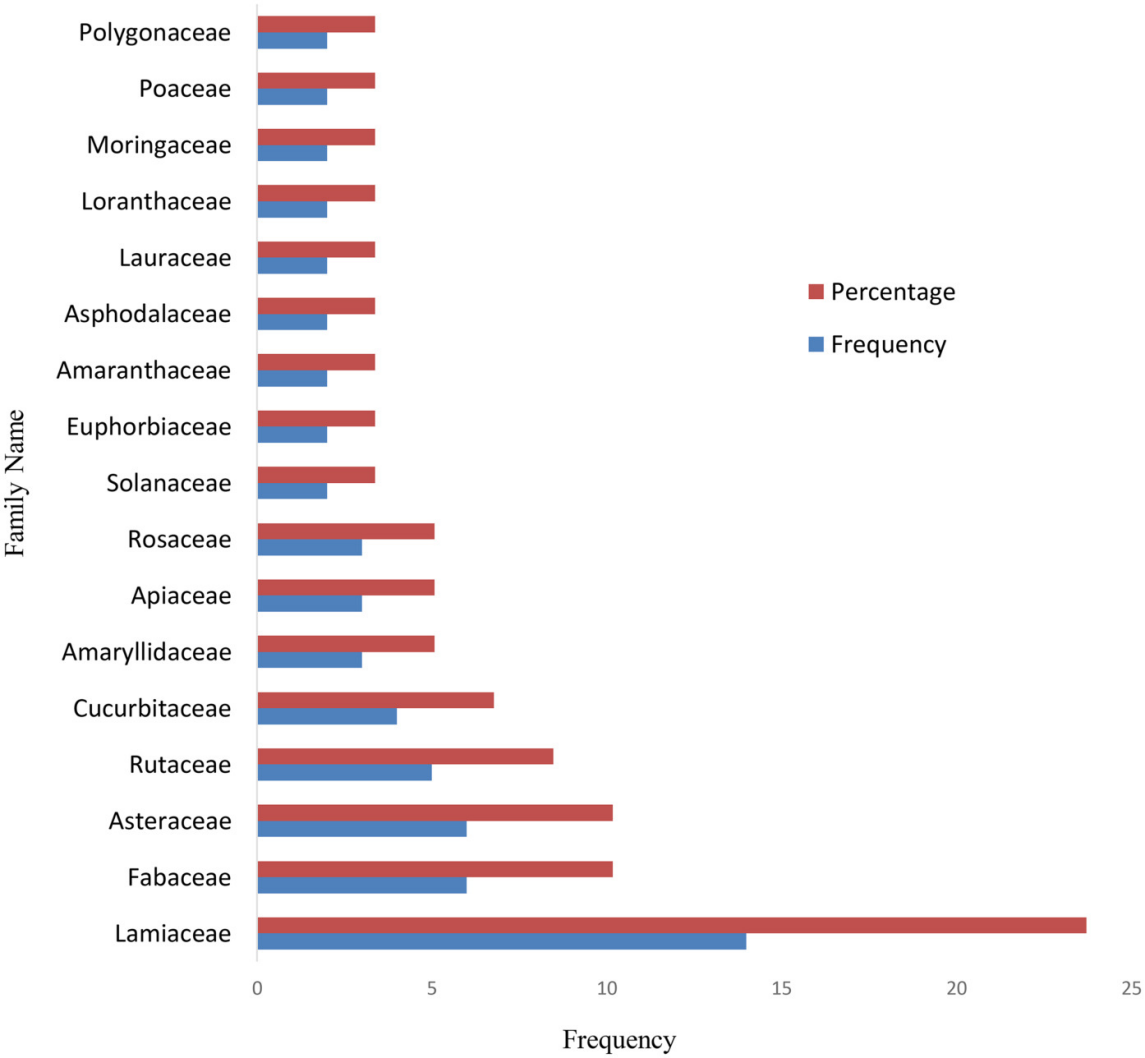
Frequently used medical plant families to control hypertension in Ethiopia.

The documented MPs were categorized into 40 families, with the most prevalent being Lamiaceae (23.72%), Fabaceae (10.17%), Asteraceae (10.17%), Rutaceae (8.47%), and Cucurbitaceae (6.78%). Amaryllidaceae, Apiaceae, and Rosaceae each contributed 3 species (5.08%) (Figure 1). These findings are consistent with studies from Morocco, where most HTN-treating plants belong to the Lamiaceae (18 species), Asteraceae (10 species), Apiaceae (8 species), Fabaceae (4 species), and Solanaceae (3 species) families (116). Similarly, in Ghana, many of the plants used for HTN Rx are from the Fabaceae, Cucurbitaceae, and Lamiaceae families (113). In South Africa and Guinea, Asteraceae and Fabaceae are the most commonly represented families (115, 117). In Ethiopia, around 23 families are represented by a single species effective against HTN. The Fabaceae, Asteraceae, and Solanaceae families are particularly prominent in Ethiopian flora (119). The dominance of the Lamiaceae (120), Asteraceae (121, 122), Fabaceae (120, 121), and Rutaceae (118, 123) families has also been noted in surveys of MPs used for various ailments in Ethiopia. This data highlights the cultural and medicinal significance of the Lamiaceae, Asteraceae, Fabaceae, and Rutaceae families in managing HTN in Ethiopia (123).

#### 5.1.2 Parts of medical plants used and preparation conditions

**Figure 2:**
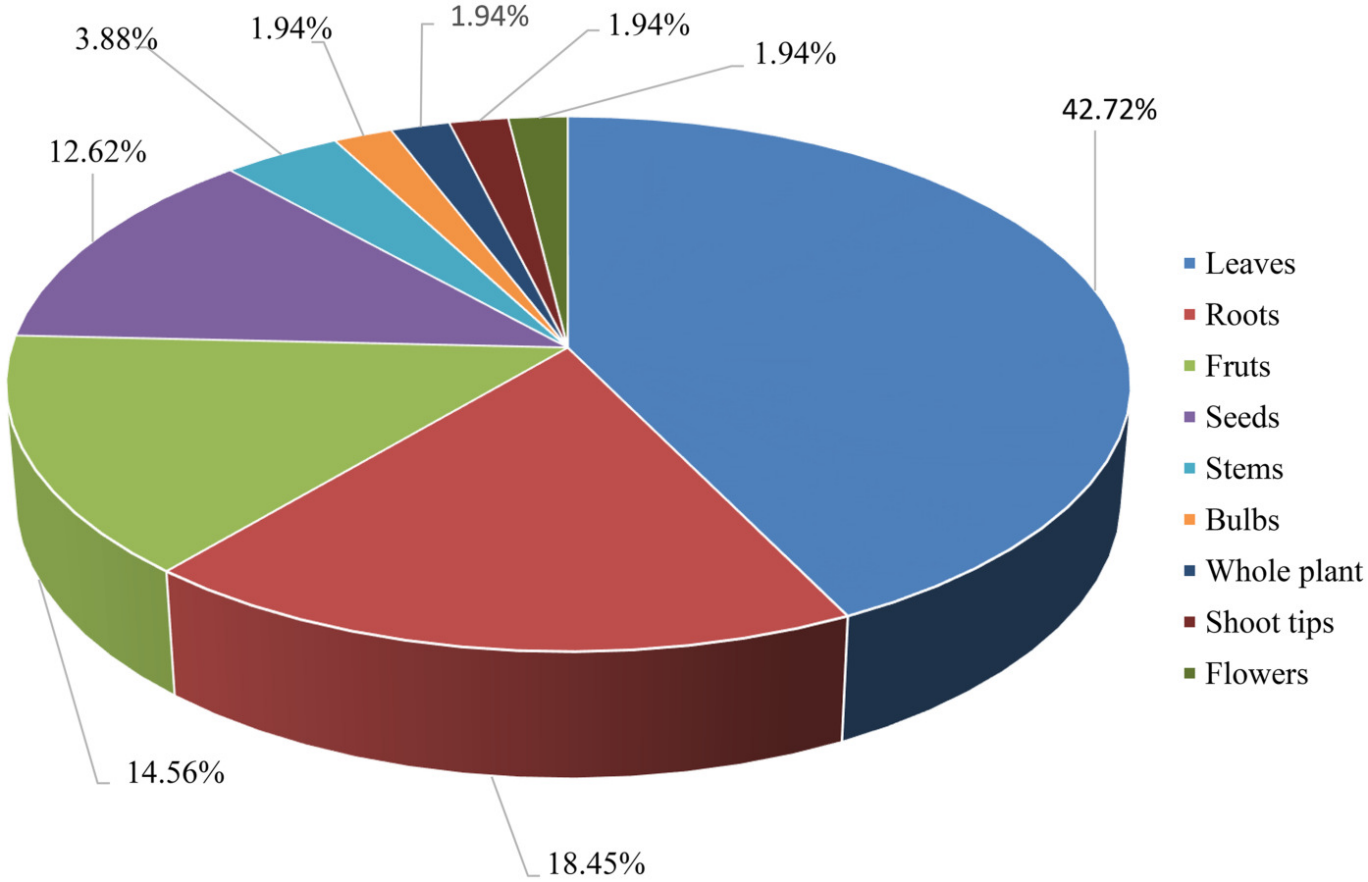
Frequently used parts of medical plants to manage hypertension in Ethiopia.

The use of plant parts and their preparation methods are influenced by their availability and the knowledge of indigenous people (122). While various plant parts are used for remedy preparation, leaves were the most commonly used (42.72%), followed by roots (18.45%), fruits (14.56%), seeds (12.62%), and stems (3.88%), as shown in Figure 2. These findings align with other studies that identified leaves as the most frequently used part for treating HTN (113, 115, 116, 124). This pattern is similar to traditional wound Rx practices in Ethiopia, where leaves are the most commonly used, followed by roots and fruits. Other ethnomedicinal studies in Ethiopia have similarly reported that leaves are the most frequently utilized plant parts, likely due to their greater availability, ease of preparation, and the effectiveness of their phytoconstituents (125).

Research indicates that TM practitioners in various African countries primarily use plant leaves. Leaves produce the majority of plant secondary metabolites, making them a rich source of chemically active compounds that are relatively easy to extract (118). Promoting the use of leaves for remedy preparation is recommended as a more sustainable approach to accessing plant materials, as harvesting leaves allows the parent plant to continue its life functions, unlike root harvesting, which typically kills the plant (119). While plant roots are also rich in potent bioactive compounds, their frequent use in herbal remedies can endanger the survival of the plant species. To ensure the sustainable use of MP resources, it is important to adopt proper harvesting techniques and conservation strategies (118).

In some instances, multiple parts of the same plant were used either separately or in combination. Remedies were prepared using plant parts in dry, fresh, or both forms. Fresh plant parts, which are rich in bioactive metabolites, are often preferred for formulating remedies in Ethiopia, as they can be quickly and conveniently prepared into medicines using methods such as crushing, squeezing, maceration, infusion, and decoction (119). Most of these anti-hypertensive botanicals are used as monotherapies, although some involve combinations of multiple MPs. Ethiopian herbal remedies for treating HTN that involve mixtures of two or more different MPs include *Allium sativum* with *Allium porrum*; *Carica papaya* with *Ajuga integrifolia*; *Embelia schimperi* with *Ruta chalepensis* and *Rumex abyssinicus*; *Mentha spicata* with *Carissa spinarum* and *Citrus aurantifolia*; *Moringa stenopetala* with *Allium cepa* and *Capsicum annuum*; and *Rumex abyssinicus* with *Allium sativum* (Supplementary material 1).

#### 5.1.3 Techniques of community recipe preparations and additives used

**Figure 3:**
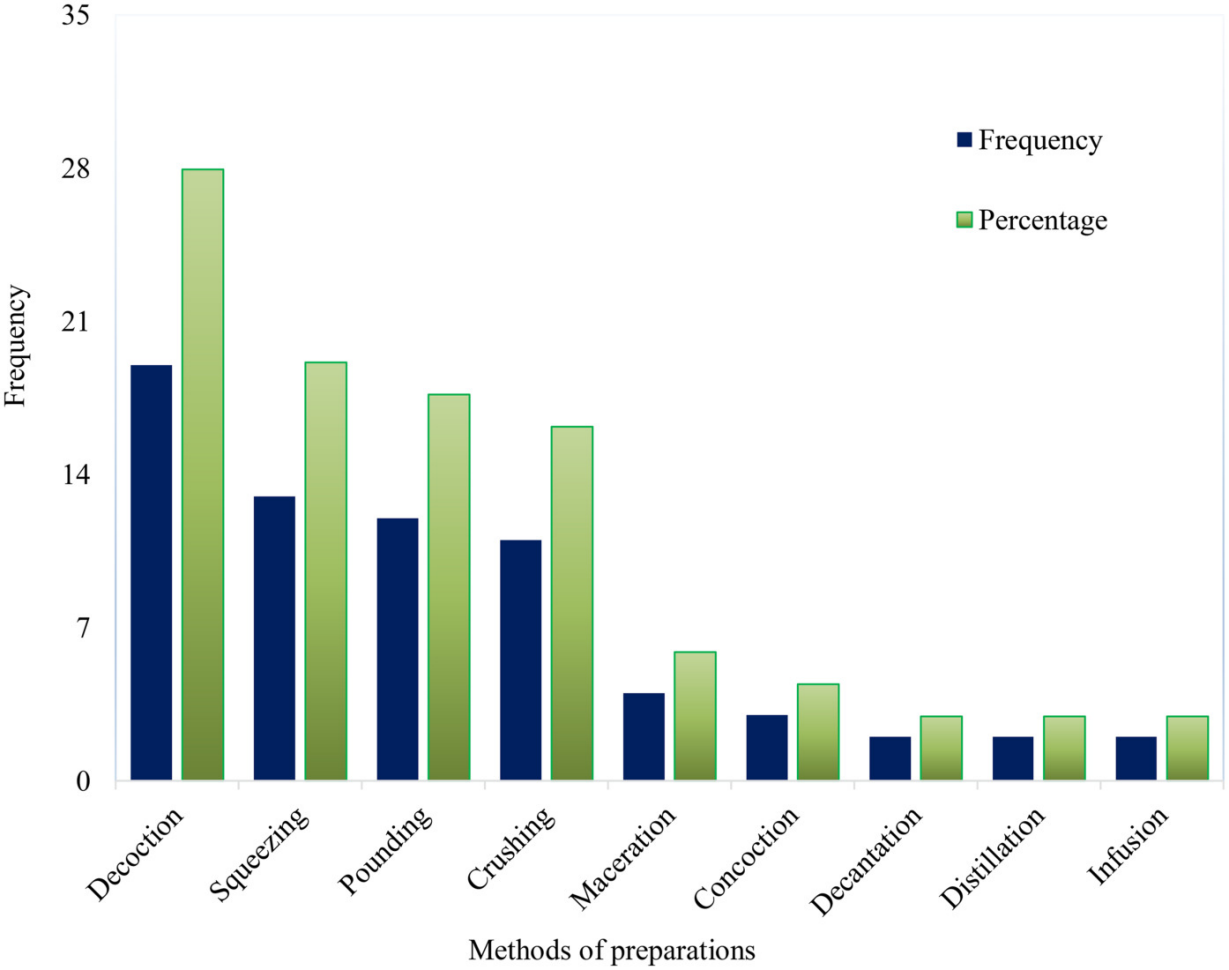
Common preparation forms of traditional medicines for hypertension in Ethiopia.

Traditional healers follow various techniques and strict procedures when preparing herbal therapies, even though they rely on simple methods and tools. They do not require advanced processing methods or equipment, likely due to the absence of processing instruments and formal education (120). Healers use either a single method or a combination of techniques for preparation. The most common methods for preparing anti-hypertensive herbal remedies include decoction (27.94%), squeezing (19.12%), crushing (17.65%), and pounding (16.18%), as shown in Figure 3. These methods are also commonly used in the preparation of anti-malarial remedies in Ethiopia (121). The preference for chewing and crushing may be linked to the ease of preparation and the availability of local tools, such as stones. Similarly, decoction has been highlighted as the primary preparation technique in studies conducted in Ghana (113), Guinea (115), Morocco (116), and Iran (124).

**Figure 4:**
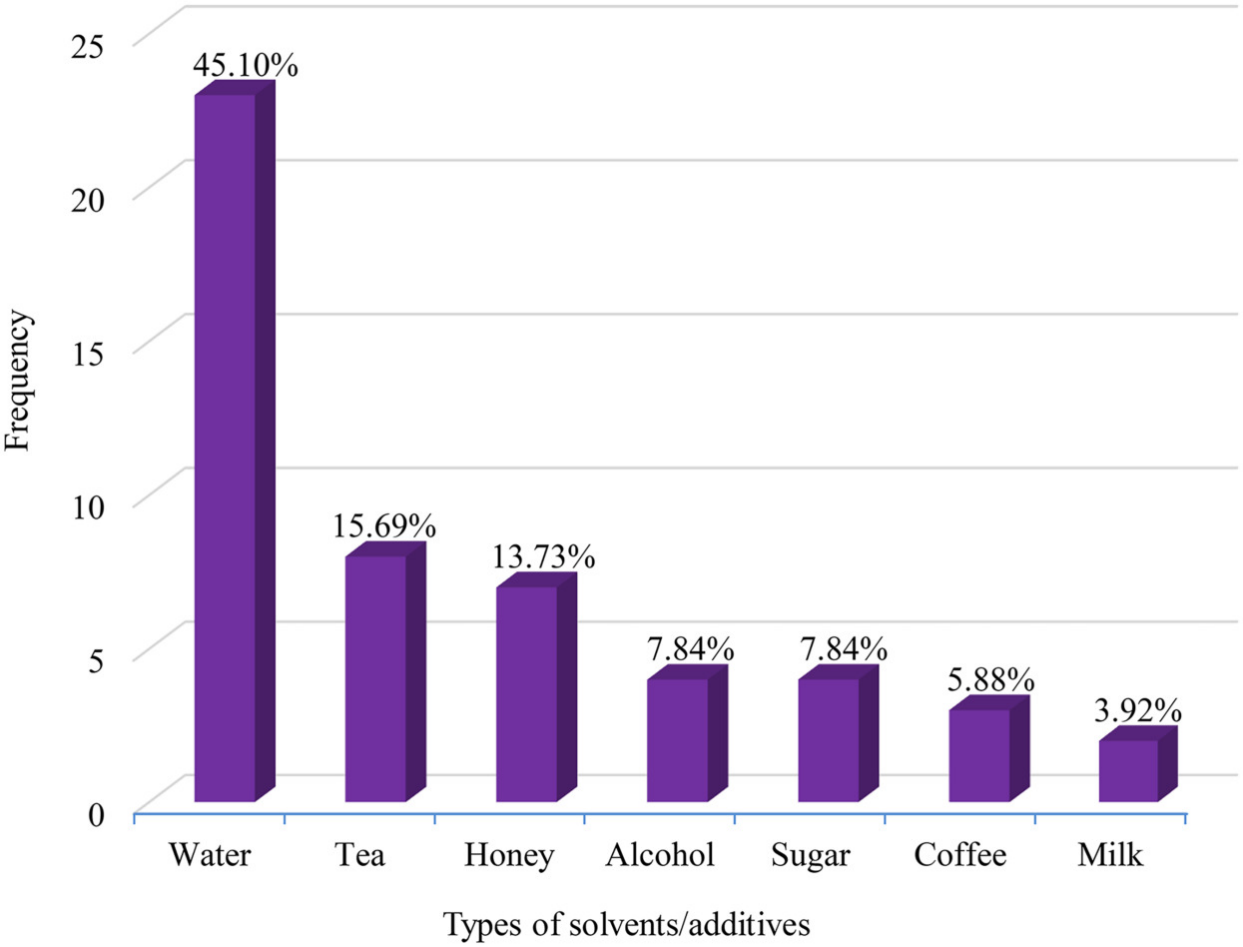
Commonly employed solvents/additives in antihypertensive medical plants remedy in Ethiopia.

MPs were prepared in various forms using different additives and solvents. Among the solvents used, water was the most common (45.10%), followed by tea (15.69%). Other solvents like alcohol (7.84%), coffee (5.88%), and milk (3.92%) were used less frequently. Various additives such as honey (13.73%) and sugar (7.84%) were incorporated into the preparations (Figure 4). Water and tea are popular solvents because many metabolites dissolve easily in them, and high temperatures help to quickly extract active components. Additives and solvents were mainly used to enhance the effectiveness of the remedy and create favorable healing conditions by either reducing toxicity or improving the flavor of the Rx. This could be due to the synergistic effects of combinations that contain multiple pharmacologically active substances, increasing the likelihood of interactions with a wide range of biological targets. These interactions can influence the remedy's availability, absorption, distribution, bioactivity, enzyme activity, and selectivity (118).

#### 5.1.4 Route, frequency, duration, and doses of administration

As shown in Table 2, the most common method of administering anti-hypertensive herbal remedies was oral intake, with drinking (65.52%), eating/chewing (24.14%), and, less frequently, intranasal administration (10.34%). This preference for oral administration may be due to the simplicity of this method, the challenges of using other parenteral routes, and concerns about potential side effects in the event of an overdose. This finding aligns with numerous studies on other diseases in Ethiopia (120). Similarly, in other African countries, oral administration is also the preferred method for managing HTN (113, 115). HTN is a chronic and systemic condition, requiring precise delivery of therapeutic compounds at the correct blood concentration. The oral route is non-invasive and convenient, allowing for relatively rapid absorption and distribution of the active ingredients in herbal remedies, thus providing adequate therapeutic effects (98). Typically, herbal medicines are recommended to be taken once, twice, or three times daily for periods ranging from three consecutive days to six months. Some traditional healers advise patients to take the remedy in the morning, at bedtime, or on an empty stomach before meals. Dosages are often expressed as a glass, a cup, or half a cup. It is well known that traditional healthcare systems often face challenges due to a lack of precision and standardization (118).

**Table 2:** Common forms of antihypertensive herbal remedy application in ethipia.

Application form	Frequency	Percentage
Drinking	38	65.52
Eating/chewing	14	24.14
Inhaling/sniffing intranasal	6	10.34

Supplementary material 2 provides a detailed review of other local and traditional uses of MPs used for treating HTN in Ethiopia. Overall, many of the ethnobotanical studies were localized, lacking comprehensive nationwide surveys. In most of the survey, the frequency of administration and the dosage used were not reported. In some of the reported studies, methods of preparation and application are not mentioned. In some other published papers that are not included here, the list of plants with their corresponding indications, method of preparations, and other descriptions are not available in table format. This reduces the total number of species identified and reported.

### 5.2 Pharmacological and nutraceutical evidences of the reported medicinal plants

**Figure 5:**
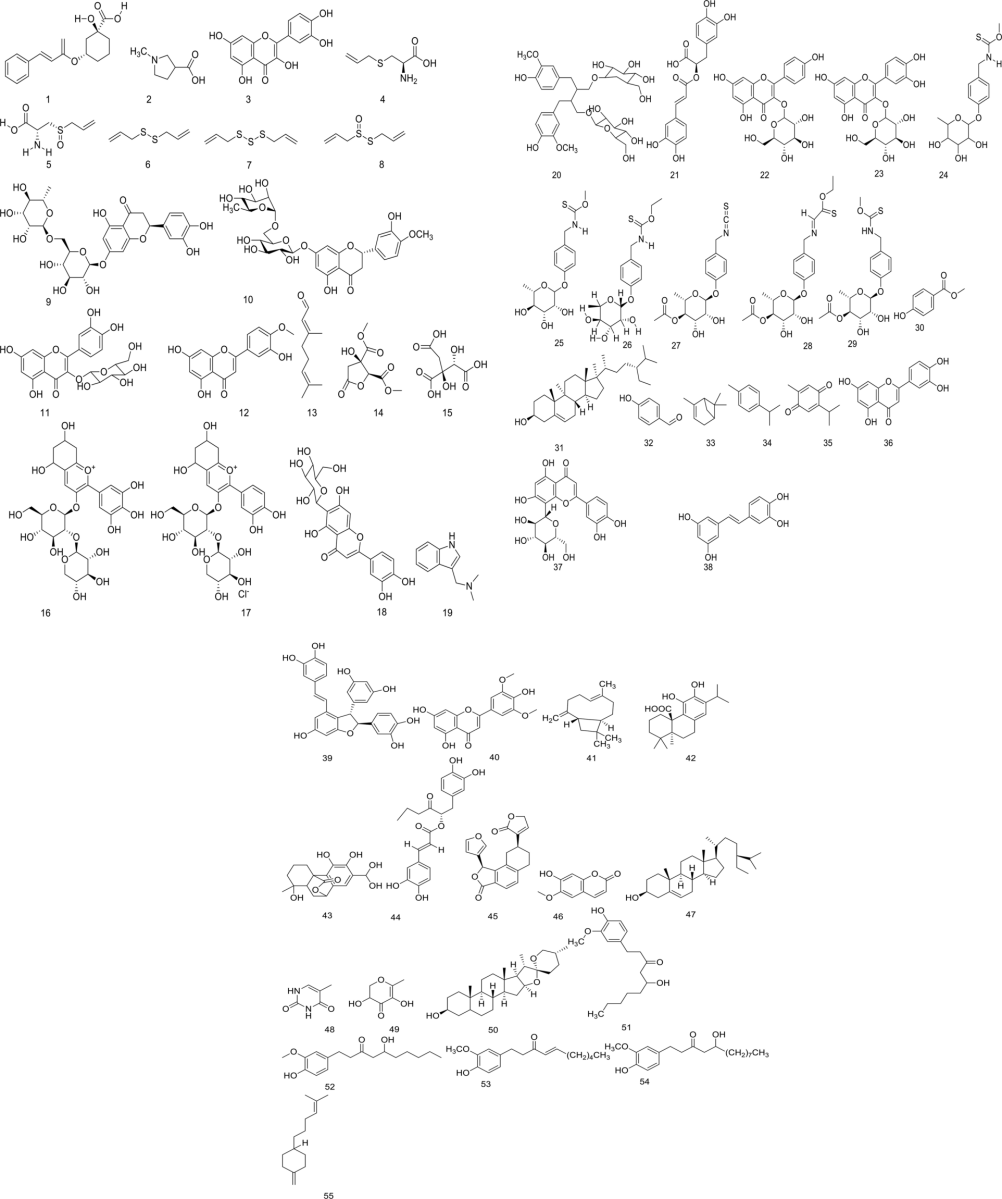
Chemical structure of bioactive compounds isolated from the studied antihypertensive medicinal plants.

The findings of preclinical studies are summarized in Tables 3–6, while the clinical investigation results are presented in Table 7. Out of 85 Ethiopian MPs traditionally claimed to have anti-HTN properties, about 21 showed positive anti-HTN effects in *in vivo*, *in vitro*, or *ex vivo* studies. Around 11 plants demonstrated anti-HTN benefits both in preclinical models and clinical studies. The secondary metabolites (Table 8) and compounds (Table 9 and Figure 5) isolated from these plants also exhibited significant anti-HTN activity in preclinical studies. Extracts, plant materials, and isolated active compounds from around eight plants were reported to have substantial anti-HTN effects in preclinical models. Approximately five plants had robust scientific evidence of anti-HTN effects in human studies and animal, tissue, and *in vitro* assays. One plant showed *ex vivo* bioactivity, demonstrated solely by its active compound. In total, 46 Ethiopian MPs have confirmed anti-HTN activity through various mechanisms and approaches. The remaining 39 plants are yet to be explored by researchers.

**Table 3:** Preclinical studies on antihypertensive activities of medicinal plants claimed for hypertension treatment in Ethiopia.

S. no.	Medicinal plants	Extract/treatment type(s)	Study animal/tissue/assay	ROA	Study model(s)	Dose(s) and result(s)	Possible MOA(s)	Ref. no.
1	*Acanthospermum hispidum*	Ethanol soluble fraction of aerial parts	Normotensive Wistar rats	PO	*In vivo*, *In vitro*	▪ 30 mg/kg significantly reduced SBP & MAP by 35 & 30 mmHg, respectively	- NO/cGMP pathway activation - Hypotensive effect	(150)
		Ethanol-soluble fraction of aqueous extract	2K1C-ovariectomized rats, & isolated rat mesenteric vascular bed	PO	*In vivo*, *Ex vivo*	▪ 30 mg/kg reduced SBP (144–90), DBP (103–71), MAP (110–77), & HR (251–165) ▪ 300 mg/kg caused a significant urinary excretion of Na^+^ & Cl^−^ ions ▪ 0.001, 0.003, & 0.01 mg significantly reduced vascular perfusion pressure	- Reduced OS, saluretic, & anti-nitrosant action - NO & PG-dependent vasodilator effect	(153)
2	*Allium cepa*	Ethylacetate tunic extract	Normotensive albino rats	PO	*In vivo*	▪ 40 mg/kg significantly decreased SBP, DBP, MAP, & HR by 33, 16, 23 & 7%, respectively	- Hypotensive effect - Negative chronotropy	(174)
		5% dried onion diet	L-NAME-induced & SHRSP HTN in SDR rats	PO	*In vivo*, *In vitro*	▪ Produced significant anti-HTN effects ▪ Decreased TBARS in plasma ▪ Increased the nitrate/nitrite excreted in urine	- Increased NO by increasing NOS activity - Protect NO	(178)
		Aqueous onion bulbs extract	FIH Wistar rats, anti-inflammatory assay	PO	*In vivo, In vitro*	▪ 400 mg/kg reduced SBP from 132.6–122.5 mmHg, & attenuated VCAM-1 expression & increased eNOS effect	- Anti-inflammatory activity	(177)
		70% ethanol peel extract	FIH in Wistar rats, thoracic aortic rings	PO	*In vivo*, *Ex vivo*	▪ 200, 400, & 800 mg/kg attenuated high BP significantly ▪ 0.0625–2 mg/ml reduced aorta contraction in a dose-dependent manner	- Reduced OS - Ca^2+^ influx inhibition	(179)
3	*Allium sativum*	Garlic dialysate	Anaesthetized dogs, isolated rat atria	IV	*In vivo, Ex vivo*	▪ 67.2 mg/kg elicited significant decreases in DBP & HR by 38 & 17%, respectively	- Beta-adrenoceptor blocking action	(183)
		Bulb powder packed in capsules	Anaesthetized dogs	PO	*In vivo*	▪ 2.5−15 mg/kg decreased arterial BP ▪ 15 & 20 mg/kg provoked bradycardia	- Hypotensive activity	(185)
		Aqueous extract of fresh garlic	2K1C SDR rats	IP	*In vivo*	▪ 500 mg/kg reduced BP by 47%	- NO pathway	(186)
		Garlic in diet	Wistar rats	PO	*In vivo*	▪ 2% diet block induced HTN	- Enhance NO production	(187)
		Aqueous garlic cloves extract	Diabetic & 2K1C SDR	IP	*In vivo*	▪ 500 mg/kg caused 66% reduction in SBP		(51)
		Aqueous garlic bulbsextract	FIH Wistar rats, antiinflammatory assay	PO	*In vivo, In vitro*	▪ 150 mg/kg reduced SBP from 132.6–120.7 mmHg, & attenuated VCAM-1 expression	- Increased eNOS activity - Anti-inflammatory activity	(177)
		Aqueous garlic cloves extract	Normotensive & 2K1C Wistar rats	IV	*In vivo*	▪ 20 mg/kg reduced SBP, DBP, MAP, & HR by 16.7, 26.7, 23.1 & 38.4% in normotensive rats & by 22.2, 30.6, 28.2, & 45.2% in hypertensive rats, respectively	- Unkown	(188)
		Processed garlic contain 75.3 mg/100 g *S*-allyl-ʟ-cysteine	SHR rats	PO	*In vivo*	▪ 30 & 50 mg/kg significantly decreased SBP & DBP	- Not studied	(192)
		650 mg bulb powder tablets, & garlic & vitamin-C/& E combination tablets	Human umbilical vein endothelial cells	NA	*In vitro*	▪ 10-μmol/L of various garlic ingredient & garlic ingredients combined with vitamin-C/& E caused vasorelaxant effect 2-fold & 3-fold above control, respectively	- Enhance endothelial NO production	(200)
		Water & 5% ethanol whole fresh garlic extracts	Isolated rat pulmonary arteries	NA	*Ex vivo*	▪ 1–500 μg/ml of the extracts resulted in a dose-dependent vasodilation reaching a maximum of 91 & 93%, respectively	- NO formation activation	(201)
		Fresh garlic cloves juice dissolved in water	Isolated rat aorta rings, human RBCs	NA	*Ex vivo*, *In vitro*	▪ 50, 200, & 500 μg/ml concentrations of garlic caused a dose-dependent vasorelaxation	- H_2_S production	(206)
4	*Calpurnia aurea*	80% methanolic seed extract	Normotensive & 2K1C SDR rats, isolated aortic rings of guinea pig	IV	*In vivo, Ex vivo*	▪ 45 mg/kg reduced SBP, DBP, MAP by 37.8, 45, & 42.2% in normotensive, & by 33.2, 36.1, & 35% in hypertensive rats, respectively ▪ 250 mg/L caused maximum (92.1%) vasorelaxation	- Ca^2+^ influx inhibition	(211)
5	*Carica papaya*	Crude ethanol extract of unripened fruit	Normotensive, renal & DOCA-salt induced HTN albino wistar rats, isolated rabbit aorta, renal artery & vertebral artery	IV	*In vivo, Ex vivo*	▪ 20 mg/kg showed significant MAP depression in both group but caused 28% more depression than the standard drug in hypertensive rats ▪ 10 μg/mL produced significant vasorelaxation attenuated by phentolamine	- Modulating alpha-adrenoceptor activity	(215)
		Ethanolic root bark extract	Hypertensive Wistar rats (renal artery occluded)	IV	*In vivo*	▪ 100 mg/kg produced MAP of 40.88 mmHg (equipotent as that of the standard drug)	- Action on rennin – angiotensin system	(217)
		Nori preparation of leaves	FIH Wistar rats	PO	*In vivo*	▪ 20% diet reduced SBP, DBP, & pulse wave velocity by 37%, 14%, & 33%, respectively	- Reduced arterial stiffness	(222)
		Standardized methanolic leaf extract	WKY & SHR rats	IV	*In vivo*	▪ 100 mg/kg reduced MABP in SHR rats similar to the standard drug	- Baroreflex sensitivity improvement	(223)
6	*Citrus aurantifolia*	Citrus leaf extract supplementation	HPOIH in SDR rats	PO	*In vivo*	▪ Reduced BP		(237)
		Aqueous bark extract	Normotensive rabbits, isolated rat heart & aorta	IV	*In vivo, Ex vivo*	▪ 32 mg/kg dose decrease MAP by 54.31% ▪ 10^−3^ mg/ml of extract decreased inotropic effect by 48% & chronotropic effect by 28%	- Cardiodepression - Endothelium-dependent vasorelaxation	(245)
		70% methanolic fruit juice extract	Anesthetized SDR rats, isolated pork coronary artery	IV	*In vivo, Ex vivo*	▪ 0.75 g/kg produced a significant fall of SBP, DBP, MAP, & HR in both normotensive & glucose-induced hypertensive rats ▪ 0.0001–0.3 mg/ml showed a concentration-dependent vasorelaxation	- Increasing cGMP & cAMP via inhibition of vascular PDEs enzymes	(246)
7	*Citrus limon*	5% diluted lemon juice	SHR rats	PO	*In vivo*	▪ Suppressed an increase in BP		(249)
		5% lemon juice squeezed residue	SHR rats	PO	*In vivo*	▪ Significantly lowered SBP & DBP		(250)
8	*Citrus medica*	Aqueous *C. medica* fruits, *C. citratus* stems & fresh leaf, *P. americana* fresh leaf, & honey mixture extract	Sucrose-induced HTN in Wistar albino rats	PO	*In vivo, In vitro*	▪ MAP & HR lowered respectively by 16.86 & 4.94% at 50 mg/kg, by 25.81 & 7.95% at 100 mg/kg, & by 28.95 & 10.56% at 150 mg/kg	- Improvement in oxidative status - Protect vascular endothelium damage	(256)
9	*Coriandrum sativum*	70% methanolic fruit extract	Normotensive SDR rats, isolated guinea-pig atria & rabbit aorta	NA	*In vivo, Ex vivo*	▪ 30 mg/kg produced a fall in BP with 40.84% maximum effect ▪ 5 mg/ml caused high inhibitory effect on atrial & aortic force of contractions	- CCB activity - Cholinergic effect	(267)
		Ethyl acetate & hot water extracts of aerial part	Rat isolated aorta	NA	*Ex vivo*	▪ 1 mg/ml ethyl acetate extract showed biphasic endothelium-based vasorelaxation ▪ 3 mg/kg water extract showed only slow vasorelaxation independent of endothelium	- NO releasing - Blocking the influx of Ca^2+^ via Ca^2+^**-**channels	(268)
10	*Cymbopogon citratus*	Aqueous aerial part extracts	Salt & ethanol-induced HTN in Wistar rats	PO	*In vivo*	▪ 200 mg/kg significantly decreased SBP, DBP, MAP, & HR	- Reduced OS	(9)
		Leaves infusion	Distal segements of human internal thoracic arteries	NA	*Ex vivo*	▪ 0.0002 mg/ml significantly inhibited adrenergic-mediated vasoconstriction ▪ 0.002–0.2 mg/ml caused vasorelaxant effect with 6.46% maximal relaxation	- COX-mediated vasorelaxation	(284)
11	*Hibiscus sabdariffa*	Cold & hot aqueous calyces extracts	L-NAME induced HTN in Wistar rats	PO	*In vivo*	▪ 250 mg/kg of cold & hot extract significantly reduced SBP from 172.6 mmHg to 141 & 125.2 mmHg, respectively	- Elevated eNOS - Rise plasma NO levels	(294)
		Crude methanolic calyces extract	Isolated aortic rings of SHR rats	NA	*Ex vivo*	▪ 1 mg/ml produced maximum vasorelaxation effect (27.9 & 86.01% reduction in K^+^ & PE-contractions, respectively)	- Endothelium-derived NO-cGMP-pathway - Ca^2+^ influx inhibition	(297)
		80% methanolic leaf extract	Salt-induced HTN in Wistar rats & biochemical assay	PO	*In vivo*	▪ 400 mg/kg significantly reduced SBP, DBP, MAP & HR	- Reduction in serum OS	(298)
		Crude methanolic calyces extract	Isolated thoracic & abdominal SDR rat aorta	NA	* Ex vivo*	▪ 1 mg/ml relaxed PE pre-contraction by 76% ▪ 2 mg/ml relaxed KCl pre-contraction by 68%	- Inhibition of Ca^2+^ influx via VGCCs	(310)
		Aqueous calyces extract	SDR rats, isolated kidney	PO	*In vivo, In situ*	▪ 1,000 & 2,500 mg/kg produced diuretic, natriuretic, & potassium sparing effects	- Modulation of aldosterone activity	(301, 314)
12	*Linum usitatissimum*	Flax lignan concentrate from seeds	2K1C Wistar rats	PO	*In vivo*	▪ Reduced BP in a dose-dependent manner ▪ 800 mg/kg reduced SBP, DBP & MAP similar to that of captopril	- Renal ang-II reduction - Plasma ET-1 inhibition - NO & NOS induction	(352)
			DOCA-salt induced renal HTN in wistar rats	PO	*In vivo*	▪ 800 mg/kg significantly decreased SBP (152.5–129.3), DBP (107.1–90.65), & MAP (129.8–110 mmHg)	- Reduced OS - Antagonism of RAAS	(353)
		70% methanolic seed extract	Anesthetized SDR rats, isolated guinea-pig atria & rat aorta	IV	*In vivo, Ex vivo*	▪ 100 mg/kg decreased MAP by 47% ▪ 10 mg/ml inhibited atrial force of contraction (EC_50_ of 3.7 mg/mL), & relaxed the vessels (EC_50_ of 5.7 mg/mL)	- α_1_-receptor blocker - CCB-like activity	(355)
13	*Lupinus albus*	“Gebto Arekei” residue	Hypertensive guinea-pigs	IV	*In vivo*	▪ 200 mg/kg reduced SBP, DBP, MAP by 43.11, 49.75, & 47.02%, respectively	- Unknown	(359)
		Gebto Arekei (distillated seed extract)	Isolated guinea-pig aorta	NA	*Ex-vivo*	▪ 4.74 μg/ml caused 90% vasorelaxation in EPI pre-contracted aortic strips ▪ 21 μg/ml caused 90% vasodilation in KCl pre-contracted aortic strips	- K^+^-channels opening via eNOS pathway - VDCCs inhibition	(360)
14	*Melia azedarch*	Ethanolic leaf extract	DOCA salt-induced HTN in Wistar rats	NC	*In vivo*	▪ 180 & 360 mg/200 g significantly decreased SBP (141–101& 157–104 mmHg) & DBP (112–71 & 108–75 mmHg), respectively	- Unknown	(42)
		Methanolic leaf Extract & its fractions	Normal & Salt-induced HTN in SDR rats, isolated aortic tissue, & right atria	IV	*In vivo*, *Ex vivo*	▪ 300 mg/kg methanol extract reduced MAP by 65*.*36 & 81.44% in normotensive & hypertensive rats, respectively ▪ 300 mg/kg ethyl acetate fraction reduced MAP by 77*.*06 & 88*.*96% in normotensive & hypertensive rats, respectively	- NO & CCB dependent vasorelaxation activity - Negative inotropic & chronotropic effects	(370)
15	*Moringa oleifera*	Ethanolic pod extract & its ethyl acetate phase, & ethanolic seed extract	Anaesthesized normotensive Wistar rats	IV	*In vivo*	▪ 30 mg/kg of the extracts reduced MAP by 41.04, 79.63, & 43.90%, respectively	- Hypotensive activity	(394)
		Water leaf extracts	SHR rats	PO	*In vivo*	▪ 3 ml/kg caused a significant decrease in SBP	- Unknown	(395)
		70% ethanolic leaf extract	L-NAME-induced HTN in Wistar rats	PO	*In vivo*	▪ 500 mg/kg decreased BP from 159.60–102.40 mmHg	- Vasodilator action	(11)
		95% ethanolic leaf extract	EPI-induced HTN in SDR rats	IP	*In vivo*	▪ 462.5 mg/kg decreased BP from 162–118 mmHg	- CCB activity	(396)
		Aqueous leaf extract	L-NAME-induced HTN in Wistar rats, isolated mesenteric artery	PO	*In vivo, Ex-vivo*	▪ 60 mg/kg decreased SBP (193.6–149), DBP (137.5–90.4), MAP (160.3–109 mmHg), & HR (435–339 beat/min) ▪ 0.3 mg produced significant vasorelaxation	- Endothelium-dependent vasodilation & reduced OS	(397)
			Rats, human pulmonary artery ECs, isolated mesenteric arterial beds	IV	*In vivo, Ex-vivo*	▪ 30 mg/kg produced 8.57% reduction in MAP ▪ 30 μg/mL induced significant endothelial NO production	- Activation of eNOS-NO-sGC pathway	(398)
		Aqueous extract of traditional dietary supplement	Rabbits	IV	*In vivo*	▪ 5 × 10^−8^−5 × 10^−2^ mg/kg caused a dose-dependent fall in BP ranging between 7.14 & 100%	- Cholinomimetic effect - Adrenergic receptors antagonism	(399)
		Leaves protein hydrolysate & ultrafiltration fractions	SHR rats	PO	*In vivo*	▪ 100 mg/kg of <1 kDa peptide fraction lowers BP by 17 mmHg		(8)
		Methanolic & ethyl acetate leaf extracts	Hypertensive ICR mice	PO	*In vivo*	▪ 0.3 g/kg of both extracts reduced SBP from 102.35–90.97 & 87.71 mmHg, respectively		(400)
		95% ethanolic & aqueous extracts of leaves, twigs, pods, roots, & seeds	SHR rats	PO	*In vivo*	▪ 1,000 mg/kg of all extract significantly reduced SBP & DBP		(402)
16	*Moringa stenopetala*	Aqueous leaf extract	Normotensive guinea pigs, isolated thoracic aorta strips	IV	*In vivo, Ex-vivo*	▪ 40 mg/kg decreased SBP, DBP, & MAP from 81.91–39.26, 53.16–20.79, & 62.74–26.94 mmHg, respectively ▪ 7 mg/ml inhibited vasoconstriction by 95.56%	- Blockade of VSCCs	(408)
		70% ethanolic & aqueous leaf extracts	FIH Wistar rats	PO	*In vivo*	▪ 1,000 mg/kg of ethanol & aqueous extract decreased SBP (163.67–133.5 &163.67–124.67), DBP (114.5–92.5 & 114.5–85.5), & MAP (130.88–106.18 & 130.88–98.57 mmHg), respectively	- Unknown	(18)
		Microencapsulated product of leaf extract	Isolated guinea pig thoracic aortic strips		*Ex-vivo*	▪ 160 mg/ml produced 74.17% vasorelaxation	- Vasodilation activity	(409)
17	*Nigella sativa*	50% ethanol seed extract	Isolated rat aortic rings	NA	*Ex vivo*	▪ 2–14 mg/ml caused a concentration dependent relaxation in aortic rings pre-contracted by PE & KCL with a maximum effect of 62.3% & 60.2%, respectively	- Ca^2+^ influx inhibition - K_ATP_-channels opening - Intra-cellular calcium release inhibition	(421)
		Seed oil	Isolated rat aortic rings	NA	*Ex vivo*	▪ 10-100μg/mL elicited a dose-dependent relaxation of the aorta	- Block VSCC & ROCCs	(422)
		Aqueous & macerated extracts of seeds	Isolated guinea pig heart	NA	*Ex vivo*	▪ 2.0 & 5.0 mg% of macerated extract & 1.0, 2.0 & 5.0 mg% of aqueous extract caused significant reductions in HR & contractility	- CCB like activity - Opening K^+^-channels	(423)
		Aqueous seed extract	Normotensive wistar rats, isolated rat thoracic aorta	IV	*In vivo, Ex vivo*	▪ 200 mg/kg reduced MAP & HR by 35.83 & 10.16%, respectively ▪ 30 mg/kg reduced aortic ring contraction ▪ force by 55.03%	- SNS inhibition - Increasing endothelial NO synthesis	(424)
		Dethymoquinonated volatile oil	Anaesthetized rats	IV	*In vivo*	▪ 2-16 µl/kg decreased both arterial BP & HR	- Suppress SNS outflow at vasomotor centre in the medulla	(425)
		Volatile oil	Guinea-pigs	IV	*In vivo*	▪ 30-120 μg/kg decreased arterial BP & HR in a dose-depended manner	- 5-hydroxytrptaminergic & muscarinic actions	(426)
		Seed oil	L-NAME-iduced HTN in SDR rats,	PO	*In vivo, In vitro*	▪ 2.5 mg/kg significantly reduced SBP & increased plasma NO level	- Vasorelaxation	(428)
		Seeds or biomass extracts	L-NAME-induced hypertensive rats	PO	*In vivo*	▪ 400 mg/kg normalized increment in SBP & DBP, reversed elevated serum LDH level & increased serum NO level up to three fold	- Reduced OS	(429)
		Seed oil	2K1C-induced RVH in Wistar rats	IP	*In vivo*	▪ 0.2 ml/kg reduced BP & improved left ventricular function		(430)
		Dichloromethane seed extract	SHR rats	PO	*In vivo*	▪ 0.6 ml/kg decreased MAP by 22%	- Hypotensive activity	(431)
18	*Otostegia integrifolia*	80% methanolic leaf extract	FIH in SDR rats & its isolated thoracic aorta	PO	*In vivo, Ex vivo*	▪ 500 mg/kg significantly reduced SBP (121.91 to109.33) & DBP (90.91–87.33) ▪ 318.75 μg/ml caused 100% vasorelaxation	- CCB like activity	(44)
19	*Passiflora edulis*	Methanolic rind extract	SHR rats	PO	*In vivo*	▪ 50 mg/ml caused a decline in BP by 28 mmHg	- Not studied	(445)
		Ethanolic fruit peel extract	SHR rats	PO	*In vivo*	▪ 50 mg/kg decreased SBP, DBP, & MAP by 13.8, 10.2 & 13 mmHg, respectively	- Unknown	(446)
		Fruit peel extract	SHR rats	PO	*In vivo*	▪ 50 mg/kg significantly lowered SBP by 12.3 mmHg & serum NO level by 65%	- NO modulation	(447)
		Fruit pulp	SHR wistar rats	PO	*In vivo*	▪ 8 g/kg significantly reduced the SBP		(448)
		70% ethanolic seed extract, & fruit juice & pulp extract	Rat thoracic aorta, anti-inflammatory assay	NA	*Ex vivo, In vitro*	▪ 200 μg/mL of ethanolic seed extract, & fruit juice & pulp extract caused 31 & 86% vasorelaxation, respectively, in endothelium-intact aortic rings pre-contracted with PE	- Vasodialation & anti-inflammatory activities	(449)
		50% ethanolic & aqueous fruit peel extracts	Mesenteric artery rings	NA	*Ex vivo*	▪ 1,000 μg/ml of ethanolic & aqueous extracts caused vasorelaxant effects in endothelium-intact (45.9 & 31.11%) & endothelium-denuded (77.2 & 9.83%) rings, respectively	- K^+^ - channel opening	(451)
20	*Persea americana*	Methanol & aqueou*s* leaf extracts & fractions from methanol extract	Anaesthetized SDR normotensive rats	IV	*In vivo*	▪ 50 mg/kg methanolic & aqueou*s* extracts caused a decrease in MAP by 36.2 & 35% ▪ 15 mg/kg of PaMCF**_8_** & PaMCF**_11_** fractions caused a fall in MABP by 38.6 & 38.3%, respectively	- Hypotensive activity	(456)
		Aqueous seed extract	SDR normotensive rats	PO	*In vivo*	▪ 260 mg/kg 10-day pre-Rx decreased MAP from 125.7–92.1 mmHg & HR from 274.6–161.6 beats/min	- Not studied	(43)
		Aqueous leaf extract	Wistar rats (normal & with Dahl salts-induced HTN), guinea pig isolated atrial muscle strips, rat isolated portal veins & thoracic aortic rings	IV	*In vivo, Ex vivo*	▪ 25–400 mg/kg significantly declined BP & HR in normotensive & hypertensive rats ▪ 25–800 mg/ml produced negative inotropic & chronotropic effects on guinea pig atria ▪ 50–800 mg/ml reduced the spontaneous, rhythmic, & myogenic contractions of veins ▪ 25–800 mg/ml produced relaxations of endothelium-containing thoracic aortic rings	- α_1_-adrenoceptor blockade - Endothelium-dependent NO production & cGMP release	(460)
		Aqueous leaf extract	Isolated rat aortic rings	NA	*Ex vivo*	▪ 0.01–12.8 mg/ml caused a concentration-dependent vasorelaxation in intact endothelium with an EC_50_ of 0.88 mg/ml	- Ca^2+^ influx inhibition - EDRF synthesis/release - PGI_2_ & PGE_2_ receptors activation	(461)
		Methanol (95%) leaf extract & ethyl acetate fraction	Rat isolated thoracic aortic rings	NA	*Ex vivo*	▪ 2 mg/ml of crude extract & ethyl acetate fraction produced 74.4 & 65% reduction in contraction of endothelium-intact aorta	- α-adrenoceptors antagonisim	(463)
		70% ethanolic leaf & nanoparticle extracts	NaCl-induced wistar rats & NO serum assay	PO	*In vivo, In vitro*	▪ 100 mg/kg ethanolic extract declined SBP & DBP by 48.08 & 35.58 mmHg, respectively ▪ 100 mg/kg nanoparticle extract decreased SBP & DBP by 68.75 & 55.25 mmHg, respectively	- Increase serum NO level	(466)
		Aqueous seed extract	Salt-induced HTN in albino rats	NC	*In vivo*	▪ 700 mg/kg reduced SBP & DBP by 45.2 & 56.14%, respectively	- Not studied	(468)
		Aqueous leaf extract	Normotensive rats & ethanol-sucrose- & EPI-induced HTN in SDR rats	PO	*In vivo*	▪ 50, 100, & 150 mg/kg decreased SBP, DBP & MAP dose-dependently in both normal & hypertensive animals	- Not studied	(469)
21	*Rosmarinus officinalis*	Ten kinds of extracts	Isolated rat thoracic aorta	NA	*Ex vivo*	▪ 0.05-0.80 mg/mL of most extracts showed a dose-dependent relaxant activities	- Increased NO level - Decreased ang-II level	(471)
22	*Ruta chalpensis*	70% ethanolic aerial parts extract	Normotensive guinea pigs & its isolated aorta	IV	*In vivo, Ex vivo*	▪ 80 mg/kg markdly reduced SBP (84.54–58.83) & DBP (60.56–39.50 mmHg) ▪ 10 mg/kg caused 83.47% relaxation of aorta	- Hypotensive activity	(10)
23	*Satureja punctata*	Decoction of the aerial parts	Normotensive & RVH guinea pigs, isolated thoracic aorta	IV	*In vivo, Ex vivo*	▪ 30 mg/kg caused 45.78 & 52.07% fall in MAP in two group of rats, respectively ▪ 40 mg/ml caused 98.19% vasorelaxation effect on aorta precontracted with KCl	- Unknown	(489)
24	*Solanum nigrum*	Aqueous fruit extract	Isolated rat mesenteric arteries	NA	*Ex vivo*	▪ 0.00001–0.02 mg/ml caused a dose-dependent decrease in perfusion pressure in diabetic (143.74–109.23) & non-diabetic rats (83.87–80.98 mmHg) with intact endothelium	- Unknown	(506)
25	*Syzygium guineense*	80% methanolic leaf extract	1K1C-induced HTN in SDR rats, isolated guinea pig aorta	PO	*In vivo, Ex vivo*	▪ 150 mg/kg caused significant reduction in SBP, DBP, & MAP by 26, 17.7, & 21.3% ▪ 70 mg/ml caused maximum (56.22%) vasorelaxation in KCl-precontracted aorta	- Vasodilation activity	(518)
26	*Thymus schimperi*	Aqueous leaf extract	Isolated guinea pigs thoracic aorta	NA	*Ex vivo*	▪ 5 mg/ml caused maximum (99.93%) relaxation in EPI precontracted aorta ▪ Extract also showed relaxation in KCl, Ach, glibenclamide, & histamine pre-contracted aorta in a dose-dpendent manner	- ROCC & VGCC - K^+^_ATP_-channel activation - Activation of H_1_ & M_3_-receptors	(23)
27	*Thymus serrulatus*	Aqueous leaf extract	Isolated guinea pigs thoracic aorta	NA	*Ex vivo*	▪ 0.5–5 mg/mL reduced contraction of aorta in a concentration-dependent manner	- Raises the tissue content of cGMP	(540)
28	*Trigonella foenum-graecum*	Alcoholic seed extract	1K1C SDR rats	PO	*In vivo*	▪ 300 mg/kg reduced SBP significantly from 176.1–160.5 mmHg	- Not studied	(5)
		Methanol seed extract & its methanolic fraction	DOCA-salt induced HTN in Wistar rats, FIH Wistar rats	PO	*In vivo*	▪ 30 mg/kg of extract & 15 mg/kg of fraction reduced MAP from 130–106.7 & 108 mmHg, respectively in salt-induced HTN ▪ 100 mg/kg extract reduced SBP from 177–154.1 mmHg in FIH	- 5-HT_2B_ receptor antagonism	(543)
		Aqueuse leaf extract	Diabetic rat isolated aorta	NA	*Ex vivo*	▪ 200 mg/kg partly counteract KCl- & NE-induced contractions in endothelium-intact aorta & enhanced Ach relaxation effect	- Endothelial PG synthesis	(544)
29	*Vernonia amygdalina*	Methanolic, ether, water & total crude leaf extracts	Rabbit isolated perfused heart	NA	*Ex vivo*	▪ 0.5−250 mg/kg reduced rate & force of heart contractility in a concentration-dependent manner	- Negative inotropic & chronotropic activity	(552)
		Methanol leaf extract	NaCl-induced HTN in wistar rats	PO	*In vivo*	▪ 400 mg/kg reduced SBP (190.17–87.17), DBP (132.83–63.5), MAP (151.5–70.5 mmHg), HR (467.83–427.83 beats/min)		(554)
		Aqueous leaf extract	Normotensive SDR rats & its isolated thoracic aorta	IV	*In vivo, Ex vivo*	▪ 10 mg/kg caused a bi-phasic alteration of BP (MAP of 73.7 rose to 101.9 in first phase before it fell to 60.2 in second phase) ▪ 2.7 mg/ml caused 31.3% relaxation of aorta	- Direct vasorelaxant mechanism	(556)
		95% & 50% ethanol extracts	Isolated rat aorta	NA	*Ex vivo*	▪ 95% ethanol extract exhibited vasorelaxant effect with EC_50_ of 6.78 mg/ml ▪ 50% ethanol extract exhibited vasorelaxant peffect with EC_50_ of 11.41 mg/ml	- Not studied	(557)
		Ethanol leaf extract & 95% ethanolic seed extracts	SHR rats, isolated rat aortic rings	PO	*In vivo, Ex vivo*	▪ 0.01−2.55 mg/ml of leaf extract caused dose-dependent relaxation in aortic rings in the intact- & denuded-endothelium ▪ 95% ethanol extract exhibited a stronger vasorelaxant effect	- Block VOCCs - K^+^-channels opening - M_3_ & β_2_-receptors activation - PGI_2_ & NO/cGMP pathways activation	(558)
30	*Zingiber officinale*	70% methanolic rhizome extract	Normotensive SDR rats, & isolated rat aorta, guinea pig atria & rabbit aorta	IV	*In vivo, Ex vivo*	▪ 0.3–3 mg/kg decreased BP, & force & rate of atrial contractions ▪ Exhibited a dose-dependent vasorelaxation in K^+^ & PE-induced contractions, with an EC_50_ of 0.11 mg/mL & 0.9 mg/mL, respectively	- VDCCs blockage	(563)
		Petroleum ether rhizome extract & its toluene fraction	FIH & DOCA salt-induced HTN in Wistar rats	PO	*In vivo*	▪ 50 mg/kg petroleum ether extract reduced SBP from 171.5–132.71 mmHg in FIH rats ▪ 50 mg/kg petroleum ether extract & 10 mg/kg toluene fraction significantly reduced MAP in salt-induced hypertensive rats	- 5-HT_2B_ & 5-HT_3_ receptors antagonism	(568)
		Decoction of rhizome	Guinea-pig isolated atrium & aortic strips	NA	*Ex vivo*	▪ Showed weak negative inotropic (42%) & chronotropic (8%) intrinsic activities ▪ Inhibited aortic contraction by 73%	- Calcium antagonistic activity	(569)
		Petroleum ether rhizomes extract & its n-hexane fraction	SHR rats & its isolated thoracic aortic rings	PO	*In vivo, Ex vivo*	▪ 250 mg/kg petroleum ether extract notably reduced SBP, MAP, & HR by 29 mmHg, 48.2 mmHg, & 57 beats/min, respectively ▪ n-hexane fraction produced notable relaxation effects in concentration-dependent manner	- NO & PGI_2_ release - cGMP-K^+^_ATP_-channels activation - M-receptors activation - CCB like activity - Ca^2+^ release from intracellular stores	(570)
		Aqueous crude extract	Normotensive SDR rats & its isolated aorta & isolated guinea-pig right atria	IV	*In vivo, Ex vivo*	▪ 10 mg/kg induced a fall in BP by 46.9% ▪ 0.01–5 mg/ml depressed PE-induced contraction of endothelium containing aorta ▪ 0.1–1.0 mg/ml relaxed K^+^-induced aorta contraction ▪ 0.1–5 mg/ml caused inhibition on force of atrial contractions in dose-dependent manner	- M-receptors activation - Ca^2+^-channels blockade	(571)
		Ginger crude extract of rhizome	Porcine coronary arteries	NA	*Ex vivo*	▪ 1, 3, 10, 30 & 100 μg/ml increased relaxation from 27–99% in endothelium-intact artery	- NOS signaling pathway - Vasoprotective activity	(2)

ROA, route of administration; MOA, mechanism of action; PO, per OS (orally); NO, nitric oxide; cGMP, cyclic guanosine monophosphate; 2k1C, two kidney one clip; SBP, systolic blood pressure; DBP, diastolic blood pressure; MAP, mean arterial blood pressure; HR, heart rate; PG, prostaglandin; SDR, Sprague-Dawley rats; L-NAME, L-nitroarginine methyl ester; SHRSP, stroke-prone spontaneously hypertensive rat; HTN, hypertension; eNOS, endothelial nitric oxide synthase; FIH, fructose-induced hypertension; HPOIH, heated oil-induced HTN; VCAM-1, vascular cell adhesion molecule-1; NA, not applicable; IV, intravenous; IP, intraperitonial; NC, not clear; SHR, spontaneously hypertensive rats; RBCs, red blood cells; H_2_S, hydrogen sulfide; ADH, antidiuretic hormone; BP, blood pressure; DOCA, desoxycorticosterone acetate; WKY, wistar kyoto rats; cAMP, cyclic adenosine monophosphate; PDEs, phosphodiestrases; CCB, calcium channel blocking; COX, cyclooxygenase; VGCCs, voltage-gated calcium channels; ET-1, endothelin-1; RAAS, rennin angiotensin aldosterone system; EC_50_, 50% effective concentration; VDCCs, voltage-dependent calcium channels; PE, phenylephenephrine; KCl, potassium chloride; EPI, epinephrine; IC_50_, 50% inhibitory concentration; ECs, endothelial cells; VSCCs, voltage-sensitive calcium channels; ROCCs, receptor-operated calcium channels; SNS, sympathetic nervous system; LDH, lactate dehydrogenase; RVH, renovascular hypertension; EDRF, endothelium-derived relaxing factor; NaCl, sodium chloride; NE, norepinephrine; 5-HT, serotonin; 1K1C, one kidney one clip.

**Table 4:** Antioxidant activity of medicinal plants claimed for treating hypertension in Ethiopia.

S. no.	Medicinal plants	Extract/treatment type(s)	Result(s)	Ref. no.
1	*Allium cepa*	5% dried onion diet	▪ Decreased TBARS in plasma & protect NO	(178)
		Aqueous onion bulbs extract	▪ 400 mg/kg reduced OS	(177)
2	*Allium sativum*	Aqueous garlic cloves extract	▪ 500 mg/kg significantly increased serum antioxidants level	(51)
		Aqueous garlic bulbsextract	▪ 150 mg/kg reduced OS	(177)
3	*Citrus aurantifolia*	Citrus leaf extract supplementation	▪ Reduced vascular damage	(237)
4	*Citrus limon*	5% lemon juice squeezed residue	▪ Significantly lowered urinary levels of 8-hydroxy-2’-deoxyguanosine & isoprostane	(250)
5	*Cymbopogon citratus*	Aqueous aerial part extracts	▪ 200 mg/kg significantly reduced OS	(9)
6	*Hibiscus sabdariffa*	80% methanolic leaf extract	▪ 400 mg/kg reduction serum OS	(298)
7	*Linum usitatissimum*	Flax lignan seed concentrate	▪ Reduced OS in a dose-dependent manner	(352, 353)
		Flaxseed peptide mixture	▪ 4 mg/ml caused 100% radical scavenging effect	(354)
8	*Mentha×piperita*	Powdered peppermint water extract	▪ Produced anti-oxidant effect	(373)
9	*Mentha spicata*	Spearmint water extract	▪ Produced anti-oxidant effect	(373)
		Ethanolic leaf extract	▪ Caused a dose-dependent anti-oxidant effect	(29)
10	*Moringa oleifera*	Aqueous leaf extract	▪ Reduced OS	(397)
11	*Nigella sativa*	Seed oil	▪ 2.5 mg/kg significantly reduced NADPH oxidase activity & increased HO-1 activity	(428)
		Seed oil	▪ 0.2 ml/kg attenuated oxidative injury	(430)
12	*Passiflora edulis*	Fruit pulp	▪ 8 g/kg significantly incresad GSH, & decreased TBARS	(448)
		70% ethanolic seed extract, & fruit juice & pulp extract	▪ 200 μg/mL reduced OS	(449)
13	*Persea americana*	Aqueuos extracts of leaf & seed	▪ Both extracts can scavenge ABTS radical	(465)
		Ethanol seed extract	▪ 500 mg/ml scavenged DPPH radical by 87.5, NO radical by 91.44%, & lipid peroxidation by 75.51%	(37)
14	*Ruta chalpensis*	70% ethanolic aerial parts extract	▪ scavenged DPPH radical significantly	(10)
15	*Vernonia amygdalina*	Methanol leaf extract	▪ 400 mg/kg increased anti-oxidant enzymes activity	(554)
16	*Zingiber officinale*	Ginger crude extract of rhizome	▪ Produced vasoprotective activity	(2)

TBARS, thiobarbituric acid reactive substances; NO, nitric oxide; OS, oxidative stress; HO-1, heme oxygenase-1; NADPH, nicotinamide adenine dinucleotide phosphate; GSH, glutathione; ABTS, 2,2-azino-bis-3-ethylbenzothiazoline-6-sulphonic acid; DPHH, 2,2-diphenyl-1-picrylhydrazyl.

**Table 5:** *In vitro* angiotensin converting enzyme inhibitory activity of medicinal plants claimed for treating hypertension in Ethiopia.

S. no.	Medicinal plants	Extract/treatment type(s)	Dose(s) and result(s)	Ref. no.
1	*Allium cepa*	Water & ethanol bulb extracts	▪ 0.33 mg/ml of each extract brings 52 & 50% ACEI effect, respectively	(181)
2	*Allium sativum*	Water leaves extract	▪ 0.33 mg/ml inhibited ACE by 30%	(205)
		Water & ethanol bulb extracts	▪ 0.33 mg/ml of each extract brings 76 & 68% ACEI effect, respectively	(181)
3	*Carica papaya*	Standardized methanolic leaf extract	▪ 100 mg/kg reduced ACE activity (by 72.9%)	(223)
4	*Citrus aurantium*	Water & ethanol fruit extracts	▪ 0.33 mg/ml of each extract brings 60 & 56% ACE inhibition effect, respectively	(181)
5	*Citrus aurantifolia*	Citrus leaf extract supplementation	▪ ACEI activity	(237)
		Water & ethanol peel extracts	▪ 0.33 mg/ml of each extract brings 67 & 35% ACE inhibition effect, respectively	(181)
6	*Citrus limon*	5% diluted lemon juice	▪ ACEI activity	(249)
7	*Coccinia grandis*	Hot water immature fruit extract	▪ Produced moderate activity against ACE (73.62% inhibition)	(265)
8	*Coriandrum sativum*	Water extract	▪ 1 mg/ml caused inhibition of ACE with IC_50_ value of 0.7 mg/ml	(38)
9	*Foeniculum vulgare*	Water seed extract	▪ 0.33 mg/ml exhibited 56% ACEI effect	(181)
		Water & ethanol root extracts	▪ 0.33 mg/ml of each extract brings 50 & 61% ACEI effect, respectively	
10	*Hibiscus sabdariffa*	Cold & hot aqueous calyces extracts	▪ Cold & hot extracts significantly reduced ACE, ang-II, & aldosterone levels in plasma	(294)
11	*Hordeum vulgare*	Alcoholic extract of barley seedling	▪ 5,000 μg/mL demonstrated potent (66.5%) ACEI effect	(323)
12	*Jatropha curcas*	Peptide fractions from seeds	▪ ACEI effect ranged from 22.66–55.83%	(328)
13	*Leucaena leucocephala*	Protein hydrolysate from seeds	▪ Globulin-Alcalase hydrolysate produced the highest (95.44%) ACEI activity	(339)
14	*Linum usitatissimum*	Flaxseed peptide mixture	▪ 0.4 mg/mL caused 70.8% ACE inhibition	(354)
15	*Lupinus albus*	Deoiled lupin, protein isolates, & their hydrosylates	▪ 1 mg/mL samples exhibited 2.6-58.7% ACE inhibitory effects	(361)
16	*Melia azedarch*	Petroleum ether, ethyl acetate, & methanol leaf extracts	▪ Extracts showed ACEI effect with IC_50_ value 536, 588 & 483 μg/ml, respectively	(368)
17	*Mentha×piperita*	Powdered peppermint water extract	▪ 600 μg caused 94% ACEI activity with IC_50_ value of 4.5 mg	(373)
18	*Mentha spicata*	Powdered spearmint water extract	▪ 600 μg caused 85.2% ACEI activity with IC_50_ value of 5.8 mg	(373)
19	*Moringa oleifera*	Leaves protein hydrolysate & ultrafiltration fractions	▪ 1 mg/ml of <1 kDa peptide fraction caused 84.71% ACE & 43.72% renin inhibitory activity	(8)
		Ethyl acetate leaf extracts	▪ Exhibited the highest ACEI effect (64.23%)	(400)
		Methanol leaf extract	▪ Inhibited ACE with an IC_50_ of 226.37 μg/ml	(401)
		95% ethanolic pods & aqueous leaf extracts	▪ 80 μg/mL of extracts showed >50% ACEI activity	(402)
20	*Nigella sativa*	Crude seed extract & ammonium sulphate fractions	▪ Crude extract exhibited 89% ACEI ▪ 30% ammonium sulphate fraction caused the highest (61%) ACEI effect	(427)
		Seed oil	▪ 2.5 mg/kg significantly reduced cardiac ACE activity	(428)
21	*Passiflora edulis*	Fruit juice & 96% ethanolic leaf extract	▪ 0.1 mg/ml of juice & ethanol extract showed ACEI effect by 40 & 27.4%, respectively	(453)
22	*Persea americana*	Methanol (95%) leaf extract & ethyl acetate fraction	▪ 100 μg/ml of crude extract & fraction inhibited ACE by 70 & 73. 6%, respectively	(463)
		Ethanol (95%) leaf extract	▪ 50 μl caused 29.49% ACE inhibitory activity	(464)
		Aqueuos extracts of leaf & seed	▪ 0–2.5 mg/ml inhibited ACE in a dose-dependent manner with EC_50_ of 1.57 mg/ml (leaf) & 1.03 mg/ml (seed)	(465)
		70% ethanolic leaf & nanoparticle extracts	▪ Leaf extract & nanoparticle extract inhibited ACE was by 60 & 59.5%, respectively	(466)
23	*Schinus molle*	Ethanol & acetone leaf extracts	▪ 0.33 mg/ml of the extracts caused 39 & 61% of ACEI activity, respectively	(501)
24	*Solanum nigrum*	95% ethanolic leaf extract	▪ 50 μl caused 29.52% ACEI effect	(464)
25	*Trigonella foenum-graecum*	Water & ethanol leaf extracts	▪ 0.33 mg/ml of extracts brings 55 & 23% ACEI effect, respectively	(181)
26	*Zingiber officinale*	Red & white ginger diet supplement	▪ 2% or 4% caused a notable ACEI effects with high effect by white ginger	(34)
		Water & ethanol root extracts	▪ 0.33 mg/ml of each extract brings 31 & 10% ACEI effect, respectively	(181)

ACE, angioteensin converting enzyme; ACEI, angioteensin converting enzyme inhibition.

**Table 6:** *In vivo* diuretic activity of medicinal plants claimed for hypertension treatment in Ethiopia.

S. no.	Medicinal plants	Extract/treatment type(s)	Study animal/tissue/assay	ROA	Dose(s) & result(s)	Remark(s)	Ref. no.
1	*Achyranthes aspera*	Methanolic extract of the whole plant	Wister albino rats	PO	▪ 400 mg/kg significantly increased urinary output (2.3 ml) & excretion of Na^+^, K^+^ & Cl^−^		(157)
		Aqueous seed extract	Goats	PO	▪ 3 g/kg resulted 52.7% increase in diuresis (significantly increased renal clearance of Na^+^, K^+^, Cl^−^, & HCO_3_^−)^		(158)
		Alcoholic & aqueous leaf extracts	Wistar rats	PO	▪ 100 mg/kg increased urine volume, & Na^+^, K^+^ & Cl^−^ ions excretion		(159)
2	*Ajuga remota*	80% methanolic & aqueous leaf extracts	Albino mice	PO	▪ 750 mg/kg methanol extract caused 91.4% urine, 69.6% Na^+^ & 76% Cl^−^ excretion ▪ 1,000 mg/kg aqueous extract produced 96% increase in urine volume, 69.2% Na^+^ loss & 88.6% Cl^−^ excretion	- Potassium-sparing diuretic activity	(164)
3	*Allium cepa*	Natural juice	SDR rats	PO	▪ 400 mg/kg increased urinary excretion by 103% with 1.27 diuretic action		(173)
		n-butanol bulb extract	Westar albino rats	PO	▪ 20 mg/kg produced 9.9 ml volume of urine & 26.84% increase in Na^+^ excretion	- Aquatic & natriuretic responses	(32)
4	*Allium sativum*	Bulb powder packed in capsules	Anaesthetized dogs	PO	▪ 2.5−15 mg/kg decreased arterial BP ▪ 15 & 20 mg/kg provoked bradycardia	- Diuretic activity	(185)
		n-butanol bulb fraction	Wistar albino rats	PO	▪ 20 mg/kg produced 9.3 ml of urine & increased Na^+^ excretion by 24.57%	- Natriuretic & aquaretic response	(208)
		Chromatographically purified garlic fractions	Rabbits	IV	▪ 2, 4 & 6 μg/kg of fraction V elicit a dose-dependent increased in diuresis & natriuresis ▪ 6 μg/kg of fraction V decreased HR by 10%	- Active transport inhibition - Stimulation of ADH, ang-II, & aldosterone effect on Na^+^ transport	(209)
		Purified garlic fraction	Dogs	IV	▪ 6 μg/kg produced biphasic diuretic–natriuretic response	- Sodium pump inhibition at sodium tubular reabsorption level of the kidney	(210)
			Isolated rat kideny		▪ 200 μ/ml produced 70% *in vitro* inhibitory effect on Na^+^/K^+^-ATPase in the kidney		
5	*Carica papaya*	Aqueous seed extract	SDR rats	PO	▪ 400 & 800 mg/Kg increased urine volume & excretion of Na^+^ & K^+^ ions		(221)
6	*Citrullus lanatus*	Ethanolic pulp extract	Wistar rats	IP	▪ 200 mg/kg increased urinary output (3.63 ml), & urinary Na^+^ (1.85 mmol) & Cl^−^ (1.08 mmol) levels		(226)
		Pure & homogenized watermelon juice	Mice	PO	▪ Produced 200 μl mean amount of urine		(228)
7	*Citrus limon*	Pure lemon juice	Mice	PO	▪ 1 ml/kg produced moderate diuretic effect (1.29 ml of urine)		(13)
8	*Citrus medica*	Methanolic leaf extract	Wistar albino rats	PO	▪ 200 mg/kg produced 1.29 diuretic index, 2.85 ml urine volume, & 113.28 ml urine excretion		(257)
9	*Coccinia grandis*	Aqueous & alcoholic fruit extracts	Albino rats	PO	▪ 100 mg/kg of aqueous extract produced 5.2 ml urine volume ▪ 100 mg/kg of alcoholic extract produced 4.1 ml urine volume		(264)
10	*Coriandrum sativum*	70% methanolic fruit extract	Normotensive SDR rats	NA	▪ 100 mg/kg produced 6.47 ml urine out put		(267)
		Aqueous seed extract	Anesthetized wistar rats	IV	▪ 100 mg/kg significantly increased water (48.6 μl/min), Na^+^ (8.6 μ mol/min), & Cl^−^ (46.1 μ mol/min) excretion		(270)
		Aqueous seed extract	Rats	PO	▪ 10% decoction produced high diuretic activity (273 ml mean urinary output)		(271)
		Methanol & aqueous leaf extracts	Wistar albino rats	PO	▪ 400 mg/kg produced 14.2 & 10.6 ml urine, respectively, with high Na^+^ excretion than K^+^		(272)
		70% methanol seed extract	Swiss albino mice	IP	▪ 400 mg/kg produced 51% diuretic activity with urine output 5.3 ml/6 h		(273)
11	*Croton macrostachyus*	80% methanol & aqueous leaf extracts	SDR rats	PO	▪ 400 mg/kg of aqueous extract caused 78.8% Na^+^, 199.9% K^+^ & 72.9% Cl^−^ loss in urine ▪ 400 mg/kg of methanol extract caused K^+^ (193.1%) & Cl^–^ (67.2%) loss, with a lesser Na^+^ excretion	▪ Natriuresis & kaliuresis	(275)
12	*Cymbopogon citratus*	Aqueous aerial part extracts of C. citratus	Salt & ethanol-induced HTN in Wistar rats	PO	▪ 200 mg/kg increased (1.12) urine volume		(9)
13	*Foeniculum vulgare*	Aqueous root extract	Rats	PO	▪ 10% decoction produced intermediate diuretic activity (150 ml mean urinary output)		(271)
		70% methanol seed extract	Mice	IP	▪ 400 mg/kg produced 53% diuretic activity with urine output 4.6 ml/6hr		(273)
		Aqueous fruit extract	SHR rats	PO	▪ 190 mg/kg significantly decreased SBP & increased in Na^+^ & K^+^ renal excretion	▪ Natriuretic activity	(291)
		Hydroalcohol root extract	SDR rats	IP	▪ 200 mg/kg significantly increased excretion of urine with a maximum of 7.7 ml/100 g		(292)
14	*Hibiscus sabdariffa*	Aqueous & 95% ethanol calyces extracts	Rats	PO	▪ 10% decoction produced high diuretic activity (234 ml mean urinary output) ▪ 500 mg/kg ethanolic extract increased urinary excretion by 124.4%		(271)
		Aqueous flower petals extract	Wistar rats	PO	▪ 700 mg/kg induced 29.42 ml/kg increased in urinary excretion & resulted in 72.51% Na^+^, 76.54% K^+^ & 58.81% Cl^−^ ion excretion		(315)
15	*Mentha×piperita*	Methanolic arial parts extract	Wistar albino rats	IP	▪ 100, 300, & 500 mg/kg increased urine output & urinary excretion of Na^+^ & K^+^		(378)
16	*Mentha spicata*	Ethanolic leaf extract	Wistar albino rats	PO	▪ 200 mg/kg produced 0.77 diuretic, 188.01 saluretic, & 2.17 natriuretic effects	▪ Carbonic anhydrase inhibition	(29)
17	*Moringa oleifera*	95% ethanolic leaf extract	SHR rats	PO	▪ 1,000 mg/kg caused diuretic effects		(402)
		Hot water infusions of seeds	Albino rats	PO	▪ 1,000 mg/kg produced 13.08 ml urine output		(403)
		70% alcoholic leaf extract	Swiss albino rats	PO	▪ 50 mg/kg produced highest natriuretic effects ▪ 200 mg/kg brings maximum saluretic effects		(404)
		96% ethanolic seed & pod extracts	Swiss albino mice	NC	▪ 400 mg/kg pod extract produced highest (5.94 ml) volume of urine ▪ Both extracts increased Na^+^, K^+^, Cl^−^ excretion	▪ May act as loop diuretics	(405)
18	*Moringa stenopetala*	Microencapsulated product of leaf extract	Swiss albino mice, Wistar rats	PO	▪ 1,000 mg/kg caused 56% urinary excretion with significant excretion of Na^+^, K^+^, & Cl^−^ ions		(409)
		70% ethanol leaf extract	Swiss albino mice	PO	▪ 500 mg/kg produced significant urine output (2.5 ml) & highest urinary excretion (7.78%) ▪ 100 mg/kg significantly increased K^+^ excretion ▪ 250 & 350 mg/kg caused significant loss of Na^+^ & Cl^−^ ions ▪ 250 mg/kg caused highest saluretic activity		(410)
		Aqueous crude extract & hot tea infusion of leaves	Wistar rats	PO	▪ 125 mg/kg aqueous extract & 2 tea spoonful infusion displayed the highest diuresis (101% & 96%, respectively) ▪ Both test samples revealed a significant Na^+^ urinary excretion at all doses ▪ 125, 250, & 500 mg/kg of extract, & 1 & 2 tea spoonful infusion caused significant Cl^−^ urinary excretion ▪ 500 mg/kg extract & 4 & 6 tea spoonful infusion caused significant K^+^ excretion		(406)
19	*Nigella sativa*	Dichloromethane seed extract	SHR rats	PO	▪ 0.6 ml/kg increased diuresis by 16% & enhanced urinary excretion of Na^+^, K^+^, & Cl^−^ ions		(431)
		Aqueous seed extract	Albino rats	IP	▪ 50 mg/kg showed 46% diuretic activity	▪ Natriuretic & kaliuretic effects	(41)
		Seeds oil	Lithiasic wistar rats	PO	▪ 5 ml/kg produced 27.83 ml maximum urine output		(432)
20	*Passiflora edulis*	Methanolic leaf extract	Rats	IP	▪ 400 mg/kg produced significantly different uolume of urine than the control		(454)
21	*Persea americana*	70% ethanolic leaf & nanoparticle extracts	NaCl-induced wistar rats	PO	▪ Crude extract & nanoparticle extract produced 1.41 & 2.25 diuretic index, respectively		(466)
		Ethanol seed extract	Wistar albino rats	NC	▪ 500 mg/ml increased urine output (5.25 ml) & urinary excretion of Na^+^, K^+^ & Cl^−^ ions		(37)
		70% ethanol leaf extract	Wistar rats	PO	▪ 150 mg/kg produced 3.32 ml total urine output		(467)
22	*Rosmarinus officinalis*	Aqueous leaf extract	Wistar rats	PO	▪ 8% (8 g in 100 ml distilled water) produced significant urinary excretion of Na^+^, K^+^ & Cl^−^		(472)
23	*Rumex abyssinicus*	Aqueous & 80% methanolic rhizome extracts	Albino mice	PO	▪ 1,000 mg/kg aqueous & 750 mg/kg methanol extract markedly increased urine volume (2.7; 2.8 ml) & excretion of Na^+^ (93.13; 106 mmol), K^+^ (55.2; 55.19 mmol) & Cl^−^ (114.13; 111.75 mmol/L), respectively	▪ Saluretic type activity	(476)
		Aqueous, methanol & ethyl acetate fractions of crude rhizomes extract	Albino rats	PO	▪ 750 mg/kg of all fraction produced significant urine out put & urinary electrolyte excretion with more saluretic effect by methanol fraction	▪ Furosemide like activity	(46)
24	*Tamarindus indica*	Aqueous leaf extract	Rats	PO	▪ 10% decoction produced high diuretic activity (442 ml urinary output)		(271)
		Aqueous ripe fruit pulp extract	Wistar rats	PO	▪ 1,200 mg/kg significantly increased urine volume (14.50 ml) & urinary excretion of K^+^ (139.42 mmol/L) & Cl^−^ (207.32 mmol/L)		(529)
25	*Thymus schimperi*	Aqueous leaf extract & essential oil extracts	Salt-sucrose induced HTN rats	PO	▪ 500 mg/kg extract produced significant prevention in SBP increment & highest (5.86) diuretic index (2.46 ml urine volume) ▪ 750 & 10,000 mg/kg significantly increased Na^+^, K^+^ & Cl^−^ excretion ▪ 1 &1.5 ml/kg essential oils howed significant kaliuretic effect		(54)
26	*Thymus serrulatus*	80% methanolic leaf extract & n-butanol fraction	Swiss albino mice	PO	▪ 500 mg/kg of crude extract produced 88% diuretic effect ▪ 1,000 mg/kg fraction displayed 104% diuretic effect with a good natriuretic (1.34) activity		(30)
27	*Trigonella foenum-graecum*	Petroleum ether, benzene, ethanol & aqueous seed extract	Wister rats	IP	▪ 150 & 350 mg/kg of all extract significantly increased urine volume & electrolyte excretion		(547)
28	*Vernonia amygdalina*	Standard aqueous leaf extract	Wistar rats	IP	▪ 10 mg/100 g caused 1.465 ml/rat/hour mean urine output		(47)
29	*Zingiber officinale*	Ethanolic & aqueous rhizomes extracts	Wistar albino rats	PO	▪ 500 mg/kg ethanol extract caused significant water & Na^+^ excretion (4.16 ml urine volume) than aqueous extract (3.83 ml urine volume)		(573)

ROA, route of administration; PO, per OS (orally); NA, not applicable; IV, intravenous; IP, intraperitonial; NC, not clear; SHR, spontaneously hypertensive rats; SDR, Sprague-Dawley rats; ADH, antidiuretic hormone; BP, blood pressure; HR, heat rate; SBP, systolic blood pressure; NaCl, sodium chloride.

**Table 7:** Clinical studies on antihypertensive activities of medicinal plants claimed for hypertension in Ethiopia.

S. No.	Medicinal Plant	Extract/Treatment type	Study design	Patient condition	Dose	Duration	Result(s)	Ref.
1	*Allium cepa*	Quercetin-rich onion skin extract	Double-blinded, placebo-controlled cross-over trial	Pre-HTN & stage-1 HTN	162 mg/d quercetin	6-week	Decreased 24 h ambulatory SBP by 3.6 mmHg	(176)
2	*Allium sativum*	Garlic tablets	Single-blind, placebo controlled trial	Stage 1 essential HTN	1500 mg/day	24 weeks	Reduction in SBP by 7.6 mmHg & in DBP by 6.27 mmHg	(190)
		500 mg garlic powder capsules	Single-blind, placebo-controlled, parallel RCT	Hypertensive-diabetic patients	2 g/day	40 days	Reduced SBP from 171.88–138.75 & DBP from 106.88–78.75 mmHg	(191)
		500 mg garlic & coriander mixture powder capsules	>>	>>	>>	>>	Reduced SBP from 176.88–148.13 & DBP from 96.25–86.25 mmHg	(191)
		Garlic powder	Triple-blind clinical trial	Primary HTN	10 g garlic powder	20 days	Increased patient comfort & decreased SBP (153.78–138.33) & DBP (97.27–90.30 mmHg)	(15)
		Processed garlic with 75.3 mg/100 g of *S*-allyl-ʟ-cysteine	Placebo-controlled parallel feeding RCT	Pre-hypertensive or hypertensive patients	Two 500 mg capsule/day	8 weeks	Lowered SBP by 8.05 mmHg	(192)
		960 mg aged garlic extract capsules containing 2.4 mg S-allylcysteine	Double-blind, parallel, placebo-controlled RCT	Uncontrolled HTN	4 capsule/day	12 weeks	Decrease SBP by 10.2 mmHg & showed 92% tolerability & acceptability	(193)
		480 mg of aged garlic extract containing 1.2 mg of S-allylcysteine	Double-blind, placebo-controlled dose–response RCT	Uncontrolled systolic HTN	2 capsule per day	12 weeks	Lowered SBP by 11.8 mmHg & showed 93% tolerability, compliance & acceptability	(196)
		650 mg garlic bulb tablets containing allyl disulfide, alliin, & diallyl trisulfide	Placebo-controlled, crossover trail	Mild HTN	4 tablet/day	10 days	Produced significant lowering of SBP but not DBP	(200)
		Garlic tablet plus 1100 mg vitamin-C/& E tablets	>>	>>	>>	>>	Lower both SBP & DBP to reference ranges	(200)
3	*Citrullus lanatus*	Watermelon seeds	Open labeled, single-arm trial	Patients with SBP 120-159 & DBP 80-99 mmHg	50 g/day	40 days	Produced significant decrease in SBP by 13-16 mmHg & DBP by 8-10 mmHg	(227)
		Watermelon extract supplement	Double-blind, experimental & placebo-controlled RCT	Pre-hypertensive & hypertensive patients	6 g/day	6 weeks	Produced significant reduction in SBP (137.8–126.0 mmHg) & DBP (79.2–72.3 mmHg)	(232)
		Watermelon supplement with L-citrulline & L-arginine in 2:1 ratio	Two-period, crossover RCT	Stage-1 HTN	6 g/day	6 weeks	Ankle & brachial SBP decreased by 11.5 & 15.1 mmHg, & DBP by 7.8 & 7.6 mmHg, respectively	(233)
4	*Citrus limon*	Lemon juice	Descriptive & transversal	Hypertensive patients	30 ml	30 min	Averagely decreased SBP by 10 mmHg & DBP by 6 mmHg	(253)
			Triple-blind clinical trial	Primary HTN	10 cc lemon juice	20 days	Increased patient comfort & decreased SBP (153.48–143.18) & DBP (96.96–91.51 mmHg)	(15)
5	*Coriandrum sativum*	500 mg coriander powder capsules	Single-blind, placebo-controlled RCT	Hypertensive-diabetic patients	2 g/day	40 days	Reduced SBP from 175.63–156.88 & DBP from 100–83.75 mmHg	(191)
6	*Cymbopogon citratus*	Lemongrasses tea of leaf powder	Clinical study	Normotensive adults	2, 4 & 8 g/day	30 days	4 g decreased SBP & DBP while 8 g decreased MAP & HR	(45)
		Infusion of leaf powder	Pre-& post experimental design	Healthy volunteers	2, 4 & 8 g/day	30 days	Significantly increased natriuretic & saliuric indices	(281)
7	*Hibiscus sabdariffa*	Dry calyx decoction standardized on 9.6 mg anthocyanins	Controlled & randomized clinical trial	Mild to moderate HTN not received medication	Taking infusion daily	4 weeks	Showed diuretic activity & a decrease in BP from 139.05/90.81–123.73/79.52 mmHg	(302)
		Calyces decoction	Multi-center before-after pilot clinical trial	Uncontrolled HTN, with (without) medication	10 g/0.5l, 15 g/1l, & 20 g/1l/day	4 weeks	65% participants saw their SBP decreased by 10 mmHg & 38% of participants reached target BP	(303)
			Multi-centric pilot comparative intervention	Uncontrolled HTN, with (without) medication	10 g, 15 g, & 20 g/day	6 weeks	61.8% participants attain target BP with mean reduction of 23.1 in SBP & 12 mmHg in DBP	(305)
		Decoction (sour tea)	Sequential RCT	Essential HTN	2 spoonfuls of tea	12 days	Showed 11.2% lowering of SBP & 10.7% decrease in DBP in the experimental group	(306)
		Galenic forms of tablet (bissap tablets) & brew (decoction) of calyces	Multi-centric RCT	Hypertensives with SBP between 140 & 180 mmHg &/or DBP between 90 & 110 mmHg	2 bissap 375 mg tablet per day & bissap 10 g brew (calyx) daily	6 months	Produced a decrease in SBP by 19.5 mmHg, with slightly more effect by brews than tablets; bissap showed 75% rate of clinical effectiveness	(307)
		Sour tea	RCT	Stage 1 HTN	2 cup sour tea (480 ml) daily	1 month	Significantly higher reduction in SBP (by 7.43 mmHg) in the intervention group	(308)
		Dried calyxes standardized on 250 mg total anthocyanins	Double-blind RCT	Stage 1 or 2 HTN	Dissolved in 250 ml water & taken daily	4 weeks	Showed ACEI effect & decreased BP from 146.48/97.77–129.89/85.96 mmHg	(312)
8	*Linum usitatissimum*	Milled flaxseed in diet	Double-blinded, placebo-controlled RCT	Peripheral artery disease patients	30 g/day	Over 6 months	SBP lowered by 10 mmHg, & DBP lowered by 7 mmHg	(348)
9	*Mentha piperita*	Leaf decoction	Case report	Hypertensive cases	4–12 g (3–9 teaspoonfuls) daily	Not clear	Fall in elevated BP	(376)
		Peppermint juice	Clinical study	University students	200 ml twice/day	30 days	Showed a decrease in BP in 52.5% of study participants	(377)
10	*Moringa oleifera*	Leaves juices	Experimental study	Stage 1 HTN	150 ml BID per day	30 days	Reduced mean SBP from 140–111 & DBP from 97–79 mmHg	(385)
		Leaf decoction	Pilot study	Spontaneous HTN	20 g/750 ml per day	5 weeks	Both SBP & DBP reduced down to the near normal limits	(390)
		Cooked leaves	Prospective, placebo-controlled clinical study	Healthy participants	120 g/day	1 week	Significantly decreased SBP & DBP	(391)
		Aqueous leaf extract	Clinic based-observational study	Normotensive adults	57, 28.5, & 85.7 mg/kg/250 ml	90 min	Produced a fall in intraocular pressure & BP in a dose-dependent manner	(392)
		Leaf powder as diet supplement	Clinical study	Normal & obese hypertensive patients	30 g daily	6 months	Reduced SBP & DBP by 6.86 & 14.63 mmHg in obese & by 10.53 & 6.23 mmHg in normal weight subjects, respectively	(393)
11	*Nigella sativa*	Standardized seed extract capsules	Double-blind, placebo-controlled RCT	Mild hypertension	100 mg or 200 mg twice a day	8 weeks	SBP & DBP of the test groups were significantly lower than the baseline levels	(415)
		Seed oil	Double-blind, placebo-controlled RCT	Healthy volunteers	5 ml/day	8 weeks	Caused 8.17% reduction in SBP & 12.46% reduction in DBP	(416)
		Seeds virgin oil	Clinical study	Mild to moderate HTN	0.5 ml twice a day	180 days	Decreased SBP & DBP from 155.07–127.28 mmHg & 94.3–76.35 mmHg, respectively	(417)
		Seed oil	Quasi-expermental research with pretest-posttest control group	Hypertensive adults	50 mg/day	3 months	Produced significant effect in reducing SBP & DBP	(418)
		Seeds powder	Open label RCT	MS patients with HTN	500 mg capsule/day	8 weeks	Reduced SBP from 166.78–130.85 mhg & DBP from 88.02–80.84 mmHg	(419)
		Paste mixture of *N. sativa* seeds & honey	Clinical study	Hypercholesterolemic & healthy subjects	50 mg/kg/day	3 months	Reduced SBP by 3.9% & DBP by 5.5% in hypercholesterolemic subjects	(420)
12	*Passiflora edulis*	Fruit juice extract	Clinical study	Non-chronic hypertensive	2 g/day in 2 ml	30 days	Reduced SBP (142.4–125.2) & DBP (79–76.1 mmHg)	(443)
		Fruit peel extract	Randomized, double-blind, placebo-controlled trial	Adult T2DM subjects	220 mg/day	16 weeks	Reduced mean SBP from 133.8–126.1 mmHg	(444)
		Fruit peel extract	Randomized, parallel-group, double-blind, placebo-controlled trial	Stage 1/2 essential HTN	400 mg/day	4 weeks	Decreased SBP & DBP significantly by 30.9 & 24.6 mmHg, respectively	(447)
13	*Tamarindus indica*	Dried & pulverized fruit pulp	*In Vivo* Approach	Human Model	15 mg/kg twice a day	4 weeks	Significantly reduced DBP	(530)
14	*Zingiber Officinale*	Rhizomes extract	Randomized safety trial	Healthy humans	100 mg/kg per day	2 & 4 h	Reduced SBP (114.3–105.5 afer 2 h & 114.3–107.6 mmHg after 4 h), DBP (73.3–70.5 mmHg after 2 h) & HR (78.8–70.2 beats/min after 2 h)	(560)

HTN, hypertension; RCT, randomized control trial; SBP, systolic blood pressure; DBP, diastolic blood pressure; MS, metabolic syndrome; MAP, mean arterial blood pressure; HR, heart rate; BP, blood pressure; ACEI, angiotensin coverting enzyme inhibition.

**Table 8:** Preclinical studies on secondary metabolities isolated from medicinal plants claimed for hypertension in Ethiopia.

S. no.	Secondary metabolites	Sources	Study model(s)	Study subject(s)	Dose and result(s)	Possibly MOA(s)	Ref. no.
1	Saponins	*Achyranthes aspera*	*In vivo*	Rats	▪ 10 mg/kg increased urine output comparable to acetazolamide effect	- Diuretic activity	(578)
2	Crude flavonoids, fraction B & flavonoid glycosides	*Citrus limon*	*In vivo, In vitro*	Hypertensive rats & ACEI assay	▪ Both crude flavonoids & fraction B significantly lowered SBP ▪ Both crude flavonoids & flavonoid glycosides caused ACEI	- ACEI activity	(249)
3	Purified polyphenolic fraction	*Coccinia grandis*	*In vitro*	ACEI assay	▪ Produced 20.12% inhibitory effect against ACE	- ACEI activity	(265)
4	Flavonoid rich leaf fraction	*Coriandrum sativum*	*In vivo, ex vivo, in vitro*	L-NAME intoxicated albino rats, isolated rat heart, rabbit lung, & DPPH assay	▪ 400 mg/kg prevented a decline in serum NO level ▪ 100 μg/ml showed 81.4% inhibition effect against ACE	- Anti-oxidant activity - ACEI activity - Enhancing NO levels	(274, 594)
5	Flavonoids & tannins	*Cymbopogon citratus*	*Ex vivo*	Distal segements of human internal thoracic arteries	▪ 0.2 mg/ml flavonoids caused a notable inhibition against NE-mediated vasoconstriction ▪ 0.002−0.2 mg/ml tannin caused 26.91% maximal vasorelaxation	- Not clear	(284)
6	Phenolic compounds	*Foeniculum vulgare*	*In vitro*	ACEI assay	▪ Produced 50.8% ACEI effects	- ACEI activity	(290)
7	Alkaloidal salts of water leaf extract	*Moringa oleifera*	*Ex vivo*	Isolated frog heart, guinea pig taenia coli	▪ 3−48 ng concentration produced negative inotropic effect	- CCB activity	(613)
8	Steroidal triterpenes	*Schinus molle*	*In vitro*	ACEI assay	▪ Showed ACEI effect with IC_50_ of 250 μM	- ACEI activity	(501)
9	Saponin rich fraction of seeds	*Trigonella foenumgraecum*	*In vivo*	1K1C rats	▪ 100 & 200 mg/kg showed a dose-dependent anti-HTN effects	- Not studied	(642)
10	Free phenol & bound phenol leaf extracts	*Vernonia amygdalina*	*In vitro*	ACEI & anti-oxidant assays	▪ 5–20 mg/ml caused ACEI effects in a dose-dependent manner	- Anti-oxidant activity - ACEI activity	(646)

MOA, mechanism of action; SBP, systolic blood pressure; NO, nitric oxide; ACE, angiotensin coverting enzyme; ACEI, angiotensin coverting enzyme inhibition; L-NAME, L-nitroarginine methyl ester; DPHH, 2,2-diphenyl-1-picrylhydrazyl; NE, norepinephrine; CCB, calcium channel blocking; IC50, 50% inhibitory concentration; 1K1C, one kidney one clip.

**Table 9:** Preclinical studies on pure compounds isolated from medicinal plants claimed for hypertension in Ethiopia.

S. no.	Active compound(s)	Sources	Study model(s)	Study subject(s)	Dose and result(s)	Possibly MOA(s)	Ref. no.
1	Achyranthine	*Achyranthes aspera*	*In vivo*	Dogs & frogs	▪ Decreased BP & HR ▪ Dilate blood vessels	- Negative chronotropy - Vasodilation	(579)
2	Quercetin	*Allium cepa* & *Allium sativum*	*In vivo, ex vivo,* anti-oxidant assay	SHR rats, thoracic aortic rings, & superior mesenteric artery	▪ 10 mg/kg induced a reduction in SBP, DBP, & MAP by 18, 23, & 21%, respectively	- Anti-oxidant activity	(580)
			*In vivo, In vitro*	SDR rats	▪ 0.5% level depressed HTN strongly ▪ Suppressed the decrease in NO metabolites in plasma & urine ▪ Decreased lipid peroxidation in plasma	- Increased NO level - Anti-oxidative activity	(587)
3	Pure allicin	*Allium sativum*	*In vitro*	Rat isolated pulmonary arteries	▪ 1.0 g/ml brings 62% vasorelaxation	- NO formation activation	(201)
4	Citral	*Cymbopogon citratus*	*Ex vivo*	Isolated hypertensive rat aortic rings	▪ 0.00624−6.24 mM caused a dose-dependent vasorelaxation with 39*.*13% maximum effect	- Affect intracellular Ca^2+^ concentration - Partially via NO pathway	(596)
5	Hibiscus acid & garcinia acid	*Hibiscus sabdariffa*	*Ex vivo*	Isolated thoracic & abdominal rat aorta	▪ 1 mg/ml relaxed PE pre-contracted tissue by 96% ▪ 2 mg/ml relaxed KCl pre-contracted tissue by 77%	- Blockade of VDCCs	(310)
6	Delphinidin-3-O-samb-ubioside & cyanidin-3-O-sambubioside	*Hibiscus sabdariffa*	*In vitro*	ACEI assay	▪ 200 μg/ml of each compound showed ACEI effects with an IC_50_ of 84.5 & 68.4 μg/ml, respectively	- ACEI activity	(316)
7	Isoorientin	*Hordeum vulgare*	*In vitro*	ACEI assay	▪ Exhibited ACEI effects with an IC_50_ of 7.3 μM	- ACEI activity	(323)
8	Secoisolariciresinol diglucoside	*Linum usitatissimum*	*In vivo*	Anesthetized SDR normotensive rats	▪ 20 mg/kg caused fall in SBP, DBP, & MAP by 40, 48, & 43%, respectively	- GC activation	(610)
9	Quercetin-3-O-glucoside	*Moringa oleifera*	*In vitro*	ACEI assay	▪ 28 μg/ml produced 75.74% ACEI effect	- ACEI ativity	(400)
10	4-1 (4’-O-acetyla-α-L-rhamnosyloxy) benzyl] isothiocyanate, Niaziminin A, & Niaziminin B	*Moringa oleifera*	*In vivo*	Normotensive rats	▪ 3 mg/kg of isothiocyanate, niaziminin A, & niaziminin B decreased MAP by 34.9, 37.5 & 40.3%, respectively	- Hypotensive activity	(614)
11	Niazinin A, Niazinin B, Niazimicin, & Niaziminin A + B	*Moringa oleifera*	*In vivo, Ex vivo*	Rats, isolated rabbit aorta & guinea-pig atria	▪ 1−10 mg/kg of all compounds caused a dose-dependent fall in SBP & DBP ▪ 50-150 μg/ml of all compounds caused negative inotropic & chronotropic effects	- Hypotensive & bradycardic activity via muscarnic receptor activation	(389)
12	β-sitosterol*,* methyl *p-*hydroxybenzoate, & *p-*hydroxybenzaldehyde	*Moringa oleifera*	*In vivo*	Anaesthesized normotensive Wistar rats	▪ 3 mg/kg of β-sitosterol & 10 mg/kg of the other two compounds reduced MAP by 43.05, 47.01, & 75.05%, respectively	- Hypotensive activity	(394)
13	α-pinene & *p*-cymene	*Nigella sativa*	*In vivo*	Rats	▪ 2−16 µl/kg decreased both arterial BP & HR	- Suppresing sympatheic outflow at vasomotor centre in the medulla	(425)
14	Thymoquinone	*Nigella sativa*	*In vivo*	Guinea-pigs	▪ 6−24 mg/kg decreased arterial BP & HR in a dose-deppendent manner	- 5-hydroxytrptaminergic & muscarinic actions	(426)
		*Nigella sativa*	*In vivo*, *in vitro*	L-NAME-induced HTN in Wistar rats, anti-oxidant assays	▪ 0.5 & 1 mg/kg reduced SBP & inhibited the production of superoxide radicals in a dose-dependent manner	- Anti-oxidant activity	(617)
		*Nigella sativa*	*Ex vivo*	Isolated rat pulmonary arterial rings	▪ 50–1,000 μM caused a concentration-dependent vasorelaxation the artery precontracted by PE	- K_ATP_-channels activation - Blocking 5-HT-, α_1_- & ET- receptors	(618)
15	Luteolin	*Passiflora edulis*	*In vivo*	Hypertensive rats	▪ 50 mg/ml significantly decreased BP	- Vasodilatory effect	(445)
16	Anthocyanin fraction & edulilic acid	*Passiflora edulis*	*In vivo*	Hypertensive rats	▪ 2.39 mg/kg anthocyanin & 1.19 mg/kg edulilic acid significantly decreased SBP, DBP, & MAP	- Unknown	(446)
17	Scirpusin B & piceatannol	*Passiflora edulis*	*Ex vivo, in vitro*	Isolated rat thoracic aorta	▪ 30 μM of both compounds showed vasorelaxation effect	- Endothelium-derived NO production	(621)
18	Carnosic acid & carnosol	*Rosmarinus officinalis*	*Ex vivo*	Isolated rat thoracic aorta	▪ 0.05−0.80 mg/mL of each ingredients showed a dose-dependent vasorelaxant activity	- Increasing NO level - Decreasing ang-II level	(471)
19	Tilifodiolide	*Salvia tiliifolia*	*Ex vivo*	Isolated rat aortic rings	▪ 0.9–298 μM exhibited vasorelaxant effects with EC_50_ of 48 μM	- Mediated by NO & cGMP	(628)
20	Rosmarinic acid	*Satureja punctata*	*In vivo*	Normotensive guinea pigs	▪ 3 mg/kg caused 31.78% fall in MAP	- Not studied	(489)
21	10-gingerol, 8-gingerol, & 6-gingerol	*Zingiber officinale*	PKC & *Pa* tests	Bioinformatics approach	▪ Have good vasoprotective, vasodilator (*Pa* > 7), & anti-oxidant potential	- AT_1_-receptor antagonist activity	(648)
22	8-gingerol, 6-gingerol, & 6-shogaol	*Zingiber officinale*	*Ex vivo*	Isolated guinea-pig atrium, guinea-pig aortic strips	▪ All compounds showed negative inotropic effect ▪ Only 6-shogaol showed negative chronotropy (50% at 10 mg/ml)	- Calcium antagonistic activity	(569)
23	10-gingerol, 8-gingerol, & 6-gingerol	*Zingiber officinale*	*Ex vivo*	Isolated rat aorta	▪ 1.0–300 mg/ml of all gingerols exhibited vasodilation in PE-pre-contracted aorta	- Ca^2+^-antagonist activity	(571)

MOA, mechanism of action; BP, blood pressure; SHR, *spontaneously hypertensive rats*; *SDR*, Sprague-Dawley rats; SBP, systolic blood pressure; DBP, diastolic blood pressure; MAP, mean arterial blood pressure; HTN, hypertension; NO, nitric oxide; ACE, angiotensin coverting enzyme; ACEI, angiotensin coverting enzyme inhibition; L-NAME, L-nitroarginine methyl ester; *PE*, *penylepinephrine*; *KCl*, *potassium chloride*; *VDCCs*, *voltage-dependent calcium channels*; *GC*, guanylyl cyclases; EC_50_, 50% effective concentration; IC_50_, 50% inhibitory concentration; cGMP, cyclic guanosine monophosphate; PKC, pharmacokinetic.

Although *Catha edulis* is traditionally considered an anti-HTN plant (57, 77) in Ethiopia (Supplementary material 1), several studies suggest that khat is more likely to raise BP than lower it (143–147). These findings highlight the possibility that some herbs and plants might have effects contrary to their traditional uses. Therefore, scientifically validating the medicinal claims of such plants is crucial for ensuring patient safety.

Herbal medicines help manage and reduce HTN through various mechanisms, including antioxidant, antiinflammatory, antiproliferative, and antiapoptotic effects. They also stimulate the endothelial nitric oxide synthase (eNOS)/NO signaling pathway, decrease endothelial permeability, inhibit ACE activity, increase diuresis, regulate Ca^2+^ levels in VSMCs or myocardial cells, inhibit the expression or activity of contractile and structural proteins, and open K^+^ _ATP_-channels. Natural plants or herbs can reduce inflammation in macrophages and monocytes by inhibiting the inducible NOS (iNOS)/NO signaling pathway, potentially through the activation of estrogen receptor and PPARα-dependent signaling pathways (148). The mechanisms through which Ethiopian MPs (their extracts and active compounds) control HTN are detailed in Tables 3–6, 8, 9.

*Acanthospermum hispidum* (149) is recognized for its anti-HTN properties in TM in Ethiopia (55) and Brazil (150). It is also used as a diuretic in Brazil (150), Benin (151), and South America (152). *A. hispidum* exhibits both acute hypotensive and anti-HTN effects, likely through vasodilation by the activation of prostaglandin (PG) (153) and NO/cyclic guanosine monophosphate (cGMP) pathways. This action is linked to a reduction in oxidative and nitrosative stress biomarkers (150). Research by Palozi *et al*. demonstrated that *A. hispidum* is effective in inducing saluretic effects (153). The plant also possesses hepatoprotective and hypoglycemic properties, which can help manage comorbidities in hypertensive patients (149).

*Achyranthes aspera* is a widely known MP used in various traditional and modern healthcare systems, including ayurvedic, allopathic, homeopathic, naturopathic, and home remedies (154). Traditionally, it has been used to manage HTN in Ethiopia (56) and India (154, 155), as well as to promote diuresis in India (154, 156). The plant's effectiveness in reducing BP could also be linked to its confirmed antioxidant (155, 160, 161), diuretic (157–159), antiinflammatory (162), hypotensive, bradycardic (163), and antiproliferative properties. Its hepatoprotective, antiobesity, hypolipidemic, and hypoglycemic effects may further support its role in managing high BP (156).

*Ajuga remota* is an herb native to East Africa. Species of the Ajuga plant have been traditionally used as remedies for high BP in East and North Africa (61, 164). In traditional Chinese medicine, Ajuga plants are documented for their diuretic properties. One mechanism through which *A. remota* exerts its anti-HTN effects is its K-sparing diuretic action (164). Its antioxidant activity may also contribute to its role in managing HTN (165). The plant's anti-diabetic properties further enhance its suitability for treating high BP (166). It is generally accepted that plants within the same genus exhibit similar bioactivity and contain comparable bioactive compounds. In line with this, *A. bracteosa* and *A. iva*, which belong to the same genus as *A. remota*, have shown anti-hypertensive effects (167–169).

*Allium cepa*, a fiber-rich herb believed to have originated in Afghanistan, Iran, and the former Soviet Union (170), is traditionally used to treat HTN in various countries, including Ethiopia (62, 63), Morocco (14), and Palestine (171), as well as Algeria, Benin, Brazil, Congo, Italy, Kosovo, Martinique, Mexico, Nigeria, Pakistan, Peru, the Philippines, Romania, Spain, Togo, and Turkey (172). In Cuban TM, it is used as a diuretic (173). The global use of this plant highlights its ethnopharmacological significance in managing HTN.

*A. cepa* has shown diuretic effects in rats comparable to furosemide, suggesting that it functions similarly to loop diuretics (32, 173). It also exhibited hypotensive and negative chronotropic effects, with results similar to ACEIs and ramipril (174, 175). The quercetin-rich extract from *A. cepa* skin lowered arterial BP in hypertensive patients, indicating quercetin's cardioprotective properties (176). In rats, *A. cepa* demonstrated an anti-HTN effect by increasing NO levels through antioxidant activity, enhancing NOS activity (177–179), and releasing NO from S-nitroso-glutathione (180). The plant extracts may also increase BK availability indirectly through ACEI, further boosting NO levels and promoting endothelium-dependent vasodilation (181, 182). The vasorelaxation effect was also linked to the inhibition of Ca^2+^ influx in VSMCs (179). *A. cepa* bulb extract reduced vascular cell adhesion molecule 1 (VCAM-1) expression in rats, providing evidence of its anti-inflammatory properties (177). *A. cepa*'s cardioprotective, hepatoprotective, antiobesity, anti-HC, and anti-DM effects contribute to HTN management (170). However, boiling *A. cepa* is reported to diminish its anti-HTN activity (179).

*Allium sativum* is widely used around the world as both food and medicine (182, 183). It is even mentioned in the Bible and has been a traditional remedy in many countries (184). This herb is utilized in TM for its anti-HTN properties in countries such as Ethiopia (57, 64, 65), Morocco (14), Palestine (171), Afghanistan, Albania, Algeria, Benin, Brazil, Congo, Egypt, Eritrea, Germany, Greece, India, Indonesia, Iran, Iraq, Italy, Jamaica, Kosovo, Macedonia, Madagascar, Malaysia, Martinique, Mauritius, Mexico, Myanmar, Nicaragua, Nigeria, Pakistan, the Philippines, Rodrigues, Romania, Saudi Arabia, Sierra Leone, Tanzania, Thailand, Togo, Turkey, the United Kingdom, and the USA (172). This widespread use highlights its potential medicinal benefits against HTN, underscoring the need for comprehensive pharmacological research, formulation of extracts, and its application as an alternative medicine.

*A. sativum* has been found to produce hypotensive effects along with negative inotropic and chronotropic effects, which suggest a BB-like action (183, 185). The BP-lowering effect of garlic may also be mediated through the NO pathway (186). Increased urinary excretion of the stable end products of NO metabolism (nitrite/NO_2_^−^ and nitrate/NO_3_^−^) in garlic-fed rats indicates that its effect is linked to the NOS activation and NO production (187). Garlic may exhibit anti-HTN properties through anti-inflammatory effects (177, 188). The combination of captopril with fresh garlic homogenate or its bioactive component, S-allyl cysteine, has been shown to have synergistic anti-HTN and cardioprotective effects (189). Various *A. sativum* preparations have demonstrated significant anti-DM effects in patients with HTN (15, 190, 191).

The potential drawbacks of using fresh *A. sativum* (garlic) include the risk of causing indigestion and unpleasant odors, primarily due to S-allylʟ-cysteine sulfoxide (alliin). Aged garlic is believed to offer superior antioxidant benefits compared to raw garlic, particularly in reducing both physiological and psychological stress. In studies involving spontaneously hypertensive rats (SHR) and human participants, aged garlic effectively reduced high BP. The effectiveness of processed garlic products may be attributed to their ability to enhance the antioxidant status in individuals with HTN (192). In patients with treated but uncontrolled HTN, aged garlic has shown to be more effective in lowering SBP, comparable to first-line medications (193–195). S-allylcysteine, a stable and active compound in aged garlic, allows for standardized dosing. Aged garlic is also safer than other garlic preparations, as it does not pose a risk of bleeding when used with blood-thinning medications like warfarin. It is a safe and effective supplement to traditional anti-HTN Rxs for individuals with uncontrolled HTN (196).

Allicin, which is derived from alliin—the main component of fresh, raw, and powdered *A. sativum*—is unstable and easily evaporates. Consuming excessive amounts of raw garlic, which contains allicin, can lead to intolerance, gastrointestinal issues, allergic reactions, and a decrease in red blood cell (RBC) count. Cooking destroys allicin. Garlic essential oil contains diallyl disulfide and diallyl trisulfide but lacks water-soluble allicin. Standardizing and comparing products is challenging because many commercially available *A. sativum* oil preparations contain only a small amount of garlic essential oil in a vegetable oil base (197).

*A. sativum* extracts not only reduce the force of heart contractions (198) but also lower BP by inhibiting adenosine deaminase (199). In a clinical trial, garlic components like alliin, allyl disulfide, and diallyl trisulfide significantly lowered BP. When garlic was combined with vitamins C and E, BP was noticeably reduced in human participants. At the cellular level, these garlic components doubled the production of NO by endothelial cells (ECs) compared to the control. When combined with antioxidant vitamins, the production of EC NO increased nearly threefold, resulting in a vasorelaxant effect (200–202).

Low-molecular-weight thiols, such as glutathione, which react with NO, have been proposed as potential NO-carrier molecules in living organisms. Endogenous nitrosothiols, like S-nitroso-glutathione, may play a role in storing and transporting NO. Studies have shown that hydrogen sulfide (H_2_S) gas and H_2_S donors like NaHS or Na_2_S can release NO from stored nitrosothiols and biological membranes. Garlic has been found to prolong the relaxation caused by S-nitroso-glutathione and inhibit chloride channels in aortic rings contracted by norepinephrine (NE) (203). Additionally, *A. sativum* reduced endothelin 1 (ET-1)-induced vasoconstriction and showed ACEI activity, promoting vasorelaxation (181, 204, 205).

*A. sativum* induces vasorelaxation by generating H_2_S, an endogenous molecule involved in cardioprotective vascular signaling. Organic polysulfides from garlic can be converted into H_2_S by human RBCs, a process dependent on reduced thiols on the RBC membrane and supported by cytosolic glutathione levels maintained by glucose. Allyl-substituted polysulfides undergo nucleophilic substitution at the allyl substituent's α-carbon to form hydropolysulfide (RSnH), a key intermediate in H_2_S synthesis. Both RSnH and H_2_S are generated through the nucleophilic substitution of sulfur atoms in organic polysulfides. Intact aortic rings can also break down garlic-derived organic polysulfides to release H_2_S under physiologically relevant O_2_ levels. The vasorelaxation effect of garlic compounds is closely linked to H_2_S production, with stronger relaxation effects corresponding to higher H_2_S yields. These findings suggest that garlic dietary supplements can be standardized based on their H_2_S production capacity (206).

The H_2_S-dependent BP-lowering effect is thought to be primarily mediated by the sulfhydration of K^+^ _ATP_-channels, which leads to the opening of voltage-sensitive channels and the relaxation of VSMCs. The effectiveness of the H_2_S and NO signaling pathways is influenced by various dietary and genetic factors, such as deficiencies in folate, vitamin B6, and vitamin B12, as well as known genetic variations in the methylenetetrahydrofolate reductase and cystathionine-synthase genes, which may contribute to HTN. Organosulfur compounds derived from garlic could help address sulfur deficiency, one of the factors contributing to HTN (207).

*A. sativum* extract, along with the steroidal and triterpenoidal saponins in garlic, produced natriuretic and aquaretic effects in rats. It had minimal impact on K^+^ -excretion, which is a key characteristic of an effective diuretic, suggesting that garlic is more K-sparing than furosemide. K is crucial for maintaining the body's water and electrolyte balance and is vital for proper nerve and muscle function (184, 208). Studies have shown that garlic inhibits Na^+^ -transporting epithelial cells and reduces ATPase activity. Fractions of garlic targeting allicin induce diuretic and natriuretic effects in rabbits, likely by inhibiting active Na^+^ transport. This mechanism is further supported by garlic's ability to inhibit the stimulatory effects of aldosterone, Ang-II, and ADH on Na^+^ transport (209). A purified garlic fraction exhibited a biphasic diuretic and natriuretic effect, with only Cl^−^ ions, not K^+^ ions, following the natriuretic pattern. The purified garlic fraction also inhibited renal Na^+^/K^+^ -ATPase, likely by inhibiting the Na^+^ -pump at the tubular Na^+^ -reabsorption level in the kidneys (210). Garlic and its preparations also help to manage major CVD risk factors, including high serum TC, increased LDL oxidation, enhanced platelet aggregation, and impaired fibrinolysis (184).

*Calpurnea aurea* is a small tree or shrub with yellow flowers (211). The *C. aurea* subspecies *aurea* from Ethiopia is used in TM to manage HTN (61, 75–77). It has hypotensive and anti-HTN effects through relaxation of the aorta, possibly due to blood vessel dilation resulting from CCB activity (211). *C. aurea* has demonstrated antioxidant and antilipidemic effects in rats (212–214).

*Carica papaya*, a functional food crop, originated in the Mesoamerican region, including Central America and southern Mexico (215–218). All parts of the *C. papaya* plant are pharmacologically significant due to its laticifers and active compounds. The plant also contains the enzymes papain and chymopapain. The young leaves are particularly important in pharmacological research, as their components are more potent than those found in mature leaves (218, 219). Traditional healers have used *C. papaya* as a diuretic in India (217, 220) and Cuba (221) and as an anti-HTN in Ethiopia (56, 60), Nigeria (218, 220), India (217), Indonesia (222), Benin, Bougainville Island, Eritrea, French Guiana, Ghana, Guyana, Malaysia, the Marquesas Islands, Mauritius, Pakistan, the Philippines, and Suriname (172). This global ethnobotanical evidence highlights the potential of *C. papaya* in managing high BP.

*C. papaya* has been shown to produce hypotensive and anti-HTN effects in both animals and humans (215, 217). These effects may be achieved by lowering HR or by acting directly on α-adrenergic receptors in VSMCs to relax the vessel tone or reduce catecholamine release from post-ganglionic sympathetic neurons (215). In animal models of HTN, incorporating papaya leaves into the diet helped stabilize both SBP and DBP and decreased arterial stiffness (222). *C. papaya* functions as a diuretic (221) and provides chronic anti-HTN effects through ACEI. It also reversed cardiac hypertrophy (CHT) and improved arterial baroreflex sensitivity, both of which are critical for the control of mean arterial BP (MAP). HTN and the RAAS significantly contribute to the development of CHT, and studies have shown that ACEI Rx improves baroreflex sensitivity in patients with HTN, acute myocardial infarction (MI), and HF. Thus, *C. papaya*'s ability to enhance baroreflex sensitivity may be associated with its ACEI activity, leading to a reduction in MAP (223). Papaya leaves exhibited vasorelaxation, primarily through an endothelium-dependent release of NO (224).

It is well-known that DM is often associated with hyperlipidemia and HTN. When DM is not well-controlled, the risk of kidney damage increases, leading to elevated BP. Insulin plays a key role in regulating lipoprotein synthesis, and insulin resistance (IR) in DM causes the liver to overproduce lipoproteins, resulting in hyperlipidemia (222). Various *in vivo* and *in vitro* studies on *C. papaya* have demonstrated its anti-DM and antihyperlipidemic effects, which help reduce the risk of developing HTN. The plant has also shown hepatoprotective, antioxidant, antiinflammatory, antiobesity, and antiproliferative activities. These studies suggest that *C. papaya* could be used to develop pharmaceutical and nutraceutical products with potential against various diseases. However, there has been limited scientific research on the pharmacological properties of the compounds extracted from the plant (218).

*Citrullus lanatus*, native to the tropical regions of Africa near the Kalahari Desert, is consumed as food in various countries and holds global significance in managing HTN (225–228). Locally, it is used as a BP-lowering agent in Ethiopia (80), Palestine (171), Nigeria (229), and South Africa (230), and as a diuretic in Pakistan and India (228, 231). Watermelon has been shown to significantly reduce BP, likely through endothelium-dependent vasodilation due to its high L-citrulline content, which is converted to L-arginine (232). Supplementation with *C. lanatus* has also been found to reduce ankle and brachial BP as well as carotid wave reflection in patients, indicating that it improves vascular function (VF) independently of peripheral BP reduction (233). Watermelon extract exhibits therapeutic effects against HTN, DM, and CVDs (227). Consumption of *C. lanatus* has been linked to reduced BMI and body weight, improved lipid profiles, and enhanced antioxidant status, suggesting that watermelon may aid in weight management by reducing appetite and lowering CV factors (234). With its low energy density, this fruit is recommended for weight management (225).

Supplementing with NO synthesis precursors like L-arginine is essential, as vascular dysfunction (VD) often precedes CVD. However, because much of L-arginine is metabolized before reaching the endothelium, L-citrulline supplementation is recommended instead. Watermelon supplementation is beneficial in increasing plasma L-arginine levels and improving VF, as L-citrulline can be converted into L-arginine. In some cases, consuming a larger volume of watermelon (>700 ml) may be necessary to achieve an adequate dose of L-citrulline, which can be challenging. L-citrulline from watermelon appears to enhance VF by improving arterial stiffness and BP. To make regular consumption of watermelon products (juice, extract, powder, and puree) more feasible, food technologies like spray drying and freeze-drying should be used to concentrate bioactive chemicals into smaller volumes. Microencapsulation of watermelon could offer a more practical and effective method to improve adherence and vascular health in individuals with cardiometabolic risk factors (235). Beyond its potential to reduce CV risk factors (236), *C. lanatus* has demonstrated nephroprotective, antiurolithiatic, and diuretic effects in rats. Watermelon is a valuable diuretic for treating dropsy (edema) related to heart and kidney conditions due to its alkalinizing properties. *C. lanatus* exhibits hepatoprotective, antiinflammatory and anti-AS effects (228, 237, 238).

The *Citrus aurantium* tree, native to eastern Africa, has been used as an essential oil in foods (239). Traditionally, citrus plants have been credited with anti-HTN effects in countries like Ethiopia (63, 76, 78, 81–85), Morocco (14), Curacao, and India (239). *C. aurantium* has demonstrated ACEI activity (181). However, its extract and primary component, synephrine, are known to increase BP and HR (240, 241). This plant has gained popularity as a safe alternative to ephedra in herbal weight-loss products due to its potential effects on metabolism, such as increasing basal metabolic rate and promoting lipolysis, as well as acting as an appetite suppressant. Bitter orange has been included in dietary supplements for weight loss for these reasons (242). A human clinical trial showed that orange juice could raise HDL (“good” cholesterol) while lowering LDL (“bad” cholesterol). *C. aurantium* also exhibits higher antioxidant activity than many other Citrus species and has shown antiinflammatory properties. These findings suggest that p-synephrine and bitter orange extract may have beneficial effects in weight management and strenuous physical activity, as inflammation and oxidative tissue damage are linked to obesity (243). However, a recent systematic review and meta-analysis report found no evidence that p-synephrine, a protoalkaloid extracted from bitter orange, can effectively facilitate weight loss (241).

*Citrus aurantiifolia*, originating from Southeast Asia or the Indo-Malayan region, is a fruit consumed globally (244). It is also one of the most widely used medicinal herbs in African TM and plays a key role in the formulation of many herbal remedies (224, 244). This plant is commonly used to treat HTN in Ethiopia (76, 78), Ivory Coast (245), Nigeria, Pakistan (246), Benin, Congo, Cuba, Mauritius, Mexico, Rodrigues, Thailand, and Togo (172).

The anti-HTN effects of *C. aurantiifolia* are likely linked to both cardiodepressive and vasorelaxation effects through the endothelium-dependent synthesis of NO (245). This plant has demonstrated hypotensive effects in rats. It may also directly influence SMs, increasing cGMP and cyclic adenosine monophosphate (cAMP) levels by inhibiting vascular PDEs, leading to endothelium-independent vasorelaxation (246). The plant has shown ACEI activity (181). Citrus leaf extract has been found to reduce BP and vascular damage, likely due to reducing OS, which modulates vasoactive mediators and BP-regulating enzymes like ACE (237). Studies have also revealed the antiinflammatory properties of *C. aurantiifolia*. The plant's ability to induce anorexia has been associated with weight loss in mice (244). Its fruit inhibits enzymes involved in the polyol pathway, which may help mitigate complications related to DM (247).

*Citrus limon* likely originates from southern Asia (248). In countries like Ethiopia (63, 83, 84), Palestine (171), and South Africa (232), this plant is used to treat HTN. Lemon juice, widely accepted as a healthy food, is often consumed during or after exercise due to its nutritional and functional benefits (249, 250). Studies suggest that lemon juice may reduce SBP in hypertensive rats, potentially through ACEI activity (249). It also acts as an antioxidant, preventing CVD (250, 251), and has diuretic and antiproliferative properties. *C. limon* has hypocholesterolemic effects and increases HDL levels (13). Since both lemon consumption and physical activity independently lower SBP through different mechanisms, it has been suggested that combining them could be therapeutic for high BP in adults. Together, they might have additive or synergistic effects (252). Consuming lemon juice also contributes to improving patient well-being (253).

*Citrus medica* is thought to have originated in India and Asia Minor. This fragrant fruit is consumed both as a functional food and for its therapeutic benefits (254, 255). It has demonstrated anti-HTN effects in rats by enhancing biochemical and oxidative status while protecting the liver, kidneys, and vascular endothelium from damage (256). The leaves of *C. medica* have also shown significant diuretic activity in rats (257). Various studies have highlighted the plant's antioxidant, cardioprotective, antihyperglycemic, antilipidemic, anti-HC, nephroprotective (antilithiatic), and antiinflammatory properties (258–260).

*Coccinia grandis*, native to Asia, India, and Central Africa, is primarily cultivated as a food crop in various countries. Its stem, root, leaf, and fruit are valued for their medicinal properties and are used to treat a variety of ailments (261–263). In TM practices, *C. grandis* is used as an anti-HTN in Ethiopia (57) and as a diuretic in India (264). It also exhibits anti-DM, anti-HC, and ACEI activities (265, 266). In rats, *C. grandis* has shown diuretic effects comparable to those of furosemide (264). Pharmacological research has highlighted the plant's antioxidant, anti-inflammatory, anti-urolithiatic, and hepatoprotective properties (261, 262).

*Coriandrum sativum*, a well-known herb native to Western Asia and Europe (267–269), has edible parts throughout the plant, with fresh leaves and dried seeds commonly used as spices and in TM (270). In India, coriander plays a significant role in Ayurveda as an essential crude drug (268). It is used as an anti-HTN remedy by local communities in Ethiopia (57), Morocco (14), and Palestine (171), and as a diuretic in Morocco (270), India (269, 270), and Argentina (269).

Coriander has been shown to lower BP in rats through vasodilation, involving both cholinergic and CCB mechanisms. Its diuretic properties likely enhance its anti-HTN effects (267). The plant exhibits biphasic vasorelaxation: its immediate effect is due to endothelium-dependent NO release, while the delayed effect results from the inhibition of voltage-dependent CCs (VDCCs) and receptor-operated CCs (ROCCs), which prevents Ca^2+^ influx (268). *C. sativum* has the potential to inhibit ACE, making it a valuable functional food with ACEI properties (38). Consuming coriander, either alone or with garlic, has shown significant anti-DM and anti-HTN effects in patients with DM and HTN (191).

Coriander has been shown to have diuretic and saluretic effects in animals, similar to those of furosemide (270, 271). In studies, coriander extract led to a greater excretion of Na^+^ than K^+^, indicating it may be a highly effective and safe diuretic. This effect might result from increased regional blood flow, initial vasodilation, or inhibition of water and anion reabsorption in the kidneys (272, 273). *C. sativum* has been found to lower elevated levels of IR, TC, LDLC, and triglycerides (TG) while normalizing blood sugar levels. As a result, it may offer CV protection by reducing various MS components, decreasing AS, and enhancing heart-protective markers. The plant also exhibits antioxidant and liver-protective properties (268, 274).

Globally, Croton species have been traditionally used to treat HTN and edema-related conditions (275, 276). In Ethiopia, *Croton macrostachyus* is specifically employed to manage BP and urinary retention (86). Extracts of *C. macrostachyus* have shown aquaretic and aliuretic effects in rats (275). In addition, it possesses antioxidant, antiinflammatory, anti-DM, and cardioprotective properties (277, 278). Within the same genus, the essential oil of *Croton argyrophylloides* has demonstrated a vasodilator effect in aortic rings (276).

The genus Cymbopogon, which originated in India, is renowned for its high concentration of essential oils. Among its species, *C. citratus* is one of the most widely distributed plants (279). Traditionally, lemongrass has been used as an anti-HTN remedy in Ethiopia (65), Argentina, Brazil, Cuba (279), Senegal (280), Benin, Bolivia, Congo, Mexico, Nigeria, Pakistan, the Philippines, and Thailand (172). It serves as a diuretic in Brazil and Egypt (279).

Lemongrass tea has been shown to induce a hypotensive effect in humans, reducing both MAP and HR, due to its various bioactive components (45). Individuals treated with *C. citratus* experienced a significant diuretic effect, similar to loop diuretics but with a K-sparing benefit. This combined or synergistic effect of *C. citratus* phytochemicals at one or multiple target sites may help mitigate the side effects typically associated with synthetic loop diuretics (281). The anti-HTN mechanisms of *C. citratus* may be linked to its vasorelaxant, antioxidant, lipid-lowering, hepatoprotective, and nephroprotective/diuretic activities, which are attributed to its phenolic and flavonoid content (9). It has also demonstrated the diuretic and antiinflammatory properties in rats (282, 283).

A lemongrass extract has shown vasorelaxation effects in *ex vivo* studies. This activity seems to involve several biochemical mediators, including NO, prostanoids, and endothelium-derived hyperpolarizing factors (EDHFs) (224). The relaxation effect on vascular SMs, independent of the endothelium, is likely due to alterations in intracellular Ca^2+^ levels. The effect may be mediated by a PG, as the leaf infusion demonstrates cyclooxygenase (COX)-mediated vasorelaxant effects in the human thoracic artery (284). Research by Nambiar *et al*. (285) highlighted lemongrass's antioxidant and antiinflammatory properties, which help prevent blood vessel damage by increasing NO levels, aiding vasodilation. Lemongrass also exhibits hypoglycemic, hepatoprotective (286), anti-proliferative (287), hypolipidemic, renoprotective, and cardioprotective activities (288).

*Foeniculum vulgare* is a plant known for its aromatic scent and sweet seeds, commonly used as flavoring agents (289). Originally native to southern Europe and the Mediterranean (290), it is utilized in TM for managing HTN in Ethiopia (57, 60, 76), Morocco (291), and Palestine (171), and serves as a diuretic in France (292). Research shows that it lowers BP and has diuretic effects in animals (271, 273, 291, 292). It has been found to exhibit ACEI activity (181). *Marrubium vulgare*, a species related to *F. vulgare*, has demonstrated vascular relaxant effects on rat aorta (291). Numerous studies confirm *F. vulgare* as an antioxidant, antiinflammatory, antispasmodic, hypoglycemic, hypolipidemic, antianxiety, and hepatoprotective agent (290, 293).

*Hibiscus sabdariffa* is native to India and Malaysia, with its calyxes commonly used in both culinary applications and TM (294–296). Known as roselle, it is utilized in TM to treat HTN in Ethiopia (56, 91), Morocco (14), Nigeria (297–299), Senegal (299), Egypt (294, 300), Mexico (301, 302), Palestine (171), Jordan (300, 303), and Trinidad and Tobago (300). It serves as a diuretic in Mexico (301), India (296), and Germany (304). This widespread usage across various countries suggests that extracts or compounds from *H. sabdariffa* have the potential to become future anti-HTN medications.

A clinical study found that *H. sabdariffa* (sour tea) is effective in treating both uncontrolled HTN (303, 305) and essential HTN (306). It was shown to be as effective as captopril (307). Drinking sour tea significantly lowered BP in patients with stage 1 HTN (308). A systematic review and meta-analysis of randomized clinical trials (RCTs) revealed that sour tea consumption significantly reduces fasting plasma glucose levels (by 3.67 mg/dl), SBP (by 4.71 mmHg), and DBP (by 4.08 mmHg). It showed a significant reduction in LDLC levels. Therefore, drinking sour tea may aid in controlling BP and glycemic levels in adults (309).

The anti-HTN effect of *H. sabdariffa* can be driven by its antioxidant and negative chronotropic effects (298). It also induces vasorelaxation through both endothelium-dependent (activation of the NO/cGMP pathway) and independent mechanisms (inhibition of Ca^2+^ influx into VSMCs, likely through the blocking of voltage-gated CCs (VGCCs)) (310). Sour tea, either alone or combined with captopril, significantly reduced BP, ACE activity, and plasma Ang-II levels in rats. While it could be used as a supplement with captopril, it may not offer additional benefits (311). The calyxes of *H. sabdariffa* significantly lowered plasma aldosterone levels and inhibited renin activity. They also reduced iNOS, increased eNOS levels in the heart and aorta, and raised NO levels in plasma. The plant extracts demonstrated cardioprotective effects through their antiinflammatory properties (294). In hypertensive patients, Rx with *H. sabdariffa* standardized for anthocyanins lowered both SBP and DBP and also reduced serum Na^+^ concentrations without affecting K^+^ levels (312).

Consistent with its effects on laboratory animals (311, 312), *H. sabdariffa* extract has been shown to lower BP and exhibit diuretic activity in humans. The findings suggest no significant difference in effectiveness and tolerability between *H. sabdariffa* and captopril, implying that the plant may contain ACEI compounds. The diuretic effect resembles that of spironolactone-type (302). *H. sabdariffa* reduced serum ACE and plasma aldosterone levels with similar effectiveness to lisinopril in hypertensive Nigerians. The stronger effect on aldosterone compared to ACE may be due to the blocking of AT_1_-receptors and the inhibitory action of Mg^2+^ in the extract on aldosterone secretion (313). Recent studies have confirmed the natriuretic and K-sparing effects of *H. sabdariffa* extracts in rats (271, 314, 315). These extracts significantly reduced the expression of the alpha epithelial Na^+^ -channel (αENaC) in renal epithelial cells (314). These effects are partly attributed to the modulation of aldosterone activity by anthocyanins, flavonoids (such as quercetin and rutin), and phenylpropanoids like chlorogenic acid (particularly 5-caffeoylquinic acid) present in the extract. Quercetin's effect on the vascular endothelium enhances NO release, leading to increased renal vasorelaxation and improved kidney filtration (301, 314).

Multiple studies have shown that *H. sabdariffa* exhibits anti-HC, nephroprotective, and hepatoprotective properties (315, 316). This MP also has antianxiety (295) and anti-AS effects, which are beneficial in treating HTN (312). The mechanisms behind the hypotensive and anti-HTN effects of roselle extracts include the stimulation of new blood vessel formation, reduction of myocardial mass, lowering blood viscosity through COX-inhibitory activity, and inhibition of adipocyte differentiation by modulating the phosphatidylinositol 3-kinase/protein kinase B (PI3-K/Akt) and extracellular signal-regulated kinase (ERK) pathways (300).

The availability and low cost of *Hordeum vulgare* make it an excellent candidate for developing functional foods (317–319). Barley has long been used to treat various inflammatory conditions and CVDs. It aligns well with the modern dietary concept of “three high and two low,” being high in protein, fiber, and vitamins, and low in fat and sugar. This makes it particularly beneficial for individuals with DM, HTN, obesity, and CVDs (318). The United States Food and Drug Administration (FDA) has approved labeling for barley-based foods, allowing claims that consuming these foods may reduce the risk of CHD (320). *H. vulgare* is traditionally used as an anti-HTN in Ethiopia (92) and Palestine (171) and as a diuretic in India (321).

H. vulgare seeds have shown antiurolithiatic, antioxidant, and nephroprotective effects in rats (321, 322). They have also demonstrated ACEI activity (320, 323) and antiinflammatory activities (324). Juice from *H. vulgare* grass, a nutraceutical plant, has exhibited antiobesity effects in rats (50). The seeds also displayed anti-DM activity in rats through an α-glucosidase inhibitory mechanism (325, 326). This effect is further supported by the bioactive peptides (peptide hydrolysates) in *H. vulgare*, which inhibit dipeptidyl peptidase 4 (DPP-4) (319). Numerous studies have shown that DPP-4 inhibition can reduce endothelial dysfunction, inflammation, and AS progression (327).

The most extensively studied species of the *Jatropha* plant in terms of nutrition is *Jatropha curcas* (328, 329). Various parts of *J. curcas* are utilized in TM across the globe (330). For instance, its root and latex are known for their significant antioxidant, anti-HTN, antiplatelet, and antiinflammatory properties (331). Ethnomedically, the Physic nut is used as an anti-HTN in Ethiopia (93) and Cameroon (330) and as a diuretic elsewhere (332). A protein hydrolysate and its peptide fractions from *J. curcas* have demonstrated ACEI activity (328). Another species within the same genus, *Jatropha gossypiifolia*, has shown hypotensive effects in rats by acting on adrenoceptors and/or reducing Ca^2+^ mobilization (333). The fruits of *J. curcas* have exhibited cardioprotective effects in rats (331, 334). *J. curcas* holds potential for treating chronic hypertensive kidney disease (335) and has also demonstrated anti-DM effects in rats (332).

*Leucaena leucocephala* is native to Northern Central America and Southern Mexico (336, 337). Various parts of this plant are used for medicinal purposes (337, 338). The entire plant is used to manage HTN in Ethiopia (72). Its seeds are now utilized as a dietary protein source in both human and animal diets. The protein hydrolysates from this plant have demonstrated antioxidant, ACEI, and anti-DM properties (339, 340). *L. leucocephala* has shown diuretic effects in mice (341). Extracts from the White Lead tree possess antiinflammatory activities (342, 343). The plant has also been associated with hypocholesterolemic, hypoglycemic, and BP-lowering effects (342), and its antiproliferative and hepatoprotective properties are used medicinally (338).

*Linum usitatissimum*, one of the oldest cultivated plants (344), is native to Egypt and temperate regions of Europe and Asia (345–347). Flaxseed is known as a functional food (346) and is used by traditional healers to treat HTN in Ethiopia (57) and Morocco (14). The anti-HTN benefits of *L. usitatissimum* can be achieved through dietary consumption. For instance, daily intake of milled flaxseed has been shown to lower BP in patients with peripheral artery disease. Circulating levels of α-linolenic acid are associated with both SBP and DBP, while lignan levels correlate with changes in DBP (348). Flaxseed has effectively reduced BP, TC, and BMI in hypertensive individuals (349). A systematic review and meta-analysis of RCTs indicated that flaxseed oil consumption reduced SBP by 3.86 mmHg in patients with MS and related disorders. The BP-lowering effects of flaxseed oil are thought to involve ACEI, NO generation, as well as antioxidant and antiinflammatory properties. The dietary fiber in *L. usitatissimum* oil can help manage blood lipids, reduce IR, and improve intestinal microbiota, which may contribute to lower BP and weight loss (350). Flaxseed consumption has also been shown to lower BP in hypertensive patients by affecting circulating oxylipins (351).

Flax lignan concentrate demonstrated an anti-HTN effect, with its maximum dose showing efficacy comparable to that of captopril, suggesting ACEI properties. The plant may lower BP by decreasing Ang-II levels, inhibiting ET-1 production, and stimulating NOS (352, 353). Protein hydrolysates with a higher Fischer ratio also displayed antioxidant and ACEI activities. Consequently, this versatile *L. usitatissimum* peptide mixture could be used to develop dietary products beneficial for addressing OS and HTN (354). The seed extract lowered arterial BP and reduced both the force and rate of spontaneous contractions in guinea pig atria. It also shifted the phenylephrine (PE)-induced concentration-response curves (CRCs) to the right, similar to the effect of prazosin. These findings suggest that flaxseed may exert α_1_-adrenergic receptor antagonism and CCB-like activities (355). Animal studies have shown that dietary *L. usitatissimum* can provide anti-AS, antiinflammatory, nephroprotective, cardioprotective, and anti-DM effects, along with lowering cholesterol and trans fats, which may contribute to its BP-lowering benefits (347, 356).

*Lupinus albus*, native to the Mediterranean region, is traditionally prepared by soaking, scalding, and dehulling the seeds, which are then consumed as a snack in the Middle East (357, 358). In Ethiopia, *L. albus* is cultivated, and an alcoholic beverage called “Gebto Arekei” is used in TM to treat HTN, along with other food products (12). The plant is also traditionally used for managing HTN in Ethiopia (57, 73, 87, 94, 95) and Palestine (171). A decoction made from the root of *L. albus* is utilized as a diuretic in Unani Medicine (357).

“Gebto Arekei” is a traditional medicinal spirit made from a fermented brew that includes *L. albus* seeds. The residue from this spirit has been shown to lower HTN in guinea pigs (359). The distilled extract of *L. albus* significantly promotes vasorelaxation in aortic strips through the eNOS-NO-cGMP pathway, which involves the opening of K^+^ -channels and subsequent inhibition of VDCCs (360). Bioactive peptides derived from lupin seeds exhibit anti-HTN effects through ACEI activity. The plant also demonstrated antioxidant, antiinflammatory, and antiproliferative properties (358, 361). Due to their high content of non-starch carbohydrates, which are slowly digested, *L. albus* seeds have low glycemic indexes and release glucose gradually into the bloodstream, potentially helping to prevent IR-related disorders (357). *L. albus* seeds have shown significant hypoglycemic effects in rabbits, increasing insulin levels and reducing IR. Over a 12-week period, *L. albus* significantly decreased glycated hemoglobin (HbA1c) and plasma TC (362–365). White lupin has also been noted for its anti-AS activity (357).

*Melia azedarach*, commonly found in Pakistan, India, Southern China, Southeast Asia, and Australia, has long been used in TM to treat various ailments (366, 367). In Ethiopia (96) and Indonesia (368), chinaberry is used as a remedy for HTN, while in India, it is employed for treating heart disorders and as a diuretic (369). It is also a key component in Ayurvedic and Unani medicine for managing DM and HTN (370). Studies have shown that extracts from *M. azedarach* exhibit anti-HTN effects in rats (42) through reducing HR and contraction strength, relaxing blood vessels through the NO pathway, and CCB (370). Its leaves demonstrated ACEI activity (368). The plant has been found to offer nephroprotective and diuretic benefits in rats (371), along with antioxidant properties (366, 369). Further research has confirmed its antiinflammatory and cardioprotective effects (42).

*Mentha×piperita*, native to Asia and Europe (372), is commonly used as a flavoring agent (373, 374). In TM, it is used in Ethiopia to treat HTN (61, 75, 97) and as a diuretic and litholytic elsewhere (375). Studies have shown that *M. piperita* can lower BP in hypertensive patients (376). Peppermint has been found to reduce glycemia, TC, TGs, and LDL while increasing HDL levels in humans (377). Its anti-HTN effects are mainly due to its antioxidant, ACEI, and diuretic properties, along with anti-DM, anti-hyperlipidemic, anti-AS, and anti-inflammatory activities (373, 374). Peppermint extracts have also demonstrated nephroprotective effects in animal studies (378). Another species in the same genus, *Mentha longifolia*, has shown hypotensive, anti-HR, and HR-lowering effects in rats (379). Peppermint oil's ability to relax SMs suggests it may promote vasodilation, possibly through mechanisms involving PGs and NOS (372). *M. piperita* may play a vital role in preventing AS and other cardiopulmonary diseases (380). Mentha species have been shown to possess hepatoprotective properties (381).

Several species of Mentha are used in TM both as flavoring agents and herbal remedies (382, 383). *Mentha spicata* is traditionally used to manage HTN in Ethiopia (65), Palestine (171), and Morocco (14). In Morocco, it is utilized as a diuretic in TM (384). Similar to peppermint, spearmint has shown antioxidant and ACEI activity (373). In rats, it exhibits diuretic effects through a CAI mechanism (29). The essential oil has demonstrated antiproliferative properties (382). *M. spicata* also offers potential antiinflammatory and anti-DM benefits (384).

*Moringa oleifera* is primarily found in the sub-Himalayan region, though it is now widely used in food, nutraceuticals, and medicine, earning the nickname “miracle tree” (385–387). Tribal healers in Ethiopia (63), Senegal (280), Bangladesh, Benin, Eritrea, Ghana, India, and many other countries (172) use Moringa to manage HTN. In TM systems in America, it is used to treat systemic arterial HTN (388). In Pakistan, the roots of Moringa are utilized in TM as a diuretic and to treat kidney stones (389).

Moringa leaves have been found to effectively lower BP in both hypertensive patients and healthy individuals (390–392). Many believe it is more effective than modern medicine, as Moringa leaf provides a more consistent reduction in BP over a short period, unlike some modern Rxs. As a result, *M. oleifera* leaves can be considered an alternative therapy for HTN. Modern pharmaceuticals isolate specific phytochemicals to produce medicines, but consuming the whole Moringa leaf allows the various phytochemicals to work together, enhancing their overall effect (385). Moringa leaves not only lower BP in normal and obese hypertensive individuals but also promote weight loss, offering an added benefit for managing HTN in obese patients (393).

In animal studies, *M. oleifera* also demonstrated hypotensive and anti-HTN effects. Extracts, including those containing gamma-aminobutyric acid (GABA), led to BP reduction in rats (394, 395). These effects are partly attributed to an endothelium-dependent vasodilator effect, primarily through activation of the eNOS-NO-soluble guanylyl cyclase (sGC) pathway, along with reductions in HR and CCB activity. Moringa's antioxidant properties further reduce OS and VD (11, 396–398). Besides, Moringa extracts may work by inhibiting adrenergic receptors (399).

In another study, a peptide fraction smaller than 1 kDa, derived from *M. oleifera* leaves, showed significant BP-lowering effects. This was attributed to the small peptide size and the presence of aromatic and hydrophobic amino acids. Two specific peptides, with the sequences Leu-Gly-Phe-Phe (LGF) and Gly-Leu-Phe-Phe (GLFF), significantly reduced BP in rats and inhibited both renin and ACE activity. It was confirmed that LGF and GLFF are resistant to gastrointestinal digestion, maintaining their structure. These findings suggest that Moringa-derived peptides could serve as potential therapeutic agents for managing HTN (8).

Moringa extracts also act as ACEIs (400–402) and have shown significant diuretic effects (402–405). The saluretic index indicates that these extracts may function as loop diuretics or CAIs (405). Pharmacological research has confirmed that Moringa extracts possess a range of beneficial activities, including antispasmodic, anti-DL, antihyperglycemic, cardioprotective, hepatoprotective, antiproliferative, and antiinflammatory effects (11, 387, 401, 403). As a result, *M. oleifera* extracts hold promise for development into effective anti-HTN products.

*Moringa stenopetala* is native to northeastern tropical Africa and is a common vegetable in southwestern Ethiopia. It has a variety of traditional uses, including as food and in medicinal applications (406, 407). In Ethiopia, it is widely used to treat HTN, and a standardized herbal product made from Moringa is available on the local market (57, 69, 72, 75, 76, 80, 99–101). Studies on *M. stenopetala* have shown it to have hypotensive and anti-HTN effects in animals. Its leaf extract induces vasorelaxation by blocking voltage-sensitive CCs (VSCCs) and exhibits antispasmodic effects (18, 408). Both microencapsulated bioactive products and leaf extracts demonstrated anti-DM, vasodilatory, and diuretic effects in rats (409, 410). The diuretic effect was comparable to that of furosemide, with significant natriuretic and kaliuretic effects. These findings support its traditional use for managing HTN (406). Pharmacological studies further highlight the plant's antioxidant, antiinflammatory, and anti-DL properties (18, 407).

*Nigella sativa*, native to South and Southwest Asia, has been used for centuries in Arab countries, the Indian subcontinent, and Europe for both culinary and medicinal purposes (411–413). In TM, it is used to manage HTN in Ethiopia (57), Morocco (14), Algeria, and Egypt, and as a diuretic in China (411) and Pakistan (45). In Islamic tradition, the “black seed,” as it is called in Arabic, is considered a universal healer, able to treat all ailments except aging and death (414). Given the high prevalence of HTN alongside DL, many hypertensive patients may benefit from Rxs that also lower lipid levels. *N. sativa* seeds and their oil are recommended for managing DM and HC due to their significant antioxidant, anti-HTN, antiobesity, antihyperlipidemic, and hypoglycemic effects in humans. Studies found that its BP-lowering effect (71%) exceeded that of the positive control (57%) (415–420).

In animal studies, *N. sativa* extract and oil demonstrated endothelium-independent vasorelaxation, primarily through the blockage of ROCCs and VDCCs, activation of K^+^ _ATP_-channels, and suppression of IP_3_-mediated receptors (421, 422). This suggests that Nigella oil could be effective as an anti-HTN in humans. *N. sativa* significantly reduced cardiac contractility and HR in guinea pigs, with effects greater than those of diltiazem, indicating the extracts might act as CCBs or K^+^ -channel openers in the heart (423). Other *N. sativa* extracts induced vasorelaxation in aortic rings, likely due to increased endothelial NO production. In rats, the extract also had a hypotensive effect, possibly by inhibiting SNS activity (424). Both dethymoquinonated and regular volatile oil from black seed lowered arterial BP and HR in rats, with the CV depressant effects potentially mediated by central mechanisms involving activation of 5-hydroxytryptaminergic and muscarinic pathways (425, 426).

HTN and T2DM often occur together, and the onset of DM is frequently linked to pre-HTN and HTN. Thus, managing either DM or BP in affected individuals could reduce the likelihood of developing the other condition (426). Studies showed that crude extract and ammonium sulfate fractions of *N. sativa* are effective in inhibiting both Dpp-4 and ACE. The crude extract exhibited the highest ACEI, likely due to the presence of other water-soluble compounds. The 30% ammonium sulfate fraction had the pronounced inhibitory activity against both ACE and Dpp-4, while the 60% fraction showed the highest trypsin inhibitory activity. Trypsin inhibitors from various plants are known for their antiproliferative properties (427). The anti-HTN effects of *N. sativa* seed oil are thought to stem from reduced OS in the heart, increased ACEI, enhanced cardiac heme oxygenase 1 (HO-1) activity, and prevention of plasma NO loss. HO-1's anti-HTN effects result from its production of carbon monoxide, which acts as a vasodilator by stimulating NO release, reducing SNS activity, and promoting Na^+^ excretion. The BP-lowering effect of *N. sativa* oil was comparable to that of the standard drug nicardipine (428).

*N. sativa* has demonstrated anti-HTN effects and protection against HTN-induced tissue damage, improving CVS function in rats. This suggests that *N. sativa* may have a promising potential for treating renovascular HTN (RVH) (429, 430). It exhibited diuretic activity in rats, with Na^+^ excretion levels lower than the reference standard, indicating a reduced risk of hyponatremia (431). The K^+^ levels in the urine of the treated groups were similar to those in frusemide-treated animals, suggesting a loop diuretic-like effect (41). *N. sativa* has also shown nephroprotective, antiinflammatory, and hepatoprotective effects, and a reduction in ischemia-reperfusion injury (432, 433).

*Otostegia integrifolia*, endemic to Ethiopia, Eritrea, and Yemen, is well-known in Ethiopia for its distinct aroma and robust medicinal properties (434, 435). It has been traditionally used to treat HTN in both Ethiopia (57, 84) and Eritrea (44). Rat BP significantly decreased after administration of the leaf extract, and aortic strips also showed relaxation. Similar to nifedipine, the extract shifted the Ca^2+^ CRC to the right, suggesting that its vasorelaxant effect is likely mediated through CCB activity (44). *O. integrifolia* extracts have antioxidant, antiinflammatory, anti-DM, and oral GT-enhancing effects (435–437).

*Passiflora edulis*, native to Brazil, Paraguay, and Argentina, is often consumed fresh as fruit pulp or juice (438–440). It is used in traditional remedies in various countries (441). The plant has been employed to manage HTN in Ethiopia (60, 68), South America, and India (442) and is commonly used as a diuretic in China, South America, and India (441). In hypertensive patients, passion fruit juice has been shown to lower BP and exhibits high antioxidant activity (443). A significant reduction in SBP and fasting blood glucose levels was observed after administering a flavonoid-rich peel extract to adults with T2DM. Purple passion fruit provided further reductions in SBP when used alongside anti-HTN drugs (444).

*P. edulis* extracts significantly reduced BP and HR in hypertensive rats (445, 446). Its fruit extract, which contains bioflavonoids, phenolic acids, and anthocyanins, significantly lowered BP in SHR. The results suggest that the anti-HTN effects of the extract may be partly due to the down-regulation of iNOS expression by compounds such as quercetin, luteolin, and cyanidin 3-O-glucoside, or through the scavenging of NO radicals by quercetin with help from other flavonoids. The modulation of NO production and the scavenging of free O_2_ species by flavonoids may reduce peroxynitrite anion formation, thereby minimizing its negative impact on the body's antioxidant system. This leads to changes in vascular tone and peripheral vessel resistance, ultimately lowering BP (446). Another potential anti-HTN mechanism involves the down-regulation of ENaC expression in the kidney by quercetin, which influences fluid volume through Na^+^ reabsorption in the kidney (447).

In hypertensive rats, yellow passion fruit pulp significantly reduced SBP. Rx with this fruit pulp showed a protective effect on the kidneys (448). Along with vasodilation, the extracts were found to possess antiinflammatory, antihyperlipidemic, anti-DM, and antiglycant properties (438, 449). A clinical trial indicated that *P. edulis* fruit juice is a safe and effective co-adjuvant to enalapril for lowering BP (450). The vasorelaxant effect may primarily occur through the opening of K^+^ -channels (451). The plant also demonstrated cardioprotective effects (452). *P. edulis* extracts were found to have significant ACEI activity and exhibited diuretic effects in rats (453, 454). Similarly, *Passiflora nepalensis*, a related species, showed prominent diuretic effects in rats (455). The fruit peel extracts of *P. edulis* have proven to be a rich source of bioactive substances that could be used in the production of pharmaceutical or nutraceutical products to manage HTN.

*Persea americana*, native to Mexico and Central or South America, is often called a “superfood” due to its exceptional nutritional and phytochemical profile compared to other fruits (456–459). In West African countries (460) and Brazil (43), avocado leaves are used as a diuretic, and in Brazil and Jamaica, they are used to treat high BP (461). *P. americana* is utilized in TM across various countries, including Ethiopia (75, 102), Benin, French Guiana, Ghana, Guinea, Guyana, Indonesia, Mauritius, Mexico, Nigeria, Panama, the Philippines, South Africa, Suriname, and Togo, for managing HTN (172). This widespread use highlights the plant's potential medicinal value in addressing HTN.

*P. americana* extracts have shown hypotensive, bradycardic, and vasorelaxant effects in animal studies (43, 456, 460). The non-parallel rightward shift of the NE CRC caused by the extract suggests its action involves blocking α_1_-adrenoceptors. The vasorelaxation effect is likely due to endothelium-dependent NO production and cGMP release (460). The extract also induced significant vasorelaxation in isolated rat aorta, depending on the synthesis or release of endothelium-derived relaxing factors (EDRFs), the activation of prostanoid receptors (PGI_2_ and PGE_2_), blocking VDCCs, and, to a lesser degree, ROCCs (461). The extract also demonstrated cardioprotective effects in rats (462).

*P. americana* revealed ACEI activity comparable to captopril (463–466). Nanoparticle extracts significantly lowered BP, reduced ACE activity, and increased serum nitrite and nitrate levels in hypertensive rats. The nanoparticle method minimizes dosing frequency while optimizing efficacy in target organs due to improved pharmacokinetics (466). The extract also exhibited diuretic effects (465–467), increasing Na^+^ excretion more than K^+^, a desirable trait for diuretics to reduce the risk of hyperkalemia. Cl^−^ excretion was also significantly elevated, indicating a natriuretic effect (37). Overall, avocado has been shown to possess antioxidant, lipid-lowering, anti-DM, antiobesity, antithrombotic, anti-AS, and antiinflammatory properties in various studies (457, 458, 463, 468, 469).

*Rosmarinus officinalis*, native to the Mediterranean region, is used as a culinary spice, a natural food preservative, an ornamental plant, and for its medicinal properties (470). Traditionally, rosemary is employed to manage HTN in Ethiopia (94, 101), Morocco (14), and Palestine (171). The plant showed anti-HTN effects through vasorelaxant activity, likely due to increased NO production and reduced ang-II levels (471). Rosemary leaf extract displayed diuretic effects in rats (472). Its extract and volatile oil exhibited spasmolytic activity, potentially through muscarinic receptor and CC blockade (473, 474). Additional benefits of rosemary include antioxidant, antiinflammatory, anti-AS, anti-HC, anti-DM, antiproliferative, and glycemia-lowering effects (470, 475).

*Rumex abyssinicus*, native to Ethiopia, has edible tender shoots and leaves (46, 476, 477). In Ethiopian TM, it is used to treat HTN (57, 73, 75, 77, 81, 83, 84, 89, 92, 104), while in Pakistan's ethnomedicine, its leaves are used as diuretics (478). The rhizomes of *R. abyssinicus* have demonstrated diuretic effects in mice, with activity similar to furosemide, suggesting that the active compounds may act in a similar way. The rapid onset of diuretic action indicates quick absorption from the intestine, and the ethanolic fraction has a long-lasting effect, particularly at higher doses, which is beneficial for reducing the frequency of administration of loop diuretics (46, 476). A related species, *Rumex acetosa*, has shown anti-HTN effects in rats through vasodilation (479). Extracts and compounds from *R. abyssinicus* also exhibit antioxidant, antiinflammatory, hepatoprotective, and anti-DM properties (478, 480).

*Ruta chalepensis*, native to Mediterranean Europe and Western Asia, is cultivated for ornamental purposes, as a food flavoring, and primarily for medicinal uses (481, 482). In Mexican (481) and Ethiopian (57, 73) folk medicine, rue has been used as a Rx for HTN. *R. chalepensis* has shown a significant hypotensive effect in rats, potentially through α-adrenergic mechanisms (481). The plant also demonstrated noteworthy antioxidant and vasorelaxant activities by reducing OS and inflammation, modulating the RAAS, or improving ECs and VF through its phytochemicals (10). *R. chalepensis* infusion in rats caused an endothelium-dependent increase in the release of COX-dependent vasoconstrictor prostanoids and basal NO release. It is likely that the rise in NO acts as a compensatory mechanism for the increased release of prostanoids (482). The BP-lowering effect of *Ruta montana*, a related species within the same genus, further supports the anti-HTN activity of *R. chalepensis (*483). *R. chalepensis* has demonstrated antispasmodic and platelet aggregation inhibition activities (484, 485).

The leaves and flowers of Satureja species are used to produce essential oils, commonly utilized for food flavoring and medicinal purposes (486–488). In Ethiopia, *Satureja punctata* is used to manage HTN (56, 93). It has been shown to relax the aorta and reduce BP in rats, likely through blockage of Ca^2+^ influx. Additionally, the plant extracts have demonstrated anti-DM, antioxidant, and hepatoprotective properties (489–491).

*Schinus molle*, a tree native to the subtropical regions of South America, produces red, edible fruit with a high aromatic and chemical content (492–496). In TM, both in Ethiopia (84) and South America (497), *S. molle* is used to treat HTN. It is commonly used in Peruvian folk medicine as a hypotensive agent (498) and serves as a diuretic in various TM systems (492, 495, 499, 500). Extracts of *S. molle* have been shown to significantly lower MAP in normal rats. At a 100 µg/ml dose, the extract reduced the contractile response to noradrenaline (NA) in the rat vas deferens, suggesting that it acts as NA receptor antagonist (498). *S. molle* leaf extracts have demonstrated ACEI activity (501) as well as antiinflammatory and anti-spasmodic effects (494, 495). The essential oils and various extracts also exhibit promising antioxidant properties (502, 503).

*Solanum nigrum* is a lesser-known food crop in many developing countries (504, 505). Both its berries and leaves are edible, though the leaves contain high levels of alkaloids that require cooking to remove their toxicity (504). In Ethiopia, the plant is traditionally used to manage HTN (85), and it serves as a diuretic in both Libya and traditional Chinese medicine (504). Studies on normal and diabetic rats showed that the fruit of *S. nigrum* causes vasodilation. In diabetics, this vasorelaxation is mediated by both the endothelium and SM, while in non-diabetics, it occurs through direct action on the SM, independent of the endothelium (506). *S. nigrum* has been reported to have hypotensive (504, 507), ACEI (464), and cardioprotective properties (508). The leaf extracts exhibit diuretic effects similar to furosemide, but with the added benefit of maintaining K^+^ levels (509). Several Solanum species are known for their anti-HTN and diuretic properties (510–513). The plant also exhibits anti-DM, antioxidant, antihyperlipidemic, antiinflammatory, hepatoprotective, and antiproliferative effects, all of which may contribute to its anti-HTN activity (504, 514–517).

*Syzygium guineense* is a fragrant plant native to the wooded savannahs and tropical forests of Africa, known for its edible fruits (518, 519). In Ethiopian TM, it is used to manage HTN (105, 518). *S. guineense* has been shown to lower BP in rats and induce vasorelaxation in the aorta (518). An extract from the bark has demonstrated sustained hypotensive and antispasmodic effects (520). Related species like *S. samarangense* and *S. cumini* have shown anti-HTN properties (521–523). Scientific evidence supports *S. guineense*'s effectiveness against DM and inflammation (524), and its extracts have exhibited organo-protective and antioxidant activities (525).

*Tamarindus indica* is a widely used ingredient in Indian cuisine, originating from tropical Africa and found in Central America and Asia (526–528). In TM, tamarind has been utilized for treating HTN in Ethiopia (106), Palestine (171), and Senegal (279). Its diuretic properties have been documented (529), with tamarind fruit pulps outstandingly lowering DBP, TC, and LDLC in humans (530). Tamarind has demonstrated considerable diuretic effects in rats, leading to a significant increase in the excretion of K^+^, Cl^−^, and Mg^2+^ ions, as well as a marked rise in urinary oxalate excretion (271, 529).

Extracts from both sour and sweet tamarind have been shown to have anti-HTN effects in rats. Further advanced preclinical and clinical studies may establish ripened sour tamarind extract as a more effective anti-HTN agent or nutraceutical (531). Along with its diuretic properties, *T. indica* may induce vasodilation, as its seed coat flavonoids are found in Pycnogenol®, a supplement known for its vasorelaxant effects (532). The plant's effect might also be due to its antioxidant, antiinflammatory, antiobesity, antilipidemic, anti-HC, cardioprotective, antiproliferative, anti-DM, and hepatoprotective properties (526, 527, 532–535).

*Thymus schimperi* is a plant endemic to Ethiopia (23), where it is used both in TM and as a food flavoring (536). It is commonly used by Ethiopian patients to manage HTN (57, 59, 65, 69, 75, 87, 92, 99, 101, 104, 107–109). *T. schimperi* has shown a relaxing effect on thoracic aortas by blocking ROCCs and VDCCs and activating K^+^ _ATP_-channels, H_1_, and M_3_-receptors (23). Both the leaves and essential oil of the plant possess anti-HTN and diuretic properties, likely due to the high K or phenolic content. The K may promote endothelium-dependent vasodilation (54). *T. schimperi* has demonstrated antioxidant and anti-DM activity (537, 538).

The Ethiopian endemic plant *Thymus serrulatus* is traditionally used both as a food ingredient and as a remedy for various health conditions. In Ethiopian TM, its decoction is used to manage high BP and DM (109, 110). This species is frequently added to tea, coffee, and various stews for flavor (539). *T. serrulatus* has been found to have a vasodilatory effect that depends on the endothelium linked to the activation of the cGMP-NO pathway (540). Thyme species are commonly recognized for their diuretic properties worldwide, and *T. serrulatus* has shown significant diuretic effects (30). Extracts and essential oil from its aerial parts exhibit antihyperglycemic properties, with phenolic compounds enhancing blood glucose-lowering effects (539). Its essential oil also demonstrated nephroprotective properties due to its antioxidant and antiinflammatory actions (541).

*Trigonella foenum-graecum* is native to regions spanning from the Eastern Mediterranean to Central Asia and Ethiopia. It is commonly used as a spice in food and as an ingredient in TM. In the Unani medical system, it is employed as a diuretic (542). In Ethiopia (57, 75) and Morocco (14), *T. foenum-graecum* is traditionally used to manage HTN. Studies have shown that extracts from it have significant anti-HTN and anti-HC effects (5). The extract may lower BP by inhibiting the overexpressed 5-HT_2B_ receptors (543). In diabetic rats, fenugreek extract has been found to enhance the relaxation response to Ach and partially reduce the heightened contractile response of endothelium-intact aortic rings to NE and/or KCl. These effects are thought to be partly mediated by the PG synthesis pathway (544). Fenugreek seed flour promotes endothelium-dependent vasorelaxation at lower levels of Ach compared to diosgenin, indicating that other components of fenugreek may contribute to its beneficial effects. The plant has shown antioxidant and ACEI activities (181, 545).

Gelatinous capsules containing fenugreek extract showed only mild diuretic effects in patients with cirrhotic ascites (546). However, fenugreek extracts demonstrated effective diuretic and natriuretic activity in rats. The increased excretion of Na^+^ and K^+^ by the water extract is an important feature of a good diuretic, as it reduces the risk of hyperkalemia (547). *T. foenum-graecum* also possesses antiinflammatory, anti-DM, hepatoprotective, and nephroprotective activities (542).

*Vernonia amygdalina*, native to tropical Africa, is commonly known as “bitter leaf” due to its distinct bitter flavor, attributed to its anti-nutritional components such as alkaloids, saponins, tannins, and glycosides. However, the bitterness can be reduced by boiling or soaking the leaves in water. The leaves are widely used in various African dishes (548–50). Traditionally, the plant is used to treat HTN in Ethiopia (57), Togo (551), Nigeria (229), and Uganda (552). In Malaysia, it is known for treating DM and HTN (553). Leaf extracts of *V. amygdalina* have been shown to reduce heart contractility force and rate in isolated rabbit hearts, similar to the effects of Ach, indicating the presence of active compounds and minerals (552). The plant also significantly lowers BP, HR, and blood volume while enhancing antioxidant activity (554, 555).

*V. amygdalina* leaves affected BP in rats in a biphasic manner, suggesting the presence of multiple compounds in the extract that influence BP. When the extract was added to the pre-contracted aortic rings in rats, it induced SM relaxation in the aorta (556–558). The primary pathway responsible for the plant's vasorelaxant effects involves EDRFs like PGI_2_ and the NO/cGMP pathway. This is followed by opening K^+^ -channels and activating endothelium-independent relaxing factors through M_3_- and β_2_-receptors. The extract also reduced vasoconstriction by inhibiting the intracellular release of Ca^2+^ through the IP_3_-receptor. Fourier-transform infrared (FTIR) spectroscopy identified alkaloids, flavonoids, and saponins in the extract (558). *V. amygdalina* exhibits diuretic effects in rats, likely due to its impact on renal Na^+^ handling and interaction with the adenosine A_1_-receptor (47). Various studies have highlighted its bioactive components and their antiinflammatory, anti-DM, antiobesity, and hepatoprotective properties (550).

*Zingiber officinale*, native to southern China, is widely used as a spice and flavoring agent in cuisines around the world. The essential oils extracted from ginger rhizomes, containing both aromatic and pungent compounds, are used to preserve food by preventing autoxidation and microbial spoilage (559–561). In TM, ginger is used to treat HTN in Ethiopia (57, 84), Indonesia (562), Palestine (171), Senegal (280), and Nigeria (229). In Pakistan, herbalists recommend HTN patients consume ginger after dinner (563). According to Ayurvedic principles, ginger helps clear blockages in blood vessels (564).

Healthy individuals who consumed *Z. officinale* rhizome experienced a significant decrease in BP and HR. It was suggested that ginger improves blood flow to the peripheral blood vessels, indicating a reduction in vascular resistance. As a result, *Z. officinale* lowers PVR and consequently reduces arterial BP (560, 565). A pooled analysis of clinical trials found that ginger supplements could reduce SBP by 6.36 mmHg and DBP by 2.12 mmHg (566). Another systematic review and meta-analysis showed significant reductions in fasting blood sugar (FBS), HbA1C, SBP, and DBP in patients with T2DM after ginger supplementation (567).

The rhizome extract of *Z. officinale* and its fractions significantly lowered BP in hypertensive rats. Rx with the extract shifted the cumulative CRC of serotonin (5-HT) to the right in the rat fundus, indicating antagonism of the 5-HT_2B_ receptor (568). Ginger rhizome also showed hypotensive effects in rats, possibly through negative inotropic and chronotropic effects and endothelium-independent vasodilation by blocking VDCCs (563, 569). The anti-HTN effects of ginger rhizome extracts in rats involve mechanisms such as NO and PGI_2_ release, activation of cGMP‒K^+^ _ATP_-channels, stimulation of muscarinic receptors, and Ca^2+^-release from intracellular stores via transmembrane CCs (570, 571). Ginger helps lower SBP and serum soluble intercellular adhesion molecule 1 (ICAM-1) levels (562). *Z. officinale* extract also induced endothelium-dependent vasorelaxation and enhanced vascular protection against EC damage using its antioxidant properties. Overall, the plant extract exerts significant vasodilatory, vasoprotective, and free radical-scavenging effects (2).

White and red ginger demonstrated notable ACEI activity in rats and also showed anti-HC effects due to this ACEI activity. Ang-II disrupts the binding of LDLC to its receptors and increases endothelial uptake of LDL. Using ACEIs can counteract these negative effects and may reduce the risk of MI associated with high plasma levels of ang-II. Thus, both types of ginger could be used as nutraceuticals for treating HTN and other CVDs (34, 181, 572). Moreover, ginger demonstrated diuretic effects comparable to furosemide (573) and potentially as effective as K-sparing diuretics (574). Accumulating evidence supports various pharmacological effects of ginger and its derivatives, including antiinflammatory, antiobesity, anti-DM, antiproliferative, antithrombotic, cardioprotective, and hepatoprotective properties (561, 575, 576).

In summary, the importance of traditional medical practices and indigenous knowledge in treating HTN is underscored by the experimentally confirmed anti-HTN properties of MPs from various regions. The pharmacological effects of these anti-HTN plants are closely aligned with traditional herbal formulations. Before advancing to clinical trials, further studies on the mechanism of action and anti-HTN activity of extracts and secondary metabolites from plants such as *A. hispidum*, *A. aspera*, *A. remota*, *C. aurea*, *C. aurantium*, *C. aurantiifolia*, *C. limon*, *C. medica*, *C. grandis*, *C. citratus*, *F. vulgare*, *H. vulgare*, *J. curcas*, *L. leucocephala*, *L. albus*, *M. azedarach*, *M. spicata*, *O. integrifolia*, *R. officinalis*, *R. chalepensis*, *S. punctata*, *S. molle*, *S. nigrum*, *S. guineense*, *T. schimperi*, *T. serrulatus*, and *T. foenum-graecum* are recommended. Additional preclinical studies focusing on the mechanism of action, pharmacokinetics, and anti-HTN effects of *C. lantus*, *C. limon*, *M. piperita*, and *T. indica* are also advised. While *in vitro* and *ex vivo* or *in vivo* studies have been conducted on extracts of *C. sativum*, *L. usitatissimum*, *P. edulis*, and *Z. officinale*, more clinical research is needed. Similarly, although preclinical investigations have been carried out on *A. cepa*, *M. stenopetala*, *P. americana*, and *V. amygdalina*, clinical studies have only been performed on *A. cepa*. Extensive animal and human studies have been conducted on medicinal preparations of *A. sativum*, *H. sabdariffa*, *M. oleifera*, and *N. sativa*. Consequently, *A. cepa*, *A. sativum*, *H. sabdariffa*, *M. oleifera*, and *N. sativa* are promising candidates for developing standardized natural products, nutraceuticals, or anti-HTN agents.

### 5.3 Phytochemistry of medicinal plants with antihypertensive activity

Since ancient times, MPs have been used as a source of Rx. Globally, around 35,000–70,000 plant species are employed for medicinal purposes. The modern pharmaceutical industry is increasingly looking to traditionally used MPs to counteract the effects of synthetic drugs at the cellular level. These plants are rich in phytochemicals, which have played a key role in the development of many modern medicines (577). Phytochemicals consist of both primary and secondary metabolites (212). The secondary metabolites, such as alkaloids, flavonoids, glycosides, phenols, phlobatannins, tannins, terpenoids, and volatile oils, enhance the therapeutic purpose of plants for treating a wide range of diseases (577). Flavonoids, a large group of polyphenolic compounds found in commonly consumed foods like fruits and vegetables, are divided into subgroups based on their chemical structure: flavones, flavanones, flavanols, anthocyanidins, and chalcones (48). Polyphenolic compounds include anthocyanins, flavonoids, phenolic acids, and phenolic diterpenes (531). Various phenolic compounds present in plants include benzoquinones, polypropenol, isoflavonoids, flavonol derivatives (such as carotenoids and β-cryptoxanthin epoxide), phenylpropanoids, phenolic quinones, lignins, melanins, tannins, and more (149). Research has shown that polyphenols offer several biological and therapeutic benefits, including antioxidant, anti-AS, and antiinflammatory properties, useful in treating human diseases (323). Natural polyphenols, widely used in the functional food industry, have been shown to protect against CVD, DM, and HTN by inhibiting key enzymes like ACE, α-glucosidase, and α-amylase, increasing NO levels, and improving EF (373).

The secondary metabolites found in the ethanol-soluble fractions of *A. hispidum* with hypotensive and anti-HTN effects include phenolic compounds like caffeoylquinic acids, dicaffeoylquinic acids, and glycosylated flavonoids (quercetin glucoside and galactoside). Derivatives of caffeoylquinic and dicaffeoylquinic acids, such as chlorogenic acid (Figure 5), are well-absorbed in the intestines and can be easily detected in human plasma. Numerous clinical and preclinical studies have shown that chlorogenic acid (1) is an effective hypotensive and anti-HTN agent. It helps reduce OS and increases NO bioavailability, which improves EF and lowers overall PVR (137). Mannitol, extracted from the aerial parts of *A. hispidum* (156), may contribute to the plant's saluretic activity.

Saponins extracted from *A. aspera* showed diuretic effects in rats (578). These compounds need structural elucidation to identify the active components. Another active compound responsible for the plant's diuretic and antiinflammatory effects is achyranthine, which is included in the polyherbal diuretic formulation Cystone® (159, 160). Achyranthine (2), a water-soluble alkaloid, was also found to lower BP and HR, as well as dilate blood vessels, in animal studies (579). The pharmacological activity of *A. aspera* is attributed to its diverse phytochemical constituents, particularly oleanolic acid, achyranthine, and ecdysterone (577). The key compounds in the plant, including flavonoids, tannins, terpenoids, and alkaloids, may have contributed to the diuretic effects observed in *Ajuga remota* (164).

The hypotensive effects of *A. cepa* (onion) peel extract may be attributed to its quercetin content (179). Most of the quercetin in onions is in a glucoside form, which is highly absorbable. *A. cepa* exhibits antioxidant properties due to the presence of organosulfur compounds (such as S-propenylcysteine sulfoxide, the major component, along with S-propylcysteine sulfoxide and S-methylcysteine sulfoxide), polyphenols, and flavonoids. In rats, dietary flavonoids like quercetin and catechin were shown to reduce lipid peroxidation. Quercetin (3), a polyphenol, is responsible for the anti-HTN and vasorelaxant effects of onions (175, 178). Studies have shown that rutin and quercetin lowered BP in animals (174). Quercetin also reduced high BP, cardiac and renal hypertrophy, and vascular changes in SHR rats (580). Additionally, onion peel, rich in quercetin and FRS 1,000, has been found to strongly inhibit PDE 5A activity (179).

*A. cepa* may exert its hypotensive effect by inhibiting ACE through one or more of its flavonoid constituents, such as quercetin (174). Quercetin could lower BP by improving vascular EF. This hypothesis is supported by several findings: (a) in normotensive humans, quercetin increased plasma quercetin and NO metabolite levels while reducing ET-1; (b) in hypertensive rats, quercetin reduced VD in a NO-dependent manner; and (c) *in vitro* studies showed that quercetin decreased cellular production of ET-1 and endothelial adhesion molecules (175). *A. cepa* contains metabolites like potassic salts, alkaloids, cardiac glycosides, steroids, and triterpenoidal saponins, which may contribute to its diuretic effects (32, 173). Anthocyanins and S-methyl cysteine sulfoxide from onions help lower serum cholesterol, TGs, and phospholipids in rats (174). Dietary nitrate and nitrite from onions may also have physiological effects, contributing to increased urinary nitrate and nitrite, while dietary arginine from onions may boost NO levels (178).

Components of *A. sativum* (garlic), including S-allyl-L-cysteine (4), alliin (5), allyl disulfide (6), and diallyl trisulfide (7), have been shown to lower BP in both rats and hypertensive patients (184, 192, 200). Allicin (8) reduced BP and TG levels in SHR rats (581). This compound can induce vasorelaxation in rat arteries, likely through garlic's ability to activate NO formation (201). S-allyl cysteine from garlic enhances eNOS activity and increases cGMP levels (582), while diallyl disulfide and diallyl trisulfide promote eNOS activity, protect against eNOS degradation, and elevate both NO and cGMP levels. Diallyl trisulfide also boosts H_2_S levels (583, 584). Allicin in garlic inhibits Ang-II activity (191), and various organosulfur compounds from garlic disrupt cholesterol biosynthesis (210). Aged garlic extract has demonstrated radical-scavenging properties due to the presence of S-allyl-L-cysteine and S-allylmercapto-L-cysteine (201). S-allyl cysteine triggers an antioxidant response by activating the nuclear factor-erythroid 2 related factor 2 (Nrf2) signaling pathways (585). N-acetylcysteine, a water-soluble chemical, reduces LDL oxidation and OS, and improves GT and lipid profiles in rats (51).

The ACEI activity of *A. sativum* is attributed to the presence of γ-glutamyl-allyl-cysteine sulfoxide and other glutamyl peptides in garlic (205). Compounds like S-allyl cysteine sulfoxide and methiin have displayed anti-DM properties (190). Alliin possesses anti-DM, anti-HC, and antiinflammatory effects (586). Nonsulfur compounds such as saponins may also contribute to garlic's key biological activities. Quercetin significantly reduced the rise in BP in rats. This compound's effect is likely due to its ability to enhance NO availability by boosting NOS activity and exhibiting antioxidant properties (587).

*A. sativum* contains several active components that contribute to its hypotensive properties, including adenosine, arginine, ascorbic acid, Ca, Mg, K, quercetin, tryptophan, and tyrosinase. The increase in GFR caused by *A. sativum* extract may be due to several factors: (a) triterpenoidal saponins interacting with glomerular membrane components, influencing fluid filtration; (b) reduced renal perfusion pressure, likely from decreased resistance in the afferent arteriole or increased resistance in the efferent arteriole; (c) a direct effect on arterial pressure, as garlic is known to relax vascular SMs, causing vasodilation—this may be due to the breakdown of garlic-derived polysulfides into H₂S in RBCs, a reaction dependent on reduced thiols in or on the RBC membrane; and (d) inhibition of Na^+^/K^+^ -ATPase activity, leading to reduced Na^+^ transport in the glomerulus because Na^+^ reabsorption in the tubules typically requires active Na^+^/K^+^ -ATPase. The diuretic effect of the n-butanol extract of *A. sativum* could result from one or more of garlic's phytocompounds. Sesquiterpene lactones and triterpenes are known to have diuretic and saluretic effects (208).

The antioxidant metabolites in *C. aurea* seeds, including flavonoids, tannins, and phenolic compounds, are likely responsible for the plant's anti-HTN effects (211–213). Papaya is now recognized as a nutraceutical fruit due to its wide range of health benefits and is favored for weight loss due to its low calorie content compared to other commercial fruits (215, 216). *C. lanatus* seeds also exhibit ant-HTN properties, attributed to their nutrient content, such as Mg, K, and Ca (225). The phenolic compounds in watermelon, including flavonoids, carotenoids, and triterpenoids, offer antiinflammatory and antioxidant benefits. Watermelon is rich in lycopene and β-carotene, which have antioxidant, antiinflammatory, and hypotensive properties. Beta-carotene is significant in reducing the risk of T2DM and lowering the chances of MS (224, 235).

The key bioactive components in *C. aurantium* fruits include phenethylamine alkaloids such as octopamine, synephrine, tyramine, N-methyltyramine, and hordenine. These fruits are also abundant in flavonoids, including hesperidin, neohesperidin, naringenin, naringin, and rutin, which offer various benefits, such as antiinflammatory effects. Essential oils like linalool and limonene contribute to anti-anxiety effects. Citrus fruits are among the best sources of pectin, a type of fiber known for lowering cholesterol and stabilizing blood sugar levels. Pectin also contains growth factor antagonists. Limonene enhances antioxidant detoxification, and it reduces the activity of proteins that may lead to abnormal cell growth (239). Therefore, both pectin and limonene may aid in BP management through their cholesterol-lowering, anti-DM, and antiproliferative properties.

The health benefits of *C. aurantiifolia* are linked to its phytochemicals, which include limonoids, carotenoids, minerals, and vitamins. The extracts of *C. aurantiifolia* contain flavonoids such as rutin, hesperidin, didymin, hesperetin, apigenin, quercetin, kaempferol, nobiletin, and neohesperidin. These flavonoids exhibit hypotensive, vasorelaxant, and antiinflammatory effects. Carotenoids offer protection against severe conditions like heart disease and function as antioxidants while regulating the immune system (239, 588). The vasorelaxant effect, which increases cGMP and cAMP by inhibiting vascular PDEs, can be attributed to its naringenin content (589). The reduction of HTN by citrus leaves may be due to their polyphenol content, especially flavonoids with antioxidant and ACEI activities, such as diosmin, isoquercitrin, hesperidin, eriocitrin, neocriocitrin, narirutin, and 7-OH flavonone (237).

*C. limon* is a valuable source of nutritional supplements, containing a variety of phytochemicals including tannins, terpenes, polyphenols, and carotenoids (13, 590). In studies with rats, both crude flavonoids and fraction B from lemons significantly reduced SBP. These extracts were rich in flavonoid glycosides such as eriocitrin (9), hesperidin, and 6, 8-di-C-β-glucosyldiosmin. Both the crude flavonoids and the flavonoid glycosides demonstrated ACEI effects, with hesperidin (10) showing particularly strong inhibition. Additionally, flavonoid glycosides from lemon peel have been noted for their hypotensive effects (249).

The antioxidant effects of *C. limon* are linked to the presence of hesperidin, eriocitrin, and auraptene. Hesperidin can prevent increases in BP by promoting NO-mediated vasodilation (249). Hesperidin can enhance EF during HTN. It also affects vascular permeability, boosts capillary resistance, and has anti-inflammatory properties (591, 592). Regular consumption of eriocitrin in hypertensive rats has been shown to improve vascular EF. Flavonoids from lemon juice residue help reduce proteinuria in chronic high BP. Long-term administration of auraptene to hypertensive rats also led to a significant reduction in BP (249).

The diuretic properties of *C. limon* juice may be attributed to its components quercitrin and iso-quercitrin (11) because both of these compounds exhibited diuretic effects (13). Diosmetin (12), a flavonoid found in citrus fruits, lemon peel, and *R. officinalis*, has demonstrated anti-HTN effects in rats. Diosmetin's potential to lower BP is evident from its CC antagonism, activation of K^+^ -channels, and vasodilatory effects (593). Hesperidin and diosmin are known to reduce hepatotoxicity, minimize OS, and lower blood sugar and cholesterol levels (251).

The beneficial effects of *C. medica* are due not only to its health-promoting macronutrients and micronutrients but also to its specialized metabolites. These include flavonoids (apigenin, hesperetin, hesperidin, naringin, naringenin, rutin, quercetin, and diosmin), coumarins (citropten, scoparone, and bergapten), terpenes (limonene, γ-terpinene, limonin, and nomilin), and phenolic acids (*p*-coumaric acid, trans-ferulic acid, and chlorogenic acid) (258). The plant's diuretic effects may be linked to dasycarpidan-1-methanol, acetate (ester), which demonstrated a strong binding affinity to PLA_2_ inhibitors in a molecular docking study (257).

The extracts from the leaves and aerial parts of *C. grandis* contain various compounds, including cephalandrol, cephalandrine A, cephalandrine B, β-sitosterol, triacontane, rutin, quercetin-3-*O*-neohesperidoside, kaempferol-3-*O*-rutinoside, kaempferol-3-*O*-neohesperidoside, kaempferol-3-*O*-glucoside, kaempferol-hexoside, oleuropein, and ligstroside (261). The purified polyphenolic fraction from the immature fruit extract of *C. grandis* has demonstrated ACEI activity. This extract yielded eight distinct polyphenols, including hydroxyl ferulic acid, apigenin, isorhamnetin-*O*-sophoroside, and isoscutellarein-*O*-pentoside (265).

The flavonoid fraction of *C. sativum* leaves exhibited anti-lipidemic properties. It also effectively prevented serum NO level reduction in rats, suggesting its potential as an anti-HTN and cardioprotective agent. This supports its use in TM and highlights its potential for development as an anti-HTN drug (274). The flavonoid-rich fraction of *C. sativum* also displayed ACEI activity. This fraction included a range of flavonoids such as apigenin, pinocembrin, pseudobaptigenin, galangin-5-methyl ether, quercetin, baicalein trimethyl ether, kaempferol dimethyl ether, pinobanksin-5-methylether-3-O-acetate, pinobanksin-3-O-phenylpropionate, pinobanksin-3-O-pentanoate, quercetin-3-O-glucoside, apigenin-7-O-glucuronide, apigenin-3-O-rutinoside, rutin, quercetin dimethyl ether-3-O-rutinoside, luteolin, daidzein, pectolinarigenin, and apigenin-C-glucoside (594). The diuretic effect of this plant is attributed to its flavonoids, glycosides, and saponins (272). Identifying and characterizing the active compounds in these metabolites is essential. The major polyphenolics in coriander aerial parts, such as caffeic acid, protocatechuic acid, and glycitin, are primarily responsible for the plant's antioxidant activity (269).

The phenolic acids in *C. macrostachyus* exhibit antioxidant effects (277). The plant's saponins, tannins, and terpenoids may contribute to its diuretic effects by impacting kidney function. Flavonoids and saponins are believed to drive the diuretic activity of the plant by inducing vasodilation in the afferent arterioles of the renal system, which increases the GFR and subsequently enhances urine production (275). The anti-HTN effect of *C. macrostachyus* could also be attributed to its diterpene compounds (277), which have been shown to have vasorelaxant effects when isolated from *Croton zambesicus* (595).

The leaves of *C. citratus* contain essential oils, with citral being the primary component. Citral (13) has been shown to produce an endothelium-independent vasorelaxant effect, likely by influencing intracellular Ca^2+^ levels and partially through NO pathways (596). Both citral and *C. citratus* leaf extracts exhibit spasmolytic properties and may function as Ca^2+^-antagonists (597). Citral has a negative chronotropic effect, likely due to enhanced PNS activity (598). Citral also demonstrates antiproliferative effects (288). It has potential as a novel anti-HTN agent due to its multiple mechanisms of action. Flavonoid and tannin fractions from *C. citratus* have shown vasorelaxant effects on human arteries (284). These substances, along with electrolytes, may have both indirect and direct vasodilatory effects and exhibit CCB properties (45). Therefore, isolating, characterizing, and evaluating the efficacy and safety of these compounds is essential.

The antioxidant activity of lemongrass is likely attributed to its flavonoids, tannins, saponins, alkaloids, and vitamin C. The electrolyte contents of lemongrass, including K^+^, Mg^2+^, and Ca^2+^, may contribute to BP reduction through several mechanisms. For instance, K^+^ helps lower BP by causing vasodilation in SM cells, while Mg^2+^ is thought to lower BP by influencing vascular tone and reactivity. This effect is achieved through changes in the interactions between cellular Mg^2+^ and Ca^2+^ in vascular SM cells, which enhances EF. Mg^2+^ competes with Ca^2+^ for binding sites on membranes, reducing intracellular Ca^2+^ levels and promoting vasodilation. Mg^2+^ and K^+^ might replace Na^+^, contributing further to their hypotensive effects. Therefore, the diuretic, natriuretic, and saluretic actions leading to BP reduction may result from the combined effects of these electrolytes and other active components in lemongrass. ACEI peptides from lemongrass could also serve as potential modulators of BP (45).

The phytochemicals found in *C. citratus*, including alkaloids, tannins, saponins, flavonoids, and phenolics, promote diuresis and natriuresis either individually or through synergistic effects by inhibiting the Na^+^/K^+^/2Cl^−^ co-transporter (NKCC). The minor changes in estimated GFR observed with the extract are similar to those seen with furosemide Rx, supporting the idea that their mechanisms of action are comparable. This co-transporter plays a role in transporting adenosine across the macula densa cell membrane, which is involved in the tubuloglomerular feedback mechanism that maintains volume homeostasis. This action prevents the expected vasoconstriction that might occur with diuretic administration, leading to vasodilation, reduced resistance in the afferent arterioles, and only minor changes in estimated GFR. Saponins' inhibition of aldosterone-sensitive Na^+^ -channels in the cortical collecting tubules suggests they have diuretic properties similar to spironolactone. The flavonoid quercetin inhibited the expression of αENaC-mRNA in the kidney (281). Therefore, further research on these compounds, including their isolation, structural elucidation, and characterization, is needed to evaluate their efficacy and safety.

The interplay between the furosemide-like and spironolactone-like diuretic mechanisms of *C. citratus* phytochemicals could partly explain the minor changes observed in serum K^+^ levels and the modest kaliuresis. The diuretic effect of *C. citratus* may also be influenced by its high K content, which could contribute to the increased serum K^+^ during the acute phase. Research has shown that serum K^+^ can act as a competitive inhibitor of the Na^+^/K^+^ -ATPase pump, and the pump's Na^+^ efflux is inversely related to serum K^+^ levels. The elevated K in *C. citratus* extract might induce diuresis similar to saponins and other phytochemicals, which are known to inhibit Na^+^/K^+^ -ATPase and are present in *C. citratus* (581).

The phenolic compounds found in *F. vulgare* exhibit ACEI activity and anti-DM effects (290). These molecules are linked to the prevention of diseases caused by OS. Fennel is known to contain derivatives of hydroxyl cinnamic acid, flavonoid glycosides, and flavonoid aglycones. Additional phenolic compounds identified in fennel include chlorogenic acid, 3-O-caffeoylquinic acid, 4-O-caffeoylquinic acid, 5-O-caffeoylquinic acid, 1,3-O-di-caffeoylquinic acid, 1,4-O-dicaffeoylquinic acid, 1,5-O-di-caffeoylquinic acid, and 3’,8’-binaringenin (293).

The polyphenol extract from *H. sabdariffa* calyx has shown vasorelaxation effects (296). Hibiscus acid (14), derived from *H. sabdariffa* calyxes, induces vasorelaxation regardless of endothelium presence. Similarly, garcinia acid (15), a diastereoisomer of hibiscus acid, exhibits almost identical vasorelaxant properties. Both compounds likely achieve this by inhibiting Ca²^+^ influx by blocking VGCCs (310). The anthocyanins, delphinidin-3-O-sambubioside (16) and cyanidin-3-O-sambubioside (17), have demonstrated competitive ACEI activity (316). Quercetin glycosides from the plant may also serve as potential Rxs for HTN and CHF. Quercetin has been found to lower BP in hypertensive rats through ACEI activity and by improving EF. Tannins have also been proven to inhibit ACE (599), suggesting the need for bioassay-guided identification and characterization of its compounds. Components such as 4-*O*-methylgallic acid, 3-*O*-methylgallic acid, gallic acid, and cyanidin-3-sambubioside could contribute to anti-HTN effects through different mechanisms (295). The antioxidant properties of *H. sabdariffa* are likely linked to its polyphenolic content (315), and its anti-HTN and cardioprotective effects may stem from the presence of phenolic compounds. The plant's medicinal effects may also be due to its vitamins and minerals (294).

The reduction in Ang-II levels caused by ACEI from *H. sabdariffa* leads to decreased genetic expression of αENaC. The diuretic effect of sour tea and 5AN:5M water extract occurs through reduced αENaC expression, likely due to the presence of flavonoids, as quercetin significantly lowers αENaC expression. In kidney cells, trans-epithelial Na^+^ hypotonicity stimulates Na^+^ reabsorption by increasing αENaC subunit mRNA expression and decreasing intracellular Cl^−^ concentration. Quercetin, by activating the NKCC, raises cytosolic Cl^−^ concentration, suppressing αENaC subunit mRNA expression (600). *H. sabdariffa* and ACEI have nephroprotective effects, slowing renal insufficiency progression. The increase in creatinine clearance in the *H. sabdariffa*-treated group is largely due to anthocyanins, which raise GFR by inhibiting Ang-II production. Vasodilatory compounds in sour tea, like eugenol and quercetin, may enhance creatinine clearance by improving renal blood flow and increasing GFR (299).

The barley and malt extracts of *H. vulgare* contain polyphenols such as catechin, epicatechin, gallocatechin, procyanidin C2, procyanidin B3, and prodelphinidin B3, along with tocopherols (α, *δ*, and γ) and carotenoids (lutein and zeaxanthin), all of which possess antioxidant properties (601). Other polyphenols isolated from methanolic barley seedling extract, including lutonarin, 3-O-feruloylquinic acid, saponarin, orientin, isoorientin, isovitexin, and various glucoside derivatives, showed ACEI activity. Among these, isoorientin (18) showed ACEI potential (323).

Gramine (19), a non-terpenoid alkaloid primarily found in the *Poaceae* family, including *H. vulgare*, induces vasorelaxation similar to ketanserin, acting mainly by inhibiting 5-HT_2A_ receptors (602). Thus, gramine functions as a vasorelaxant. On the other hand, hordenine, another alkaloid from *H. vulgare*, can cause HTN as it acts as a monoamine oxidase B (MAO-B) inhibitor, increasing NE levels. Therefore, further research is needed to fully explore this plant's potential for anti-HTN effects (603). The plant's antihyperglycemic properties may be due to its antioxidant-rich minerals and fibre-rich carbohydrates, which slow glucose absorption, aligning insulin release with peak blood sugar levels (325). Saponarin, a powerful antioxidant flavonoid found in young green barley leaves, has shown potential for treating oxidative and inflammatory conditions and may also have antiobesity effects (604, 605). The high dietary fibre content, including β-glucan, in barley can reduce the risk of CHD (325).

Flavonoid compounds such as isorhamnetin, chrysoeriol, luteolin-7-glucoside, isorhamnetin 3-*O*-galactoside, quercetin-3-*O*-rhamnoside, kaempferol-3-*O*-rubinoside, and caffeic acid, extracted from the aerial parts of *L. leucocephala*, have shown significant antioxidant activity (606). Quercetin, quercetin-3-*O*-α-rhamnopyranoside, quercetin-3-*O*-α-arabinofuranose, luteolin, myricetin, 3’,4’,7-trihydroxyflavone, and myricetin-3-*O*-α-rhamnopyranoside, identified from the plant's foliage, exhibited stronger antioxidant properties (336). The fatty acids (FAs) hexadecenoic acid and oleic acid isolated from *L. leucocephala* were found to function as α-amylase inhibitors (340).

The main components found in different parts of *L. leucocephala* include 1,2-benzenedicarboxylic acid, mono (2-ethylhexyl) ester; 9,12,15-octadecatrienoic acid, methyl ester (Z,Z,Z); betamethasone; β-sitosterol; androstan-17-one; 3-ethyl-3-hydroxy-, (5a)-; 3-beta-hydroxy-5-cholen-24-oic acid; stigmasterol; ampesterol; 1,2-benzenedicarboxylic acid, diisooctyl ester; lupeol; betulin; and 9,12-octadecadienoic acid (Z,Z), methyl ester. Other isolated compounds from this plant include squalene, astaxanthin, tetratetracontane, (cyclohexane, 1,3,5-trimethyl-2-octadecyl-), oleanolic acid, ethane 1,1-diethoxy, (hexadecanoic acid, 1-(hydroxymethyl)-1,2-ethanediyl ester), (octadecanoic acid 2-hydroxy-1,3-propanediyl ester), oleic acid 3-(octadecyloxy)propyl ester, n-hexadecanoic acid, stearic acid 3-propanediyl ester, hexadecanoic acid methyl ester, vitamin E, O-methyl-d-glucose, and 7,10-octadecadienoic acid, methyl ester. Most of these compounds are reported to have significant biological properties, such as antiinflammatory, anti-HC, and anti-DM effects (607).

The anti-HTN activity of *L. usitatissimum* may be linked to its polar compounds, such as quercetin, nicotinic acid, and nicotinamide (355). Four key components in flaxseed—α-linolenic acid, lignans, fiber, and peptides, or possibly their combined effect—may contribute to its BP-lowering properties. The antiinflammatory action of α-linolenic acid could explain its anti-HTN effect, while lignans may reduce BP due to their antioxidant properties (348). Omega-3 fatty acids in flaxseed also have antithrombotic, vasodilatory, and antiatherogenic properties, benefiting lipid metabolism (608). In older adults, a significant reduction in DBP was noted after six months of consuming a lignan complex (609). The lignan metabolites enterolactone (ENL) and enterodiol, produced by gut microbiota, are likely responsible for the effect on DBP, with END also linked to SBP (348). Flaxseed peptides have been found to inhibit ACE (354).

Some oxylipins derived from arachidonic or linoleic acids have been linked to inflammation, tissue damage, vasoconstriction, and OS. In contrast, epoxyeicosatrienoic acids (EETs), produced from arachidonic acid, act as EDHFs associated with natriuresis and eNOS-activated vasodilation. The enzyme soluble epoxide hydrolase (SEH) rapidly converts EETs into dihydroxyeicosatrienoic acids (DHETs), which reduce vasodilation. SEH also metabolizes epoxyoctadecenoic acids into dihydroxyoctadecenoic acids, which are cytotoxic and pro-inflammatory. Inhibiting SEH has been effectively used to lower BP, reduce HTN-related kidney damage, and minimize infarction size in animal models. It is hypothesized that the α-linolenic acid in *L. usitatissimum* (flaxseed) may inhibit SEH, contributing to its anti-HTN effects (351). Studies suggest that higher omega-6 fatty acid levels in human adipose tissue are linked to lower BP, while omega-3 fatty acids in flaxseed regulate gene expression, reduce serum TGs, and modify CV risk factors. Lignans, such as secoisolariciresinol diglucoside (SDG) (20), found primarily as glucosides in food like flaxseed, act as antioxidants (345). SDG, a long-acting compound, has been shown to lower BP in normotensive rats, likely due to its stimulation of GC activity (610).

Lupine seeds contain high levels of polyphenols, carotenoids, phytosterols, tocopherols, alkaloids, and peptides that possess anti-inflammatory and antioxidant properties (611). A patent has been issued for the use of γ-conglutin, a protein from lupine seeds, in the Rx of T2DM due to its ability to interact with insulin and mimic its effects (612). *L. albus* roots and nodules were found to synthesize NO and L- (14C)-citrulline. Regular consumption of bread enriched with lupine lowers BP, and lupine protein has been shown to reduce the risk of HTN and improve VF in rats. This effect may be related to compounds like polyphenols, proteins, or L-arginine in lupine. Administering lupine protein isolate to rats led to a decrease in SBP by 18.6 mmHg compared to the control group. Increasing L-arginine intake, which is abundant in lupine protein, can enhance NO levels and support BP reduction. Therefore, lupine protein may aid in lowering BP by affecting NO metabolism (12).

Quercetin-3-*O*-neohesperidoside, rutin, and kaempferol-3-*O*-rutinoside were identified as key contributors to the antioxidant properties of *M. azedarach* leaves, pulps, and kernels (366). Chemical analysis of the aerial parts of *M. piperita*, which exhibited antioxidant and antiurolithiatic properties, revealed the presence of phenols, alkaloids, flavonoids, glycosides, saponins, tannins, and terpenes (378). A total of 20 compounds were identified in the essential oil of *M. piperita*, which showed antispasmodic activity. The primary components included menthol, menthone, methyl acetate, isomenthol, isomenthone, *ε*-caryophyllene, neo-isomenthol, and pulegone (372). Phenolic compounds such as caffeic acid, eriocitrin, rosmarinic acid, and luteolin derivatives found in peppermint extract exhibited ACEI activities, with eriocitrin identified as the main phenolic compound responsible for these effects (373).

*M. spicata* contains a wide range of bioactive metabolites, including a variety of phenolic chemicals. Its extracts are rich in hydroxycinnamic acids, such as ferulic acid, caftaric acid, p-coumaric acid, and sinapic acid, as well as caffeic acid and chlorogenic acid (384). The main phenolic compound in spearmint extract responsible for its ACEI effects has been identified as rosmarinic acid (21, 373).

The total alkaloidal salts from *M. oleifera* showed negative inotropic effects on the frog heart. Studies on their mechanism of action confirmed that these alkaloidal salts exhibit CCB activity, warranting further research, structural elucidation, and characterization of the active compounds (613). Two flavonoid glycosides, kaempferol-3-*O*-glucoside (22) and quercetin-3-*O*-glucoside (23), isolated from *M. oleifera* leaf extract, demonstrated significant ACEI activity (400). Bioactive phenolic compounds may interact with disulfide bridges on the enzyme surface, altering its structure and reducing ACE activity (391).

Mustard oil glycosides (Niazinin A (24), Niazinin B (25), Niazimicin (26), and Niaziminin A + B) isolated from *M. oleifera* exhibited hypotensive, spasmolytic, and negative inotropic and chronotropic effects (389). The ethanolic extract also showed hypotensive effects, leading to the isolation of two nitrile glycosides (niazirin and niazirinin) and three mustard oil glycosides, including one isothiocyanate (4-(4’-O-acetyla-α-L-rhamnosyloxy) benzyl isothiocyanate (27)) and two thiocarbamates (niaziminin A (28) and niaziminin B (29)). Among these, the three mustard oil glycosides demonstrated similar BP-lowering effects, while the nitriles did not, suggesting the importance of amide and/or sulfur atoms for hypotensive activity. From the ethyl acetate fraction of *M. oleifera*, four compounds were isolated. Two of these, methyl p-hydroxybenzoate (30) and β-sitosterol (31), exhibited promising hypotensive effects in rats (614). Additionally, *p*-hydroxybenzaldehyde (32), the aglycone of α-L-rhamnosyloxybenzaldehyde from *M. oleifera*, caused a reduction in BP (394).

The flavonoids, biophenols, and isothiocyanates in Moringa offer beneficial effects in treating chronic diseases (387, 398). Compounds such as N, α-L-rhamnopyranosyl vincosamide and quercetin found in Moringa exhibit antioxidant, antiinflammatory, and antiapoptotic properties, which protect heart structure. These substances also promote vasorelaxation and protect the endothelium. Quercetin-3-*O*-glucoside, mahanine, and curcumin in the herbal extract significantly reduce body weight, BMI, LDL, and increase HDL levels. Quercetin-3-glucoside and the fiber content in *M. oleifera* may improve GT by slowing glucose absorption and delaying gastric emptying. Phenolics, glucosinolates, isothiocyanates, syringic acid, gallic acid, and rutin may contribute to the leaves anti-DM activity. Pure compounds from *Moringa* extract improve insulin secretion in the pancreatic islets of mice. Four isolated compounds—vanillin, fluorine opyrazine, methyl-4-hydroxybenzoate, and 4-hydroxyphenylacetonitrile—have been identified and characterized (386). Phenolic acids like chlorogenic acid are known for their anti-DM, anti-DL, and antiinflammatory effects. The tannins in *Moringa* possess anti-AS, antiinflammatory, and antihepatotoxic properties, while saponins have hypolipidemic effects (615). *Moringa* leaves, rich in tannins and flavonoids, can improve capillary resistance, venous tone, and collagen stability (391).

Similar to *M. oleifera*, the anti-HTN and antihyperlipidemic effects of *M. stenopetala* are likely due to the presence of alkaloids and glycosides in its extracts. The main compounds in *M. stenopetala* leaves are flavonoids and phenolic compounds, which have potent antioxidant properties and are involved in various bioactivities (18, 406). The seeds of *M. stenopetala* contain glucosinolates, such as 4(α-L-rhamnosyloxy) benzyl isothiocyanate, which have been shown to exhibit multiple biological activities (616).

The anti-HTN effect of *N. sativa* oil may be linked to its FA content (416), particularly due to linoleic and oleic acids. Similarly, the BP-lowering effect of olive oil is attributed to its high oleic acid content, which influences membrane lipid structure and regulates G protein-mediated signaling, leading to a reduction in BP. Another study found that dietary linoleic acid caused a modest BP decrease in normotensive individuals, likely due to its impact on ionic fluxes in vascular ECs. The polyphenol content of *N. sativa* oil is comparable to that of olive oil, contributing to its BP-lowering effect. The antioxidant and ACEI effects of polyphenols and flavonoids in *N. sativa* essential oil support EF and vasorelaxation, further aiding in BP reduction (40).

*N. sativa* seeds contain over 100 compounds, including dihydrothymoquinone, thymoquinone, dithymoquinone, trans-anethole, thymol, and carvacrol, all of which have demonstrated antioxidant properties (417, 428). Two major components from dethymoquinonated black seed volatile oils, α-pinene (33) and *p*-cymene (34), were found to lower arterial BP and induce bradycardia in rats, likely through the suppression of SNS outflow at the vasomotor center in the medulla (425). Thymoquinone (35), a volatile oil component, reduced both arterial BP and HR, probably through central action in the medulla oblongata, involving activation of 5-HT and muscarinic mechanisms (426). This substance also helps prevent HTN and renal damage due to its antioxidant effects (617). Its vasodilatory effect may be partially mediated by activation of K^+^ _ATP_-channels and blocking of 5-HT, α1-, and ET-receptors (618).

Thymoquinone is known for its ability to preserve kidney function, prevent urinary stone formation, reduce crystal retention in kidney tissue, and promote their excretion through urine (432). It also exhibits cardioprotective effects and has been shown to improve hyperlipidemia and MS in diabetic rats (411, 412). Carvacrol and thymol also serve as cardioprotective agents, with thymol helping to prevent CV complications by lowering LDL, TGs, and apoptotic proteins while raising HDL levels. Both thymoquinone and thymol possess anti-obesity and anti-inflammatory properties. Thymoquinone, anethole, p-cymene, and the FAs in *N. sativa* have demonstrated anti-DM effects (411).

In *P. edulis* fruit, the predominant antioxidants include vitamin C, β-carotene, and γ-tocopherol (449). The anti-HTN effect of *P. edulis* may be attributed to its polyphenolic components, as luteolin extracted from the fruit significantly reduced SBP in rats. Flavonoids like luteolin (36) have vasodilatory effects, potentially through PKC inhibition or reduced Ca^2+^ uptake. *P. edulis* leaves contain GABA, a known substance that lowers BP (445, 619). Two compounds, edulilic acid and an anthocyanin fraction, extracted from *P. edulis* also significantly reduced BP and HR in rats, with more pronounced effects than the whole extract. This suggests that the full extract might be less effective due to competitive inhibition for absorption or interference by other components (446).

The anti-HTN and anti-DM effects of *P. edulis* fruit peels are likely due to the flavonoids orientin (37), isoorientin, and pectin (451). Orientin has a vasorelaxant effect (620), while isoorientin helps lower BP, increase coronary flow, and reduce myocardial O_2_ consumption and coronary artery resistance (452). Pectin contributes to its hypoglycemic effects by lowering glucose levels and improving GT (451). Insoluble fiber from *P. edulis* seeds also exhibits hypolipidemic properties (441). The ACEI activity may be attributed to flavonoids such as schaftoside, isoschaftoside, and isovitexin (453). The two major polyphenolic compounds in passion fruit seeds, piceatannol (38) and its dimer scirpusin B (39), showed antioxidant and vasorelaxant effects by promoting NO release from the endothelium, with scirpusin B being more effective than piceatannol (621). Piceatannol also demonstrated anti-DM and antiglycantic activities (438). Chrysin (40), a flavonoid in passion fruit (439), may contribute to the plant's vasodilation effect (449, 451) through multiple signaling pathways (622). Chrysin isolated from *Passiflora incarnata* showed antioxidant activity (623).

The volatile (aromatic) compounds in passion fruit, including 2-tridecanone, (9Z)-octadecenoic acid, 2-pentadecanone, hexadecanoic acid, 2-tridecanol, octadecanoic acid, and caryophyllene oxide, exhibited antioxidant effects. Isoorientin, orientin, spinosin, vicenin-2, α-tocopherylquinone, polysaccharide, luteolin-8-C-β-digitoxopyranoside, and luteolin-8-C-β-boivinopyranoside possess antiinflammatory effects. *P. edulis* contains alkaloids such as harmidine, harmine, harmane, and harmol, with harmine displaying anti-inflammatory activities. Thirteen carotenoids, including violaxanthin, β-cryptoxanthin, neurosporene, lycopene, mutatocrome, β-citraurin, prolycopene, β-carotene, phytoene, neoxanthin, phytofluene, and antheraxanthin, have been identified in *P. edulis* fruits. Carotenoids are known for their health benefits, including antiobesity and anti-DM effects (441).

Steroid and triterpene glycosides in avocado are likely responsible for the plant's hypotensive effects (456). Tannins found in avocado extract exhibit vasorelaxant properties, most of which are endothelium-dependent, similar to Ach. The vasorelaxant actions of polyphenols, including tannins, are linked to the inhibition of PKC and PDEs and/or reduced Ca^2+^ influx (43). Flavonoids and terpenoids were also detected in avocado, contributing to its ACEI activity (465). Further research is needed to isolate, elucidate the structure, and characterize these active metabolites—tannins, flavonoids, and steroid and triterpene glycosides—in avocado.

The anti-HTN and diuretic effects of 70% ethanol leaf and nanoparticle extracts from *P. americana* may be attributed to their quercetin content. Quercetin promotes diuresis by inhibiting Na^+^ and K^+^. It can block the conversion of Ang-I to Ang-II, which increases OS by lowering antioxidant levels. The K and Mg in the extract also contributed to its anti-HTN properties. Both SBP and DBP were lowered as quercetin enhances NOS activity in ECs, stimulating the release of EDRFs, which cause vasodilation (466). Avocado extract boosts the synthesis and release of EDRFs (461).

Avocado fruit pulp contains up to 33% oil, primarily consisting of monounsaturated FAs, which are thought to influence the FA composition in cardiac and renal membranes and improve the absorption of α/β-carotene and lutein. The oil and its FA components are also credited with hepatoprotective effects (43). The ability of avocado to reduce cholesterol and LDL levels is linked to its high monounsaturated FA content, along with β-sitosterol, lycopene, and carotenoids like α-carotene, β-carotene, and tocopherols. β-sitosterol helps maintain healthy cholesterol levels by inhibiting cholesterol absorption, while tocopherols act as natural antioxidants, preventing LDL-receptor oxidation and promoting cholesterol uptake into tissues. Avocados also contain three times more glutathione than any other fruit (468). Some essential oils in *P. americana*, such as β-caryophyllene (41), have shown hypotensive and vasodilatory properties (469). Chromatographic analysis of the ethyl acetate fraction of avocado leaf extract identified 11 compounds, with 11-tetradecyn-1-ol acetate, 8-hexadecenal, 14-methyl-(Z)-, and cyclopropane carboxaldehyde being the most abundant, likely contributing to the fraction's anti-HTN effects (463).

Acetogenins, including persenone A and B, and (2R)-(12Z,15Z)-2-hydroxy-4-oxoheneicosa-12,15-dien-1-yl acetate, isolated from *P. americana*, exhibited antiinflammatory properties. Various phenolic compounds with antioxidant, hepatoprotective, cardioprotective, antiobesity, and antiinflammatory effects have been identified in avocado. These compounds include gallic acid, 3,4-dihydroxyphenylacetic acid, 4-hydroxybenzoic acid, p-coumaric acid, vanillic acid, ferulic acid, (-)-epicatechin, neochlorogenic acid, procyanidins, proanthocyanidins B1 and B2, an A-type trimer, scopoletin, and (E)-chlorogenic acid (also known as caffeylquinic acid, caffetannic acid, helianthic acid, and igasuric acid) (458).

Alongside β-sitosterol, other phytosterols found in avocado pulp and oil include campesterol, stigmasterol, lanosterol, *Δ*5-avenasterol, and *Δ*7-sitosterol. Phytostanols present in avocado oil are cycloarthenol, campestanol, citrostadienol, cycloeucalenol, and 24-methylenecycloartanol. These phytosterols and phytostanols lower cholesterol absorption in the intestines by competing with cholesterol, reducing the risk of heart attack, AS, thrombosis, and other CVDs. Chlorophyll and its derivatives, such as pheophytins and pheophorbide, exhibit antioxidant and antigenotoxic properties. Unlike many other fruits, avocado pulp contains minimal sugar, making it suitable for individuals with DM. Avocados are rich in seven-carbon sugars, primarily D-mannoheptulose and perseitol, with D-mannoheptulose offering protective effects against DM by mimicking caloric restriction, which delays aging and related diseases (459). The high levels of indigestible carbohydrates in avocados help prevent DM and regulate cholesterol. Despite containing carbohydrates, avocados have a low glycemic index (458).

The main compounds in rosemary include monoterpenes (such as 1,8-cineole [eucalyptol], camphor [ketone], α-pinene, borneol, β-pinene, limonene, p-cymene, verbenone [ketone], and β-caryophyllene [sesquiterpenes), diterpenes (including carnosic acid, carnosol, rosmarol, epirosmanol, isorosmanol, and rosmaridifenol), triterpenes (oleanolic acid, ursolic acid, betulin, α-amyrin, and β-amyrin), flavonoids (luteolin, apigenin, genkwanin, diosmetin, hispidulin, 5-hydroxy-7, 4'-dimethoxy-flavone, and cirsimaritin), and phenolic acids (rosmarinic, chlorogenic, and caffeic acids) (624). Compounds like chlorogenic acid, caffeic acid, oleanolic acid, eugenol, and α-pinene have been confirmed to exhibit antioxidant properties. Caffeic acid, carnosic acid, oleanolic acid, rosmarinic acid, carnosol, luteolin, camphor, and eugenol have demonstrated anti-proliferative effects. The anti-inflammatory properties of *R. officinalis* are attributed to carnosic acid, carnosol, eucalyptol, eugenol, and luteolin. Carnosic acid, ursolic acid, camphor, and carnosol have shown anti-DM activity in various studies, with carnosic acid also displaying anti-lipidemic effects. Ursolic acid was found to reduce weight gain and AS, while rosmarinic acid offered protection against AS (470).

From *R. officinalis* leaf extracts exhibiting vasodilatory effects, compounds such as rosmarinic acid, epirosmanol methyl ether, genkwanin, carnosol, carnosic acid, rosmarinic acid-3-O-glucoside, and homoplantaginin were identified. Terpenoids like epirosmanol methyl ether, carnosol, a carnosol isomer, augustic acid, and carnosic acid were suggested to contribute to rosemary's vasorelaxant properties. The vasorelaxant effects of carnosic acid (42) and carnosol (43) were confirmed (471). While rosmarinic acid from *R. officinalis* did not exhibit vasorelaxant properties, its ester derivative, ethyl rosmarinate (44), induced vasorelaxation through an endothelium-independent mechanism, indicating that esterification of rosmarinic acid may enhance its vasodilatory effects (625). The anti-HTN effect of rosemary essential oil was linked to the presence of compounds like 1,8-cineole, α-pinene, camphor, bornyl acetate, borneol, camphene, α-terpineol, limonene, β-pinene, β-caryophyllene, and β-myrcene (626).

Research on *R. abyssinicus*, which demonstrated diuretic effects, identified anthraquinones, flavonoids, saponins, steroids, terpenoids, tannins, phenolic compounds, quinones, and cardiac glycosides as its key components (46, 478). Studies on other plants have shown that flavonoids, sesquiterpene lactones, triterpenes, tannins, saponins, and organic acids possess diuretic properties (315). Physicon, an anthraquinone derived from *R. abyssinicus*, exhibited prominent antioxidant activity (478). Further research is needed to isolate and elucidate the structure and characterize the active metabolites of this plant based on these observed effects.

A phytochemical analysis of *R. chalepensis* leaf extracts, which showed anti-HTN effects, confirmed the presence of flavonoids, coumarins, sterols, and alkaloids (496). Phytochemical screening of extracts from the aerial parts that exhibited hypotensive activity revealed the presence of chromophores, polyphenols, saponins, phytosteroids, withanoids, tannins, anthraquinone glycosides, and cardiac glycosides (10). In various solvent extracts of *R. chalepensis* leaves, compounds such as 2,4-diamino-6-methyl-1,3,5-triazine (an anti-inflammatory agent), quinoline, 1,2,3,4-tetrahydro-2,2,4,7-tetramethyl- (with anti-DM, antiinflammatory, antioxidant, and antihyperlipidemic properties), dioctadecyl disulfide (antiinflammatory), cobalt phthalocyanine (antioxidant and reducing agent), and hydrazinecarbothioamide (antioxidant) were identified as key constituents (627).

*Salvia tiliifolia*, native to the Americas, has been suggested to possess anti-HTN properties in Ethiopia (83). While no preclinical or clinical studies have yet confirmed its anti-HTN effects, tilifodiolide (45), a diterpene compound derived from this plant, has demonstrated vasorelaxant effects through NO and cGMP pathways in isolated rat aortas (628). Supporting this, other species of the *Salvia* genus, such as *S. aucheri* and *S. elegans*, have shown anti-HTN properties (629, 630), while *S. scutellarioides* and *S. officinalis* exhibited diuretic effects (631, 632). *S. tiliifolia* has been reported to possess antioxidant, antiinflammatory and anti-DM activities (628).

The analysis of the aerial parts of *S. punctata*, which demonstrated anti-HTN effects, led to the isolation of two phenolic compounds: rosmarinic acid and linarin. Rosmarinic acid caused a significant reduction in MAP in normotensive guinea pigs. This compound, also extracted from other plants, has been reported to possess various pharmacological properties, including antioxidant, antiinflammatory, LDL oxidation inhibition, and antiangiogenic activities (489).

Steroidal triterpenes of the euphane type were isolated from *S. molle*, which exhibited ACEI activity (501). Compounds such as 2"-α-L-rhamnopyranosyl-hyperin, quercitrin 3-O-β-D-neohesperidoside, miquelianin, quercetin 3-O-β-D-galacturonopyranoside, isoquercitrin, hyperin, and hyperin 6"-O-gallate, separated from the leaves, demonstrated radical-scavenging properties (633). The sesquiterpene hydrocarbon terebinthene was isolated from *S. molle* resin (634). Biflavanone, chamaesjasmin, triterpenes, 3-epi-isomasticadienolalic acid, and isomasticadienonalic acid from *S. molle* showed antiinflammatory activity (495).

Phytochemical screening of *S. nigrum* extracts revealed the presence of phenols, flavonoids, glycosides, alkaloids, saponins, steroids, tannins, terpenoids, triterpenoids, phlobatannins, hydrocyanic acid, and phytic acid (515, 517, 635). From *S. nigrum* fruit extracts that displayed vasorelaxant activity, compounds such as hydroxylamine, O-(2-methylpropyl); 1,2-benzenedicarboxylic acid, diisooctyl ester; 13-docosenamide, (Z); and 1,6:3,4-dianhydro-2-deoxy-β-D-ribo-hexopyranose were isolated (506, 514). Steroidal compounds, particularly steroidal glycosides, are considered the main bioactive constituents of *S. nigrum*, responsible for its various pharmacological effects, including antiinflammatory activity (504).

Lignanamides, such as cannabisin F, are known for their antiinflammatory effects. Scopoletin (46), a coumarin compound found in *S. nigrum*, exhibited antiinflammatory, hypoglycemic, and hypotensive effects. Polyphenols derived from *S. nigrum* are reported to have antiobesity benefits. The flavonoids contribute to its antioxidant activity. Benzoic acids with phenolic hydroxyl groups found in *S. nigrum*, such as 2,4-dihydroxybenzoic acid, gallic acid, protocatechuic acid, vanillic acid, 4-hydroxybenzoic acid, salicylic acid, and 2,5-dihydroxybenzoic acid, mostly exhibited antiinflammatory and antioxidant effects. Polysaccharides from *S. nigrum*, including mannose, glucose, galactose, arabinose, rhamnose, galacturonic acid, and xylose, demonstrated liver-protective properties (504).

Extracts of *S. guineense* were found to contain polyphenols, alkaloids, saponins, steroids, cardiac glycosides, flavonoids, tannins, and coumarins (636). Analysis of the leaf extract with anti-DM effects revealed the presence of rutin, kaempferol-3-O-rutinoside, quercitrin, and quercetin (637). Chromatographic screening of berry leaf extracts, which demonstrated organ-protective and antioxidant effects, identified phenolic compounds such as apigenin, 3,4-OH benzoic acid, caffeic acid, catechin, eugenol, gallic acid, O-coumaric acid, P-coumaric acid, tyrosol, tyrosol-OH, vanillic acid, and syringic acid (525). Organic acids and hydrocarbons were the major compound classes identified (638). Among the polyphenols in the berry leaf extract, ellagitannins like casuarictin and casuarinin and gallotannins such as pentagalloyl glucose exhibited the highest radical scavenging activity (639).

In the anti-DM extract of *T. indica* fruit pulp, saponins, glycosides, alkaloids, anthraquinones, and proteins were identified (534). The extract from the pericarp of ripening *T. indica*, which showed anti-HTN activity, tested positive for proteins, alkaloids, tannins, saponins, steroids, triterpenoids, polyphenols, and flavonoids. Among the biometabolites of tamarind, γ-sitosterol (47) was found to be the most effective ligand for managing HTN, demonstrating drug-likeness with a binding energy of −9.3 kcal and significant ligand-receptor interactions with the GC. Gamma-sitosterol consistently affected fourteen targeted genes, including *NR3C1*, *PPARG*, *REN*, and *CYP11B1*, contributing to reduced HTN and related risk factors (531). The diuretic activity of *T. indica* fruit pulp has been attributed to its content of Mg, ascorbic acid, Ca, K, and terpinen-4-ol (529).

An analysis of *T. indica* fruit pulp extract identified 37 different compounds. Among these, the bioactives were 5-hydroxymethylfurfural, 3-O-methyl-d-glucose, triethylenediamine, 1,6-anhydro-β-d-glucopyranose, 5-methylfurancarboxaldehyde, 1-(2-furanyl)-1-propanone, levoglucosenone, methyl 2-furoate, 2-heptanol acetate, methyl ester hepta-2,4-dienoic acid, 2,3-dihydro-3,5-dihydroxy-4H-pyran-4-one, n-hexadecanoic acid, 3-methyl-2-furoic acid, cis-vaccenic acid, 5-[(5-methyl-2-fur-2)] furancarboxaldehyde, and O-α-d-glucopyranosyl-(1→3)-β-d-fructofuranosyl-α-d-glucopyranoside. These compounds are known for their various biological activities, such as antioxidant properties (533).

An analysis of *T. indica* sweet extract, which demonstrated cardioprotective properties, revealed 40 compounds. Among these, thymine (48) and 4H-pyran-4-one, 2,3-dihydro-3,5-dihydroxy-6-methyl- (49) exhibited the strongest binding interactions with human AT_1_-receptor antagonists and AT_1_-receptor antagonist complexes with PPARγ agonists. PPARγ, known as a nutrient-sensing signaling receptor, plays a significant role in influencing the nutritional regulation of HTN and MS (534). Flavonoids isolated from *T. indica*, such as procyanidins, catechin, taxifolin, apigenin, luteolin, and naringenin, demonstrated antiinflammatory activity (640). Phytochemical screening of *T. schimperi* leaf extract, known for its anti-HTN effects, identified the presence of phenols, polyphenols, flavonoids, terpenoids, anthraquinones, glycosides, tannins, and saponins (23, 54).

An analysis of *T. serrulatus* aerial parts extract, known for its antioxidant and antihyperglycemic properties, revealed the presence of various phenolic compounds. These include quinic acid, danshensu, yunnaneic acid E, feruloylquinic acid, hydroxyjasmonic acid-O-hexoside, apigenin di-C-glucoside, eriodictyol-O-hexoside, quercetin glucuronide, quercetin-O-hexoside, salvianolic acid C derivative, luteolin-7-O-glucoside, luteolin-O-diglucoside, rosmarinic acid hexoside, rosmarinic acid derivatives, salvianolic acids A, B, and F derivatives, K, isoscutellarein-O-glucuronide, luteolin acetyl dipentoside, methyl rosmarinate, and caffeoyl rosmarinic acid. The essential oil extracted from the aerial parts, which also exhibited antioxidant and antihyperglycemic effects, was primarily composed of phenolic monoterpenes and monoterpene hydrocarbons. These included α-thujene, α-pinene, 3-octanone, β-myrcene, α-phellandrene, α-terpinene, p-cymene, D-sylvestrene, (Z)-β-ocimene, (E)-β-ocimene, γ-terpinene, linalool, terpinen-4-ol, thymol methyl ether, carvacrol methyl ether, thymol, carvacrol, thymol acetate, carvacrol acetate, (E)-caryophyllene, germacrene D, and β-bisabolene (539).

Phytochemical analysis of fenugreek extracts, which exhibited anti-HTN and diuretic effects, identified several compounds. These included terpenoids, flavonoids, alkaloids, saponin, glycosides, saponin glycoside, polyphenols, steroids, steroidal saponin, dioscin, coumarin derivatives, tannins, tannic acid, gitogenin, trigogenin, neogitogenin, homorientin, saponaretin, neogigogenin, vitamins, minerals, and proteins (547, 568, 641). The saponin-rich fraction from *T. foenum-graecum* seeds displayed anti-HTN activity in rats (642). Diosgenin (50), a phytosteroid found in fenugreek, protected against ED and OS caused by a high-fat, high-sugar diet. Diosgenin, along with 4-isohydroxyleucine and galactomannan, is responsible for fenugreek's beneficial effects on MS and T2DM. It directly affects arterial SM cells by regulating their viability, preventing cell migration, and promoting endothelium-dependent vasorelaxation. Diosgenin helps alleviate free FA-induced ED and IR by inhibiting the inhibitor of the nuclear factor kappa-B kinase sub-unit beta (IKK-β) and insulin receptor substrate 1 (IRS-1) pathways (545).

Fenugreek leaves, known for their antioxidant and antiinflammatory properties, contain various phenolic compounds, including oleuropein, chlorogenic acid, pyrogallol, ferulic acid, ellagic acid, coumarin, 4-aminobenzoic acid, 3-hydroxytyrosol, catechol, caffeic acid, β-resorcylic acid, gentisic acid, gallic acid, o- and m-coumaric acid, and sinapic acid. They also contain flavonoids such as catechin, kaempferol-3, 2-p-coumaroyl glucose, hesperidin, apigenin-7-*O*-glucoside, naringin, naringenin, rosmarinic acid, and isorhamnetin, along with isoflavonoids and phytoestrogens like daidzein, genistein, biochanin A, and isoformononetin (643). Key components in fenugreek responsible for its antidiabetic effects include saponins, 4-hydroxyisoleucine, sapogenin, diosgenin, trigonelline, and a soluble fiber rich in galactomannan. Diosgenin, protodioscin (a furostanol saponin), and trigonelline have demonstrated antiproliferative properties, while steroidal saponins and saponins have shown antiinflammatory effects (644). Trigonelline and diosgenin may also serve as prebiotic supplements for treating and preventing hyperlipidemia and IR (645).

The polyphenolic fraction of *V. amygdalina* leaf extract showed promising inhibitory effects on both ACE and renin, suggesting these as possible mechanisms for its anti-HTN potential. The phenolic-rich extracts also inhibited α-amylase and α-glucosidase (646). The biflavonoids in bitter leaf are believed to have chemopreventive properties, as they can neutralize free radicals, enhance detoxification, block stress response proteins, and disrupt the ability of certain transcription factors to bind to DNA (548).

Nutraceuticals derived from *V. amygdalina*, particularly phytosterols like β-sitosterol and β-sitosterol glucoside, have demonstrated cardioprotective and hepatoprotective effects, suggesting potential applications in managing CVDs. β-sitosterol is often used in combination Rxs for atherogenesis and OS (549). The antioxidant activity of flavonoids from *V. amygdalina* has been documented (647). The negative inotropic and chronotropic effects of *V. amygdalina* leaves may be linked to resveratrol (a flavonoid), diosgenin, sulforaphane (an isothiocyanate), and high K levels. Condensed tannins, soluble tannins, indoles, steroidal alkaloids, and cyanogenic glycosides found in *V. amygdalina* leaf extracts may contribute to its effects on reducing HR and contraction strength (551).

Flavonoids such as luteolin, luteolin 7-*O*-glucosides, and luteolin 7-*O*-glucuronide, along with alkaloids found in *V. amygdalina* extracts, may contribute to their diuretic effects. Some flavonoids are known to interact with the A_1_-receptor, which is associated with diuretic activity (47). Compounds like luteolin, luteolin 7-O-β-glucuronoside, luteolin 7-O-β-glucoside, 11α-hydroxyurs-5,12-dien-28-oic acid-3α,25-olide, 10-geranyl-O-β-D-xyloside, 1-heneicosenol O-β-D-glucopyranoside, vernodalol, 3′-deoxyvernodalol, and 6β,10β,14β-trimethylheptadecan-15α-olyl-15-O-β-D-glucopyranosyl-1,5β-olide, identified from *V. amygdalina*, have shown anti-oxidant properties. Additionally, vernoniosides and 3′-deoxyvernodalol exhibited antiinflammatory activity, while hydroxyvernolide and 6β,10β,14β-trimethylheptadecan-15α-olyl-15-O-β-D-glucopyranosyl-1,5β-olide have shown anti-DM potential (548, 550).

Ginger is widely used as a nutraceutical due to its numerous health benefits. Chemical studies have identified over 400 compounds in ginger (561). One of the key compounds, 6-gingerol, has shown a range of biological activities, including anti-oxidant and the ability to improve hyperlipidemia (2). Other compounds, such as zingerone, geraniol, shogaols, gingerdiols, gingerdiones, and dehydrogingerdiones, have also demonstrated antioxidant properties. While 6-gingerol has established anti-DM and renoprotective effects, zingerone and 6-shogaol are noted for their antiobesity activities. Gingerol and its related compounds exhibited antiinflammatory effects, with both 6-gingerol and 6-shogaol acting to inhibit platelet aggregation (561). Ginger rhizome extract, which has been shown to lower BP, contains saponins, flavonoids, and alkaloids (563). The anti-HTN effects of petroleum ether extract of *Z. officinale* rhizome and its toluene fraction may be attributed to gingerols and galanolactone, as these compounds are known antagonists of the 5-HT_3_ receptor (568).

A decoction of *Z. officinale* rhizome, which exhibited negative inotropic and chronotropic effects, was found to contain 6-gingerol (51), 8-gingerol (52), and 6-shogaol (53). Among these, 6-gingerol was 6.5 times more effective as a negative inotropic agent as compared to nifedipine. Conversely, 8-gingerol was 2.2 times more potent than verapamil and 2.8 times more potent than diltiazem. While 6-shogaol, a dehydrated form of 6-gingerol, displayed only minimal negative chronotropic activity compared to verapamil and diltiazem, 6-gingerol and 8-gingerol did not exhibit any chronotropic effects (569). Ginger phenolic compounds 6-, 8-, and 10-gingerol (54) demonstrated endothelium-dependent vasodilatory and Ca^2+^-antagonist activities (571). Bioinformatics analysis revealed that 6-, 8-, and 10-gingerol have good oral bioavailability and exhibited vasodilatory, hepatoprotective, vasoprotective, and antioxidant properties. Molecular docking studies indicated that these gingerol compounds, along with losartan, bind similarly to the AT_1_-receptor. Thus, red ginger shows potential as an oral drug candidate for treating HTN (648).

Oleoresin, a non-volatile pungent or flavoring compound found in *Z. officinale*, demonstrated significant antioxidant activity (649). The bioactive components of *Z. officinale* Var. Rubrum (red ginger) are predominantly terpenes, which may inhibit ACE activity. Analysis of red ginger identified five key chemicals: farnesene, zingiberene, β-sesquiphellandrene (55), α-curcumene, and trans-β-farnesene. *In silico* docking studies revealed that β-sesquiphellandrene had the lowest binding energy of −6.55 kcal/mol with ACE, suggesting that both red ginger extract and β-sesquiphellandrene could be promising candidates for anti-HTN drugs (650). Polyphenols can also inhibit ACE. These compounds are reported to suppress 3-hydroxy-3-methylglutaryl CoA reductase, a key enzyme in cholesterol synthesis in the liver, and intestinal acyl CoA (cholesterol acyltransferase), which is involved in cholesterol absorption. This suggests that the reduction in dietary cholesterol absorption may contribute to the hypocholesterolemic effects of this spice (651).

Overall, the secondary metabolites of plants such as *A. remota*, *C. aurea*, *C. papaya*, *C. macrostachyus*, *M. azedarach*, *O. integrifolia*, *M. stenopetala*, *R. abyssinicus*, *R. chalepensis*, *S. punctata*, *S. molle*, *S. nigrum*, *S. guineense*, *T. indica*, *T. foenum-graecum*, *V. amygdalina*, and *Z. officinale* require further bioassay-guided isolation, structural elucidation, and characterization. After isolation, the compounds should be assessed for their anti-HTN activities and mechanisms of action *in vitro* and in animal models. For plants like *A. hispidum*, *A. aspera*, *C. lanatus*, *C. sativum*, *C. citratus*, *F. vulgare*, *L. albus*, *H. vulgare*, *M. spicata*, *N. sativa*, and *P. americana*, additional work on isolating bioactive metabolites, elucidating their structures, characterizing them, and evaluating their anti-HTN properties is needed.

Compounds already isolated and characterized from *C. aurantium*, *C. aurantiifolia*, *C. medica*, *C. grandis*, and *L. leucocephala* require further preclinical studies to better understand their mechanisms of action and anti-HTN effects. For *A. cepa*, *A. sativum*, *H. sabdariffa*, *L. usitatissimum*, *M. oleifera*, *P. edulis*, *R. officinalis*, *T. indica*, *T. foenum-graecum*, and *Z. officinale*, comprehensive efficacy, pharmacokinetic, and safety studies should be conducted, followed by clinical trials.

Promising compounds from the studied plants include chlorogenic acid, achyranthine, quercetin, quercitrin, allicin, allyl disulfide, diallyl trisulfide, eriocitrin, hesperidin, quercetin, diosmetin, citral, hibiscus acid, garcinia acid, delphinidin-3-O-sambubioside, cyanidin-3-O-sambubioside, isoorientin, gramine, secoisolariciresinol diglucoside, rosmarinic acid, kaempferol-3-O-glucoside, quercetin-3-O-glucoside, oleic acid, linoleic acid, thymoquinone, luteolin, orientin, scirpusin B, chrysin, β-caryophyllene, carnosic acid, carnosol, ethyl rosmarinate, tilifodiolide, γ-sitosterol, thymine, diosgenin, 6-gingerol, 8-gingerol, 10-gingerol, and β-sesquiphellandrene. Many of these compounds have multiple mechanisms of action, making them suitable candidates for polypharmacology and network pharmacology approaches to develop novel anti-HTN drugs. Exploring scientifically validated and technologically standardized botanical products from these extracts and compounds through reverse pharmacology could expedite drug discovery.

Comprehensive bioinformatics and chemoinformatics analyses, along with *in silico* studies of these compounds, have the potential to uncover promising drug candidates. Emerging evidence highlights the critical roles of both genetic and epigenetic factors in the regulation and maintenance of BP, as well as in the individual susceptibility to HTN. The interplay between genetic predisposition and environmental influences is increasingly recognized as a key contributor to HTN risk. Notably, epigenetic modifications offer valuable mechanistic insights and represent promising therapeutic targets in the management of CVDs. In this context, herbal medicines demonstrate a strong potential to modulate the onset and progression of chronic diseases through epigenetic mechanisms.

### 5.4 Toxicity profiles and drug-inetractions of antihypertensive medicinal plants

Although plants used in ethnomedicine are generally considered safe, it is crucial to rigorously evaluate their safety when used in scientific research. This is particularly important when the preparation methods diverge from traditional practices, such as using organic solvents instead of aqueous ones. Factors like the plant's age, geographical location, and the timing and season of harvest can influence its phytochemical composition and potential toxicity. For example, flavonoids were found in the older leaves but not in the younger leaves of bitter leaf ethanol extract (652).

The ethanol-soluble fraction of *A. hispidum* did not cause any acute toxic effects. However, teratogenic effects have been observed in female Wistar rats treated with the aqueous extract of this plant, suggesting that it should be avoided during pregnancy. Despite the toxicity found in the seeds and shoots, the roots of *A. hispidum* did not exhibit any toxicity. Root extracts showed neither bactericidal nor mutagenic effects and were non-toxic at doses up to 2,000 mg/kg in rats (150, 154). Toxicity assays using *Artemia salina* indicated that aqueous, hydroalcoholic, methanolic, and dichloromethane fractions and compounds isolated from the aerial parts and leaves of *A. hispidum* were not toxic. Acute and subacute toxicity tests on ethanolic and aqueous extracts from the whole plant revealed no toxic effects, with an estimated average lethal dose of 5,000 mg/kg (149). Although current evidence suggests that *A. hispidum* is relatively safe, it is advisable to conduct subchronic, chronic, and genotoxicity studies before using it for disease management.

In acute toxicity tests, both alcoholic and aqueous extracts of *A. aspera* leaves were found to be safe, with no mortality observed even at a dose of 800 mg/kg (159). Another study by Bhosale et al. established that the toxic dose of aqueous extracts of *A. aspera* leaves and the whole plant exceeds 2,000 mg/kg (162). Additionally, methanolic and aqueous leaf extracts of the plant were shown to protect DNA from damage caused by ultraviolet rays (161). However, *A. aspera* may induce reproductive toxicity in male rats (653).

For *A. remota*, the acute toxicity tests indicated that the median lethal doses (LD_50_) of aqueous and 70% ethanol leaf extracts were above 5,000 mg/kg, suggesting that the extracts are not toxic under the tested conditions (166). Nonetheless, further research is needed, including subacute, subchronic, chronic, and genotoxicity studies, to fully evaluate the plant's safety.

In an acute toxicity study, the LD_50_ of the n-butanol extract from *A. cepa* bulbs was found to be greater than 500 mg/kg in rats (32). Participants taking quercetin-rich onion supplements reported no adverse effects (176). Zaman et al. noted that while the methanolic bulb extract of *A. cepa* did not exhibit teratogenic effects on female mice, it did have anti-fertility effects on male mice, indicating moderate side effects and minimal toxicity (654). Onions can naturally and safely replace a variety of nutraceutical substances and offer good nutritional value (170).

Although *A. sativum* has numerous therapeutic benefits, it can also cause minor side effects such as abdominal swelling, heartburn, flatulence, and acid reflux. Additionally, garlic's antihemostatic effect may pose risks for individuals on anti-coagulant medications, so it is recommended that they avoid garlic while on such prescriptions (3).

The LD_50_ of crude *C. papaya* fruit extract was approximately 325.2 mg/kg in mice (215). The ethanolic extract of papaya root bark was deemed safe at a dose of 2,000 mg/kg in mice (217). Similarly, the methanolic leaf extract of papaya did not show acute toxicity at a dose of 2,000 mg/kg, and no sub-acute toxicity was observed after 28 days at the same dose. There were no significant changes in hematological, hepatic, renal, or cardiac markers following administration of the methanolic leaf extract at 2,000 mg/kg (223). The LD_50_ for the leaf extract was above 5,000 mg/kg, indicating no toxicity in acute studies. However, aqueous extracts of papaya were found to adversely affect the liver and reproductive system in rats (216). Despite its benefits, papaya can have side effects if consumed in excess. It has antifertility properties and contains cyanogenic glucosides that can produce cyanide, which can be fatal. While generally safe as food and medicine for most people, excessive consumption or prolonged skin application can cause esophageal damage, allergies, or irritation. Papaya should be avoided during pregnancy due to potential adverse effects from the enzyme papain on the fetus (219). Further subacute, subchronic, chronic, and genotoxicity studies on various parts of the plant are needed to confirm its overall safety.

Acute toxicity studies on *C. lanatus* extracts were conducted in rodents. The n-hexane extract (seed oil) was found to be safe at doses up to 2,000 mg/kg. Similarly, the aqueous extracts of the roots and leaves were tested in mice, with no deaths observed during the study period. The ethanolic extract showed no mortality up to 2,000 mg/kg when administered orally (231). To ensure the comprehensive safety of this edible plant, further toxicity studies are recommended.

For individuals with fair skin, *C. aurantium* oil may cause photosensitivity, particularly if applied directly to the skin and exposed to strong light. In rare cases, oral consumption of bitter orange has also been associated with photosensitivity. Excessive intake of orange peel has led to severe effects in children, including convulsions, death, and intestinal colic. While some Chinese medicine texts recommend against using bitter orange during pregnancy, the American Herbal Products Association classifies it as “class 1,” suggesting it is safe for use throughout pregnancy when used properly. Bitter orange decoctions have been shown to elevate cyclosporine blood levels toxically in pigs, and *in vitro* studies have demonstrated that it inhibits human CYP-450 3A (CYP3A), potentially affecting the metabolism of drugs processed by this enzyme. A pharmacokinetic interaction involving bitter orange and amiodarone in rats has been reported. Compounds like synephrine and octopamine in bitter orange can cause HTN and arrhythmias, which may lead to severe CV events such as heart attack, stroke, and death. Products containing *C. aurantium* have been linked to serious clinical adverse events, mostly related to CV issues, including syncope, QT interval prolongation, MI, ischemic stroke, angina, tachycardia, bradycardia, hypotension, vasospasm, ventricular fibrillation, and ischemic colitis (239, 242).

In an acute toxicity assessment, the methanol fruit extract of *C. aurantiifolia* was deemed safe in mice at doses below 3,000 mg/kg, but doses exceeding 3,500 mg/kg were found to be toxic to rats (244, 246). Conversely, the methanol fruit peel extract showed no toxicity in mice even at a dose of 5,000 mg/kg. Both acute and sub-chronic toxicity tests of the water extract from roots revealed no signs of toxicity or significant histopathological changes in rats. However, the essential oil contains coumarins, which can cause phototoxicity in humans and have been linked to tumor formation on the skin and foregut of mice. *C. aurantiifolia* juice was found to reduce the number of ova shed in rats, disrupt the estrous cycle, partially prevent ovulation, and alter the histology of the uterus and ovaries, potentially affecting fertility. The juice also has abortifacient effects but does not appear to be teratogenic. Despite these findings, further detailed toxicity studies are necessary to fully establish the safety of this plant for therapeutic use (244).

In acute and sub-acute toxicity studies, *C. limon* juice demonstrated no harmful effects on experimental animals, indicating it is non-toxic and safe for consumption, even at concentrations exceeding 80% (655). When lemon extract was incorporated into animal feed up to the maximum safe level, no adverse effects were observed in the animals (254). Additionally, pre-Rx with lemon essential oil significantly protected rats from aspirin-induced gastric injuries, likely due to the antioxidant properties of the oil (255). However, caution is advised for lemon juice consumers, as there is a potential for pharmacological interactions with anti-HTN medications, and non-adherence to recommended dosages may pose risks (656).

An aqueous leaf extract of *C. grandis* was found to be toxicologically safe at 750 mg/kg in rats (657). In both acute and subacute toxicity studies, no general symptoms of toxicity or mortality were observed within 72 h following the administration of a methanolic leaf extract. Doses between 300 and 2,000 mg/kg did not result in toxicity symptoms in the animals, and no significant changes were noted in biochemical blood markers or organ function. Moreover, there were no abnormal eating or drinking habits or changes in body weight while taking the prescribed doses. Histopathological examinations revealed no abnormalities in the liver or kidneys after Rx with plant extracts at doses between 200 and 800 mg/kg (658). While these studies suggest that *C. grandis* is generally safe, further comprehensive toxicity tests are needed to confirm its safety.

Using Lorke's method for assessing acute toxicity in mice, both the leaf extract and flavonoid-rich fraction of *C. sativum* were found to be non-toxic even at 5,000 mg/kg, indicating the plant's safety at these levels (274). Aqueous and methanol mixtures of coriander fruit extract also showed no signs of acute toxicity when administered orally at doses up to 10,000 mg/kg. The animals exhibited no convulsions or writhing, and reflexes such as the righting and corneal reflexes remained intact (267). The American Plant Products Association classifies coriander fruit as a class I herb, meaning it can be used without concern (659).

A 28-day oral gavage study in rats found that the no-observed-effect level (NOEL) for coriander essential oil is approximately 160 mg/kg per day. In a developmental toxicity study, the maternal no-observed-adverse-effect level (NOAEL) was 250 mg/kg per day, while the developmental NOAEL was 500 mg/kg daily. Although studies on the mutagenicity of coriander and its derivatives have produced mixed results, coriander oil has been confirmed as non-clastogenic, and its main component, linalool, is non-mutagenic. While coriander oil can irritate rabbits, it does not irritate humans, and, unlike the whole spice, it is not considered a sensitizer. The LD_50_ of *C. sativum* essential oil was calculated at 2.257 ml/kg. Research in Italy on *C. sativum* grown as an oilseed crop explored antinutritional compounds such as glucosinolates, sinapine, inositol phosphates, and condensed tannins, which may negatively impact the nutritional value of oilseed residues (269). The U.S. FDA and the Flavor and Extract Manufacturers Association have approved coriander essential oil as a safe food seasoning (659). However, further toxicity studies, including genotoxicity tests, are recommended to confirm its safety.

Acute toxicity studies have shown that aqueous and 80% methanol leaf extracts of *C. macrostachyus* are safe in mice at 2,000 mg and 5,000 mg/kg, indicating that the extract's LD_50_ exceeds 5,000 mg/kg (275). However, toxicity tests on albino mice found the aqueous bark extract to have an LD_50_ of 190.2 mg/kg, while the hydroalcoholic bark extract had an LD_50_ of 87.5 mg/kg. Oral toxicity was assessed in Nubian goat calves using *C. macrostachyus* seeds at 250 mg/kg and 1,000 mg/kg, both of which were fatal in kids between seven and twenty-one days old, leading to toxic effects, including death. Cytotoxicity studies suggest that *C. macrostachyus* extracts could either be harmful or contain beneficial cytotoxic compounds. Therefore, caution is advised when using this plant as an herbal remedy (277).

Cymbopogon is generally safe when consumed orally, applied topically, or used in aromatherapy. However, certain factors, such as heavy metal contamination from the soil, drug interactions, and improper use, can lead to adverse effects (660). Sousa *et al*. (661) reported significant cellular damage, including chromosome aberrations and cell death, due to the incorrect use of *C. citratus*. Similarly, Aberu *et al*. (662) found that lemongrass water extracts (2%, 4%, and 8%) had genotoxic effects on animal bone marrow. Therefore, the consumption of lemongrass tea should be moderated and monitored by health professionals. Toxicological studies indicate that *C. citratus* essential oil has low toxicity and is considered safe for long-term use at doses up to 100 mg/kg. While the USA classifies lemongrass as relatively safe, it is not recommended during pregnancy or breastfeeding due to its ability to stimulate uterine contractions and menstrual flow (660). A combination of *C. citratus* and *B. pilosa* aqueous extracts was found to be mildly toxic in acute and subchronic toxicity tests but caused no damage to vital organs, with an LD_50_ greater than 5,000 mg/kg in rats (663). Lemongrass essential oil and its compound citral showed no toxic effects in animals at 2,000 mg/kg in both subacute and acute toxicity tests (664, 665).

The long-standing ethnomedicinal use of *F. vulgare* without reports of significant side effects suggests it is generally safe. Both acute and long-term toxicity studies have confirmed the safety of fennel extract when taken in recommended doses (293). Nevertheless, one compound, estragole (methylchavicol), has raised concerns. Estragole has been linked to the development of cancerous tumors in rodents, although its potential to cause cancer in humans remains unclear. Numerous studies have suggested that estragole, a component of fennel essential oil, may be genotoxic and potentially carcinogenic. Its carcinogenicity appears to be tissue-, species-, and sex-specific. Recent findings, however, indicate that estragole does not directly cause cancer. Instead, its carcinogenic potential is tied to its metabolic activation, which produces unstable molecules and active radicals that can damage DNA by forming adducts with nucleic acids (666). About 13 compounds isolated from methanolic fennel extract were found to inhibit CYP3A4 activity in the human liver, which could result in unwanted drug interactions, particularly when fennel is consumed alongside anti-HTN medications (293).

In at least 10 countries, infusions and decoctions of *H. sabdariffa* calyx are used to treat hyperlipidemia and HTN, with no reported adverse events or side effects. The LD_50_ for *H. sabdariffa* extracts ranges from 2,000 to over 5,000 mg/kg/day in acute doses and up to 200 mg/kg in chronic three-month studies (300, 667). It is generally well-tolerated by patients (305, 307), and clinical trials have demonstrated 100% tolerability and safety (312). Although mild gastrointestinal symptoms have been noted in some studies, there is no evidence of hepatic or renal toxicity from roselle extract consumption. However, high doses of sour tea have been associated with adverse changes in hepatic and renal biomarkers, and some HC patients have reported severe dysuria. Sour tea at 200 mg/kg in mice has been shown to affect sperm morphology, testicular ultrastructure, and male reproductive fertility (300, 309). Therefore, high doses of *H. sabdariffa* should be avoided when used for therapeutic purposes.

In contrast, administering *H. sabdariffa* water extract for 10 weeks or hibiscus anthocyanins (50 to 200 mg/kg) for 5 days did not affect the male reproductive system in rats. Nonetheless, studies have shown that consumption of sour tea water extract during pregnancy and lactation in rats led to increased postnatal weight gain, delayed puberty onset, and higher BMI at puberty in female offspring. The decreased maternal fluid and food intake during this period, coupled with increased plasma Na^+^ and corticosterone levels, likely contributed to the accelerated growth and delayed puberty in offspring, possibly due to increased corticosterone and reduced leptin transfer through breast milk (668). Another consideration is the potential for herb-drug interactions. For instance, sour tea (20–40 mg/kg) taken with hydrochlorothiazides (10 mg/kg) significantly increased urine volume in rats, raising the risk of dehydration (667). Moreover, sour tea extract can suppress up to 50% of CYP-450 isoforms at doses between 306,000 and 1,660,000 mg/ml, meaning that caution should be exercised when sour tea is consumed alongside drugs (309).

An acute toxicity study established that the LD_50_ cut-off dose for the ethanolic extract of *H. vulgare* seeds is 5,000 mg/kg, indicating its safety at this level (322). Barley is generally considered non-toxic, though it contains certain allergens and anti-nutritional factors, which may pose toxic effects in extreme cases (317). In genotoxicity tests on rats, a polysaccharide fraction from young barley leaves demonstrated safety, with no acute oral toxicity up to 5,000 mg/kg (669). Additionally, *H. vulgare* is reported to have antiapoptotic properties attributed to the presence of 3,4-dehydroxybenzaldehyde, which helps protect against DNA damage (324).

*J. curcas* exists in both toxic and non-toxic genotypes, with the harmless type found exclusively in Mexico and the dangerous type prevalent worldwide. The toxic genotype contains phorbol esters, which mimic diacylglycerol (DAG), a key activator of PKC. This interference with PKC disrupts various cellular processes, including phospholipid and protein synthesis, enzyme activities, DNA synthesis, protein phosphorylation, cell differentiation, and gene expression. Phorbol esters also have purgative, skin-irritating properties and act as cocarcinogens. Accidental ingestion of Jatropha seeds can cause symptoms such as dizziness, vomiting, and diarrhea in humans and can be fatal to various animal species. While defatted Jatropha meal from the non-toxic genotype lacks phorbol esters, it still contains allergens and antinutritional factors (trypsin inhibitors, lectins, and phytates) similar to those in the toxic genotype (328).

The absence of mortality in rats after administering 10–5,000 mg/kg of methanolic leaf extract of *J. curcas* suggests that it is generally safe (335). However, the LD_50_ of the ethanolic (50%) extract of *J. curcas* leaves was found to be 2,500 mg/kg in a study on acute oral toxicity (333). The seed shell extract did not show significant cytotoxicity up to 250 µg/ml (331). But the extract became potentially toxic at >500 µg/ml, resulting in decreased cell viability. Conversely, methanolic kernel meal extract significantly reduced cell viability in all tested cell lines at 6.25 g/ml and higher (329). *Jatropha* species are well-known for their toxic properties, primarily due to their latex and seeds. The latex is highly caustic and irritating to skin and mucous membranes, while the seeds contain toxalbumins that cause agglutination and hemolysis in RBCs, damage other cell types, and have a lipoid resin complex that can induce dermatitis (670). Therefore, caution is advised when using this herb for medicinal purposes.

In an acute toxicity study, 50–2,000 mg/kg of ethanol seed extract from *L. leucocephala* did not produce any significant toxic effects in rats (671). Different fractions and isolated compounds showed no cytotoxic activity against the Ehrlich ascites carcinoma cell line at 25, 50, and 100 µg/ml (606). However, the presence of mimosine makes *Leucaena* species unsuitable for long-term human consumption. Mimosine is an alkaloid known to cause hair loss, growth retardation, cataracts, goiter, decreased fertility, and even mortality in non-ruminant mammals. Despite this, by removing mimosine from the supernatant, it is possible to produce *L. leucocephala* seed protein isolates with relatively low mimosine levels through isoelectric precipitation of seed kernel proteins. These isolates have been used to reduce the risk of mimosine toxicity (339).

*L. usitatissimum* is recognized as safe for consumption by the FDA. Nevertheless, flaxseed can cause mild side effects such as gastrointestinal discomfort, flatulence, and bloating, though these typically subside with a high fiber intake (356). Flaxseed oil is generally safe and well-tolerated, but high doses may lead to diarrhea and loose stools. Allergic reactions can also occur. Individuals with coagulation issues, pregnant and breastfeeding women, and children should exercise caution when consuming flaxseed oil (351).

Although no toxicity has been reported in clinical studies, certain compounds in flaxseed, such as cyanogenic glycosides and linatine, are recognized as potentially toxic. Cyanogenic glycosides—such as linamarin, linustatin, neolinustatin, lotaustralin, and amygdalin—are nitrogenous metabolites that can be converted by intestinal β-glycosidase into cyanohydrin, which breaks down into hydrogen cyanide. Hydrogen cyanide poses a risk of acute cyanide poisoning, affecting the respiratory and nervous systems. However, consumption of flaxseed in 15,000 up to 100,000 mg did not result in elevated plasma cyanide levels above baseline (672).

Another compound, linatine, which has been identified as an antipyridoxine factor in chicks, is also considered potentially harmful. However, clinical studies have not shown flaxseed to cause a vitamin B6 deficiency in humans. Other compounds, such as phytic acid and trypsin inhibitors, have been suggested to negatively impact nutritional status, but there is no evidence from studies showing changes in zinc levels due to phytic acid or any effect on trypsin inhibitor activity after flaxseed consumption. In conclusion, there is currently no definitive scientific data supporting the idea of toxicity from dietary flaxseed due to these compounds. More comprehensive toxicity studies are needed to fully confirm the safety of this plant (672).

*L. albus* is considered toxic when consumed at doses equal to or exceeding 1% of body weight. Sweet lupine, which contains a low concentration of toxic alkaloids (0.003%), poses minimal toxicity risks for both animals and humans. Research using reverse mutation assays and the mouse lymphoma and nuclear assays showed that the ethanolic extract of *Lupinus termis* is not genotoxic. However, *L. albus* seeds, which contain higher levels of alkaloids—specifically quinolizidine alkaloids, a group of about 100 bitter compounds—can lead to cramps, vomiting, and even death due to respiratory paralysis. In humans, consuming excessive amounts may also result in tremors and convulsions. Debittered seeds of *L. albus* are used therapeutically for oral consumption, with a simple detoxification process involving soaking the seeds overnight in water. This helps dissolve water-soluble toxins and reduces the seeds’ bitterness (357). White lupin seeds also have a low content of proteins with antinutritive properties (673).

At doses ranging from 250 to 2,500 mg/kg, neither the ethanol nor water extracts of *M. azedarach* leaves caused death or noticeable signs of general weakness in rats. As a result, 2,500 mg/kg was established as the LD_50_ cut-off value (371). Similarly, methanolic leaf extracts at 1,000, 3,000, and 5,000 mg/kg were found to be safe in mice, with no signs of toxicity observed within 24 h (370). The ethanol leaf extract has been shown to protect against H₂O₂-induced cellular damage, demonstrating a DNA protective effect (369). But, according to traditional Chinese medical literature, ingesting six to nine fruits, 30–40 seeds, or 400 grams of bark can lead to *M. azedarach* poisoning in humans. The plant's toxicity in humans is attributed to limonoids (674). While further research is needed to confirm the plant's overall safety, careful consideration of dosage is crucial when consuming its products.

In an acute toxicity study, a 70% methanol extract of *M. piperita* aerial parts was found to be safe at doses as high as 10,000 mg/kg, with no observed signs of toxicity (378). *Mentha* species are recognized for their DNA-damage protection properties (382). However, a review indicates that peppermint and its main constituents—pulegone, menthone, menthol, and menthofuran—exhibit mild toxicity. Despite this, peppermint and its menthol isomers do not pose significant mutagenic, genotoxic, or embryotoxic risks. One critical consideration is the interaction of peppermint essential oil with CYP-450 isoenzymes in the liver of both rats and humans, where it has been shown to significantly inhibit or reduce the activity of these enzymes. This interaction is crucial for drug metabolism and has therapeutic implications. Peppermint essential oil is not recommended for individuals with bile duct obstruction, gallbladder inflammation, or liver diseases. Additionally, caution should be exercised in patients with gastrointestinal reflux or hiatus hernia, as peppermint oil may worsen reflux symptoms (675).

Toxicological studies on *M. spicata* confirm its safety across a range of doses and time periods, supporting its traditional use as a tisane (herbal tea) in TMs. Both acute and subacute toxicity tests demonstrated that *M. spicata* is safe, with an LD_50_ of 13,606 mg/kg. Despite its widespread use, there is limited information on its complete safety profile. Prolonged use at maximum doses may cause specific issues, highlighting the need for further research into the plant's chronic toxicity to fully understand its toxicological profile (384).

*M. oleifera* leaves are considered safe based on toxicological studies in rats, which showed good tolerability without any mutagenic or genotoxic effects (392). Decoctions of *M. oleifera* leaves were also free of side effects or toxicity in hypertensive patients (390). Acute oral toxicity tests in rats indicated safety at doses up to 2,000 mg/kg, with no harm observed on *A. salina* larvae at similar doses (11). Similarly, methanol and ethyl acetate extracts of *M. oleifera* leaves exhibited no toxicity up to 2,000 mg/kg in mice (400). Toxicological studies have demonstrated that the oral LD_50_ of the aqueous leaf extract exceeds 6,000 mg/kg, highlighting its wide margin of safety. Therapeutic consumption is considered safe at doses below 1,000 mg/kg. No adverse effects on hemodynamic parameters, VF, or OS biomarkers were observed in normal rats after administration of *M. oleifera* leaf extract at 60 mg/kg, suggesting it is relatively safe for therapeutic use. However, caution may be warranted regarding potential nephrotoxicity during long-term use, as a slight increase in urea and creatinine levels was reported in rats receiving the water leaf extract orally for 60 days (397).

Most studies suggest that *M. stenopetala* is safe for therapeutic use. Microencapsulated leaf extract of *M. stenopetala* showed no adverse effects in experimental mice, and acute toxicity tests reported no signs of toxicity or death at doses up to 5,000 mg/kg (406). Similarly, an acute toxicity study of the oral delivery of a 5,000 mg/kg hydro-ethanolic extract of *M. stenopetala* leaves revealed no morbidity or mortality in test animals, indicating that the oral LD_50_ is greater than 5,000 mg/kg (408). However, continuous use of *M. stenopetala* extract at doses exceeding therapeutic levels may lead to mild toxicity (676). In an *in vitro* study, ethanol extracts of *M. stenopetala* leaves and seeds exhibited cytotoxic effects, suggesting the presence of toxic compounds extractable by organic solvents or formed during the extraction process (677). High doses of crude *M. stenopetala* extract were found unsafe for pregnant rats, causing significant delays in embryonic and fetal development, reduced maternal weight gain during pregnancy, increased fetal resorptions, and a higher risk of fetal mortality (678). These findings highlight potential toxic and teratogenic effects, warranting caution when considering its use during pregnancy.

In an acute toxicity study, no lethal effects were observed in mice treated with aqueous extracts of *N. sativa* seeds, even at a high dose of 5,000 mg/kg (41). However, in a sub-acute toxicity investigation where mice were administered *N. sativa* aqueous extract daily for six weeks, one mouse died after two weeks of treatment at 6,400 mg/kg. Additionally, at doses of 21,000 mg/kg and 60,000 mg/kg, two and three mice died during the third and fifth weeks, respectively. No other deaths were reported for lower doses (679). When the fixed black cumin oil was administered through PO or IP routes in mice and rats, the acute toxicity studies revealed LD_50_ values of 28.8 ml/kg and 2.06 ml/kg, respectively. In a chronic toxicity study, rats treated with a daily oral dose of 2 ml/kg of black cumin oil for 12 weeks showed no significant changes in vital liver enzyme levels or histopathological alterations. This suggests that *N. sativa* oil is generally well-tolerated with prolonged use, though high doses may pose risks (680).

In an oral acute toxicity test of thymoquinone, the active constituent of black cumin, the LD_50_ was found to be 2,400 mg/kg in mice. A sub-chronic toxicity study involving mice treated with thymoquinone at 30, 60, and 90 mg/kg/day showed no signs of toxicity or death (681). Similarly, no adverse effects were reported in human studies (415–417). Numerous scientific investigations have demonstrated that *N. sativa* and its active compound, thymoquinone, exhibit modest or negligible toxicological effects, with a wide therapeutic margin (682). This supports the safe, long-term use of *N. sativa* in both traditional food and medicinal contexts.

In an acute oral toxicity test, the methanolic extract of *O. integrifolia* leaves, administered at doses up to 5,000 mg/kg, caused no mortality or adverse effects in experimental animals, suggesting that the hydroalcoholic leaf extract is reasonably safe when administered orally to mice. In a sub-acute toxicity test, a slight reduction in packed cell volume was observed in extract-treated groups, which may be attributed to the presence of saponins in the crude extract. Saponins are known to cause hemolysis by increasing the permeability of the plasma membrane (683). In a genotoxicity study, Rx of cells with the chloroform extract of *O. integrifolia* did not result in detectable DNA damage at 0.01–0.5 mg/ml compared to the control, further supporting the plant's safety (684). However, additional sub-chronic and chronic toxicological studies are recommended to fully assess the plant's safety profile.

*P. edulis* is considered non-toxic and safe for daily consumption at usual doses. Clinical studies have confirmed its safety for therapeutic use (444, 447). In acute and sub-acute toxicity experiments, oral administration of ethanol extract from unripe passion fruit peel at 550 mg/kg showed no harmful effects in rats. Similarly, the aqueous extract of *P. edulis* leaves at 2,000 mg/kg was also found to be safe (441). *In vitro* studies revealed that purple passion fruit peel extract reduced DNA damage but did not inhibit cell proliferation. No significant hepatotoxicity was observed in *in vitro* tests of the passion fruit peel extract (444, 447). Some extracts of *P. edulis* have shown cytotoxicity against various cell types (441), highlighting the need for careful consideration of extract types and dosages in therapeutic applications.

The use of synthetic diuretic drugs can damage the stomach, but avocado leaf extract, due to its tannins and flavonoids, protects the gastric mucosa and does not cause such damage (467). For HTN Rx with avocado seed, dosage is crucial; high concentrations could increase cholesterol levels and lead to AS (468). In an acute toxicity study, rats treated with 50–200 mg/kg of *P. americana* aqueous leaf extract exhibited behavioral changes but no deaths within 24 h, except for one rat that died on the third day after receiving 200 mg/kg. A pilot study suggested that doses above 50 mg/kg could be lethal, setting the highest screening dose for IP injections at 50 mg/kg (456). The LD_50_ of the ethanol seed extract of *P. americana* was determined to be 707 mg/ml in rats (37). Another study found that the LD_50_ of the aqueous seed extract could not be established even at a maximum dose of 10,000 mg/kg. Subacute Rx with the aqueous extract did not affect body weight or organ-to-body weight ratios, indicating safety at this duration. However, extremely high doses are not advisable (685). Additionally, an acute toxicity test showed that 80% methanol avocado seed extract did not cause death or overt toxicity signs at any tested doses in mice, suggesting an LD_50_ greater than 4,000 mg/kg (686).

The LD_50_ of IP-administered ether seed extract of *P. americana* was determined to be 751.6 mg/kg. In a sub-acute toxicity study, daily IP administration of *P. americana* seed extract at 75 and 150 mg/kg led to a significant reduction in food intake, body weight, blood glucose, hemoglobin, and hepatic cholesterol, while serum creatinine, uric acid, total protein, and total bilirubin levels remained unaffected (687). Higher doses of the seed extract (500, 1,000, and 2,000 mg/kg) resulted in death rates of 20%, 60%, and 80%, respectively. No mortality was observed in groups given 125 and 250 mg/kg doses or in the control group. The LD_50_ of the avocado seed extract was thus calculated to be 1,200.75 mg/kg. At 250 mg/kg, the extract was tested for genotoxicity using the micronucleus test, which showed no genotoxic effects (688).

Rosemary extract has been recognized by the European Union as a safe and effective natural antioxidant for food preservation (475). The essential oil of *R. officinalis* has an LD_50_ greater than 2,000 mg/kg in both skin and oral acute toxicity studies conducted on mice and rabbits. In acute skin irritation tests, a 10% rosemary oil ointment did not induce acute toxicity. In sub-acute toxicity tests, rosemary essential oil did not cause mortality or significant gross or biochemical abnormalities (689). Although rosemary oil is considered safe when used in recommended doses, it is not advised during pregnancy due to the potential toxicity of some components (624). For example, d-camphor, a component of rosemary oil, has shown embryotoxic effects in rats and rabbits. There is also limited safety data regarding its use during breastfeeding and in children under 12. Cineole, a major constituent of rosemary oil, can induce liver metabolic enzymes in animals, which may affect the metabolism of other prescription medications (690). Therefore, caution is advised when using rosemary oil in the presence of liver conditions, such as bile duct obstruction, cholangitis, liver disease, gallstones, or other biliary issues, and medical guidance is recommended (624).

In oral acute toxicity tests, neither the aqueous nor the 80% methanolic extracts of *R. abyssinicus* rhizomes showed overt toxicity, with an LD_50_ estimated to be greater than 5,000 mg/kg (476). Mugisha *et al*. reported an LD_50_ of 7,727 mg/kg for a 70% ethanol leaf extract of *R. abyssinicus* in mice (691). In subacute toxicity tests, rats treated with the ethanol leaf extract at doses up to 1,500 mg/kg showed no clinical signs of toxicity, no mortality, and no significant changes in body weight or hematological parameters. However, prolonged exposure to higher doses could potentially cause changes in histopathological, biochemical, and hematological parameters. In another study, subacute toxicity testing with a 2,000 mg/kg dose of hydro-ethanolic rhizome extract of *R. abyssinicus* did not result in any deaths but did show a significant decrease in platelet count and mild acute liver injury (692).

The 80% methanolic rhizome extract of *R. abyssinicus* did not show any toxicity signs in acute toxicity studies, and subacute administration for 28 days resulted in no significant changes in hematological or biochemical markers or overall body weight (693). The maximum (10%) concentration of an ointment formulated from the methanolic rhizome extract, administered at a 2,000 mg/kg limit dose, was also found to be safe in acute dermal toxicity studies (694). Despite these findings, *R. abyssinicus* has not been fully evaluated for its safety as an herbal medicine. Further research is needed to thoroughly assess the toxicity of its extracts, fractions, and isolated compounds for pharmaceutical use. Plants from the Polygonaceae family, which may include *R. abyssinicus*, can contain high levels of oxalic acid. Consuming large amounts of oxalic acid can cause serious health issues. To reduce the risk of poisoning, it is recommended to avoid ingesting the cooking water of these plants (695).

In acute toxicity studies, the LD_50_ of *R. chalepensis* water extract and its ethyl acetate fraction was determined to be 585 mg/kg. High doses of this natural product could potentially cause neurotoxic effects, so caution is advised when using it in TM (696). Conversely, no toxicity was observed in rats after administration of methanol, acetone, or aqueous leaf extracts at 2,000 mg/kg (697). Rue essential oil has an LD_50_ greater than 5,000 mg/kg (698). Ethanolic extracts from the aerial parts of *R. chalepensis* caused no significant acute or chronic mortality in mice at 3,000 mg/kg. Nevertheless, these extracts did lead to significantly lower RBC levels. The extracts did not show spermatotoxic effects (699), although Ruta plants are known for other toxic effects. Contact dermatitis and phototoxicity are common issues linked to all Ruta species, often due to furoquinoline alkaloids and furocoumarins that can cause mutagenicity upon prolonged sunlight exposure (484). Chronic toxicity studies of *R. chalepensis* aerial part aqueous extract at doses of 100, 300, and 600 mg/kg in rats revealed no deaths or significant changes in body weight. However, male fertility parameters showed notable reductions, including decreased weight of the testis, epididymis, and seminal vesicle, as well as reduced sperm count and motility at doses of 300 and 600 mg/kg (700).

Acute toxicity studies on the crude water extract of *S. punctata* aerial parts, administered at 2,000 mg/kg, showed no significant changes in behavior or mortality in mice; all mice remained physically active (490). Similarly, no mortality or significant weight loss was observed in rats administered ethanol (70%) extracts of *S. punctata* aerial parts at 125, 500, and 2,000 mg/kg in both acute and subacute toxicity tests. However, this extract did cause a notable drop in platelet count and minor acute liver injury (692). In contrast, the essential oil from *S. punctata* exhibited higher cytotoxicity, with a 50% cytotoxic concentration of 0.013 nl/ml against human monocytic leukemia cells, and also demonstrated hemolytic activity on erythrocytes (487). These findings suggest that while some aspects of *S. punctata* appear to be safe, particularly in the crude extract form, its essential oil shows significant cytotoxic and hemolytic effects. Comprehensive toxicity studies are needed to fully assess the safety of this plant for various uses.

The acute oral toxicity test of *S. molle* seed extract at 2,000 mg/kg did not reveal any physical or behavioral abnormalities, nor did it cause any fatalities. This suggests that the LD_50_ of the seed extract is higher than 2,000 mg/kg (701). While there were no clear signs of toxicity, mice treated with fruit essential oils at doses of 1,000 and 2,000 mg/kg displayed passive behavior during the first 24 h. The LD_50_ for both the leaf and fruit essential oils exceeded 2,000 mg/kg. By the end of the experiment, no significant toxic effects or organ weight changes were observed in the treated animals when compared to the control group. However, the median lethal concentration (LC_50_) for the leaf and fruit essential oils was 47 and 67 mg/ml, respectively, indicating a higher level of toxicity toward *A. salina*. Overall, the essential oils were more toxic to *A. salina* than to mice (494).

Immediate dermal exposure to ethanolic and hexanic extracts from *S. molle* leaves caused only mild skin irritation and a slight stimulating effect on rats, suggesting the safety of these extracts for topical use (499). Subchronic exposure to the ethanolic extracts from *S. molle* leaves and fruits appears to be safe and may be beneficial for treating lipid disorders (702). In toxicity studies, both acute (2,000 mg/kg) and subacute (1,000 mg/kg) exposures led to increased arousal levels in rats. After acute exposure, the landing foot splay parameter increased, while subacute exposure significantly enhanced motor activity in the open field test. These effects were temporary, disappearing within 7 days. Organ function and structure remained unaffected by either exposure. Overall, the findings suggest that *S. molle* ethanolic extracts from the leaves and fruits are relatively safe for therapeutic use (500).

The toxicity of *S. nigrum* varies significantly depending on the specific variety, and experts caution against consuming the berries unless they are from a known edible strain. Two distinct varieties of *S. nigrum* were identified: one with black-colored fruit and the other with reddish-brown fruit. The black fruit variety is considered toxic (515, 632). The presence of steroidal alkaloids, primarily solanine, in *S. nigrum* plays a protective role by deterring animals from consuming its young leaves and fruits, aiding in the plant's survival. Solanine, a toxic compound, strongly irritates the gastrointestinal lining and has been shown to affect embryonic development, leading to risks of miscarriage and stillbirth. Toxicological studies on male mice revealed that solanine disrupts the bone marrow cell cycle, causing an increase in the ratio of cells in the G0/G1 phase while reducing the ratios of cells in the S and G2/M phases, effectively blocking cell progression. This interferes with DNA synthesis, leading to fewer cells entering the G2/M phase. The incidence of micronucleus formation and sperm deformities increased with higher doses of solanine, indicating potential mutagenic and genetic toxicity. Fortunately, the solanine content in the leaves, stems, and fruits of *S. nigrum* gradually decreases as the plant matures, reducing its overall toxicity over time (504, 515).

Boiling *S. nigrum* likely destroys its toxic components, making it edible after cooking. Similar to other nightshade vegetables, *S. nigrum* has been traditionally linked to the aggravation of joint pain in arthritic patients. This effect is believed to be due to solanine, which is found in the green parts of these plants. However, while solanine is thought to be responsible for worsening joint pain, there is currently no scientific evidence specifically linking the consumption of *S. nigrum* to joint inflammation (634).

In an acute toxicity study using hydromethanolic extract of black nightshade at 2,000 mg/kg, no significant changes were observed in the animals’ physical or behavioral characteristics (703). Even at a much higher dose of 21,500 mg/kg administered through gavage to mice for 14 consecutive days, there were no signs of toxicity or mortality. In genotoxicity tests, the mutagenicity of *S. nigrum* juice was assessed using the mouse sperm deformation test, micronucleus test, and Ames test, with all results being negative, indicating no genotoxic effects. Experimental evidence showed that the maximum tolerated dose of an aqueous decoction of *S. nigrum* in mice is 494,400 mg/kg. For humans, consuming typical amounts of black nightshade (30,000–60,000 mg) rarely results in toxic or adverse side effects (504).

The total extract of *S. nigrum* was found to be safe at doses up to 5,000 mg/kg in acute toxicity studies, likely due to the presence of glycoalkaloids complexed with metal ions, aside from Cu²^+^. However, subacute toxicity studies suggested that glycoalkaloids lacking metal ion conjugation contributed to increased toxicity. In 21-day sub-acute studies, the total extract was deemed safe regarding hematological and liver function parameters at oral doses up to 4,000 mg/kg. In contrast, the glycoalkaloid fraction alone exhibited toxicity at doses of 200 and 400 mg/kg (515). While *S. nigrum* showed lower overall toxicity, some effects on liver and kidney function were noted. Future clinical and preclinical studies are necessary to ensure its safe use in medicine, healthcare, and food products.

The methanolic leaf extract of *S. guineense* did not cause death in mice at 2,000 mg/kg, nor were there any noticeable physical or behavioral signs of toxicity, suggesting an LD_50_ above 2,000 mg/kg (704). In another acute toxicity study, no toxicity or mortality was observed, even at doses up to 5,000 mg/kg (637, 705). In subacute toxicity studies, high doses (1,600 mg/kg) of the stem bark extract led to elevated liver enzymes and biochemical markers of kidney damage, indicating potential hepatotoxicity and nephrotoxicity with prolonged use (706). At lower doses (500 and 1,500 mg/kg), no significant changes were observed in behavior, body weight, or hematological and biochemical parameters. There were also no significant differences in the gross and histological appearance of the liver and kidneys between the Rx and control groups. The 80% methanol extract showed no adverse effects in acute or sub-acute Rxs (705). However, in a subchronic toxicity study, administration of a 70% ethanol leaf extract at 1,000 mg/kg resulted in altered food consumption, weight gain, and changes in liver and kidney enzyme levels, suggesting that high doses of *S. guineense* may be toxic (707). Comprehensive chronic toxicity studies are necessary before any medicinal products of *S. guineense* leaves can be approved for sale.

Rx of rats with 1,000 mg/kg of 70% ethanolic *S. guineense* leaf extract significantly prolonged the estrous cycle, reduced the weight of the uterus and ovaries, and lowered the number of live births and total pups. Despite these effects, no notable changes were observed in reproductive indices or the gross morphology and histopathology of the ovaries, uterus, or vagina. This suggests that high doses of *S. guineense* may be toxic, and excessive consumption of the leaves is not recommended (524). In a study on teratogenic effects, the extract did not significantly affect embryos or foetuses at doses up to 500 mg/kg. However, at 1,000 mg/kg, it slowed embryo development, as indicated by reduced crown-rump length, somite count, and lower morphological scores. Therefore, high doses of *S. guineense* should be avoided during pregnancy (708).

In acute oral toxicity studies, ethanolic *T. indica* fruit pulp extract was well-tolerated at doses up to 3,000 mg/kg, with no observed changes in gross behavior, signs of toxicity, or mortality (535). Similarly, oral acute toxicity tests at 3,000 mg/kg and 5,000 mg/kg resulted in no deaths, suggesting an LD_50_ greater than 5,000 mg/kg, classifying it as practically non-toxic and safe (709). In tests using aqueous *T. indica* pulp extract, doses ranging from 900 to 4,500 mg/kg caused no mortality. Nonetheless, higher doses (2,700–4,500 mg/kg) induced some behavioral changes in rats, such as aggressive scratching, anorexia, mild restlessness, and increased sensitivity to sound. Despite these behavioral effects, no significant differences in hematological or toxicological measures were observed when compared to the control group. Histological examinations of the liver and kidneys showed no lesions, and no abnormalities were detected in the gastrointestinal tract, such as congestion or hemorrhage. These findings provide scientific support for the safety of *T. indica* in TM (710).

The ethanol extract of *T. indica* stem bark, along with its fractions, caused brine shrimp mortality rates ranging from 86.70% to 3.30% at 200, 20, and 2 µg/ml. Sub-fractions exhibited death rates between 46.70% and 3.30%. The LD_50_ values were calculated to range from 832 µg/ml to 5,019 µg/ml. In rats, blood levels of alanine transaminase (ALT) and aspartate aminotransferase (AST) were elevated after being administered 25% and 50% of the LD_50_ determined in chicken embryos for the crude extract and fractions (711). In contrast, the aqueous fruit pulp extract of *T. indica* showed no cytotoxicity at concentrations up to 1,000 µg/ml, indicating its safety (526).

When rats were given 5,000 mg/kg of *T. indica* leaf extract, no clear signs of toxicity were observed. Overall, the rats exhibited no major physical changes. However, those treated with a sub-chronic dose (2,000 mg/kg) of *T. indica* had a noticeable reduction in body weight percentages. Despite the administration of the extract, the organosomatic indices for the liver and kidneys remained largely unchanged in all the rats. Liver function tests (LFTs) in toxicological studies revealed significantly higher globulin and AST levels in the chronic group, though other LFT markers were unaffected. In a sub-acute toxicity study at 2,000 mg/kg, degenerative changes in liver cells were noted. The chronic toxicity study showed congestion in the liver cells of rats treated with the same dose. Short-term use of the extract caused reversible, non-lethal toxic effects, while medium- to long-term use led to subtle but fatal toxic effects. The traditional use of this plant in folk medicine may be due to its ability to maintain stable hematological parameters, body weight, and lack of fatalities (712).

Likewise, studies indicated no acute toxicity (at 50 mg/kg) and no sub-chronic toxicity (up to 2,000 mg/kg) with various concentrations of methanolic extracts from *T. indica* fruits (713). In a separate study, when tamarind pulp water extract was administered at doses up to 1,000 mg/kg, no significant abnormalities were detected in blood biochemistry or hematology. This research concluded that prolonged use of *T. indica* pulp water extract at this dose is generally safe and well-tolerated (714).

In the oral acute toxicity test, animals showed no signs of toxicity or behavioral changes after being given 5,000 mg/kg of *T. schimperi* aquaous leaf extract, and all survived throughout the study period (54). However, at 2,000 mg/kg, *T. schimperi* essential oil caused a 50% mortality rate in the treated mice, suggesting its LD_50_ is around 2,000 mg/kg. For up to 14 days after application, an ointment made from *T. schimperi* oil did not cause any adverse skin reactions. In subacute toxicity studies, the essential oil did not significantly affect body weight, nor did it substantially increase serum enzyme levels in the mice. Histopathological analysis of the liver and kidneys showed no serious damage to these organs from the plant (715).

In another acute toxicity study on rats, the LD_50_ for *T. schimperi* essential oil was found to be 1,284.2 mg/kg. According to the WHO guidelines, this classifies the oil as moderately hazardous when taken orally. In a subacute toxicity study, rats showed no significant changes in behavior, gross pathology, body weight, biochemistry, or most hematological markers. However, the hematological analysis indicated that rats receiving 260 mg/kg had notably lower white blood cell (WBC) counts and higher mean corpuscular volume (MCV) compared to the control group. No significant differences were observed in the liver and kidney histopathology between treated and control groups. An *in silico* toxicity analysis using chemical absorption, distribution, metabolism, excretion, and toxicity (ADMET) and vNN-ADMET predictors revealed that none of the essential oil components exhibited cardiac toxicity, Ames mutagenicity (AMES), or cytotoxicity. Only a small percentage of the compounds (1.75%) were found to potentially harm the mitochondrial membrane, 3.45% posed a risk for drug-induced liver injury, and 8.6% showed hepatotoxic potential. Overall, these results suggest that oral administration of *T. schimperi* essential oil up to 130 mg/kg is considered safe (716).

High doses of *T. schimperi* essential oil (130 and 260 mg/kg) negatively affect the development of embryos and fetuses in rats. It causes notable delays in embryonic and fetal growth, reduces the number of implantation sites, and increases fetal resorption, indicating developmental toxicity. Administering higher doses led to a significant decrease in placenta and litter weights. As a result, the use of *T. schimperi* essential oil in excessive amounts is not recommended (536).

The acute toxicity study of *T. serrulatus* aqueous leaf extract revealed no signs of toxicity, with an LD_50_ exceeding 10,000 mg/kg. Most of the evaluated hematological and biochemical parameters, along with body weight, showed no significant changes following chronic oral administration of the extract. However, male mice treated with 600 mg/kg experienced a notable decrease in basophil count, and female mice at both 200 and 600 mg/kg showed a significant reduction in creatinine levels. No deaths occurred, and body weight remained stable compared to the control group. Both the kidneys and liver appeared normal upon gross examination, and histopathological analysis confirmed no abnormalities in these organs (717).

The oral acute toxicity study for the aqueous extract and essential oil from the aerial parts of *T. serrulatus* showed no mortality during the 14-day observation period. Both the aqueous extract and essential oil demonstrated no noticeable side effects at the highest tested dose of 2,000 mg/kg (539). However, the essential oils of *T. serrulatus* and *T. schimperi*, when tested for acute oral toxicity, caused a burning sensation and inhibited growth in mice. The LD_50_ for these essential oils ranges between 2,000 and 5,000 µl/kg. Consuming large quantities of these herbs may be toxic to vulnerable groups, such as children and pregnant women, especially since thyme's aerial parts, rather than the essential oils, are commonly used in teas and as food additives (718).

Several animal and human toxicity studies have investigated *T. foenum-graecum*. In one animal study, the acute oral LD_50_ in rats was found to exceed 5,000 mg/kg, and the acute dermal LD_50_ in rabbits was greater than 2,000 mg/kg. Another study showed that debitterized fenugreek powder did not cause toxicity or mortality in rats and mice during acute (2,000 and 5,000 mg/kg) and sub-chronic (1%, 5%, and 10% in a pure diet) toxicity assessments. Additionally, weanling rats fed fenugreek seeds for 90 days exhibited no significant hematological, hepatic, or histopathological changes. A toxicological evaluation of 60 patients with DM, who consumed 25,000 mg of powdered fenugreek seeds daily for 24 weeks, revealed no signs of liver or kidney damage or blood abnormalities. In its initial human clinical trial, fenugreek extract was deemed safe and well-tolerated (546).

In the acute toxicity test of fenugreek ethanol seed extract at a dose of 3,000 mg/kg, no severe signs of toxicity or mortality were observed, though there was a mild increase in respiration and excitation, indicating that the extract was relatively less toxic at this dose. In a chronic toxicity study, one male mouse developed forelimb inflammation and baldness after 30 and 40 days of Rx, respectively. Two additional male mice experienced inflammation in both forelimbs and hindlimbs between 40 and 60 days of Rx. Survival studies showed significantly higher lethality in the treated groups, and both male and female mice in these groups ceased to gain body weight. Prolonged Rx for 90 days had a minimal impact on organ indices, but there was a significant reduction in testicular weight in the male group. All hematological parameters for both sexes remained normal, but biochemical tests revealed significantly elevated ALT levels in the treated animals. Male mice exposed to fenugreek chronically showed severe spermatotoxic effects (641).

The literature review on fenugreek does not report any clinically significant harmful adverse effects. While fenugreek is generally considered safe and well-tolerated, there are some associated adverse effects. Individuals with allergies to fenugreek or chickpeas should avoid fenugreek due to potential cross-reactivity. Fenugreek-containing curry powder can trigger allergic reactions in those with severe bronchospasm, asthma, or diarrhea. Other reported side effects include occasional diarrhea, flatulence, and dizziness. Blood glucose levels should be monitored following fenugreek supplementation due to the risk of hypoglycemia. Fenugreek can also reduce triiodothyronine (T3) production and lead to weight loss. Coumarin derivatives in fenugreek may increase bleeding risk, particularly when used with antiplatelet or anticoagulant medications, due to elevated prothrombin time (PT) and international normalized ratio (INR). Fenugreek should be avoided during pregnancy as it can stimulate uterine contractions, as seen in animal studies. Therefore, fenugreek should be used in moderation or at specific doses when employed as a therapeutic agent (719).

The structural characteristics of fenugreek fiber can impair the intestinal absorption of oral medications. Thus, fenugreek and related preparations should be taken after prescription medications. Close monitoring of blood glucose levels is necessary when fenugreek is used with hypoglycemic drugs, as it can lower serum glucose levels more than expected. A small study found that a water extract of fenugreek reduced K levels by 14% in healthy patients, suggesting that fenugreek might cause hypokalemia when used with mineralocorticoids, diuretics, purgatives, or other K-lowering medications. Fenugreek is also believed to have estrogenic properties. It was observed to lower serum T3 levels while increasing serum thyroxine (T4) levels. When taken with certain medications, fenugreek can enhance hypokalemic, hypoglycemic, and estrogenic effects. It may also interact with other drugs, so careful consideration of timing and dosage is essential when using fenugreek (719).

In an acute toxicity study of *V. amygdalina* leaf extracts, oral administration of a single dose of 5,000 mg/kg did not produce any harmful symptoms or mortality in rats, indicating an LD_50_ greater than 5,000 mg/kg (652). However, an acute toxicity test of the aqueous leaf extract in rats showed an LD_50_ of 1,265.22 mg/kg (720). Another acute oral toxicity study with the aqueous extract at 5,000 mg/kg revealed no mortality or significant physical or behavioral changes. Both the sighting and main investigations showed no notable changes in body weight, organ weight, or biochemical markers. Given that the toxicity of the aqueous extract may be less than 5,000 mg/kg, it is considered safe for consumption as a vegetable or herb (721). Additionally, the methanol (80%) extract of *V. amygdalina* leaves, administered at 2,000 mg/kg in an acute toxicity study, did not cause any morbidity, neurological, behavioral, autonomic, or physical changes in mice, and no deaths occurred. This suggests that the LD_50_ for the methanol extract could be greater than 2,000 mg/kg in mice (722).

An acute toxicity assay of crude saponin from *V. amygdalina* leaves, administered orally, revealed an LD_50_ of 5,152.3 mg/kg, indicating that it is nearly non-toxic (723). The water extract of bitter leaf demonstrated very low cytotoxicity in brine shrimp assays, suggesting that *V. amygdalina* could be a safe candidate for use as a nutraceutical or functional food (724). Sub-chronic oral administration (28 days) of methanol leaf extract of *V. amygdalina* at doses up to 1,200 mg/kg did not adversely affect liver or kidney functions in rats (725). Exposure of HepG2 cells to chloroform root extract of *V. amygdalina* did not induce significant DNA damage, showing no genotoxic effect at concentrations of 0.01, 0.05, and 0.25 mg/ml. However, in MCF-7 cells, the leaf extract demonstrated a dose-dependent increase in DNA damage, with statistically significant damage observed at 2 mg/ml (684).

Ginger is widely considered a safe herbal drug and is classified as generally recognized as safe (GRAS) by the U.S. FDA. A dosage of 500–1,000 mg of ginger powder taken 2–3 times daily for 3 months to 2.5 years has not been associated with adverse effects. The British Herbal Compendium also reports no adverse effects from ginger. The acute oral LD_50_ for roasted ginger in rats is 170,000 mg/kg, while for dry ginger, it exceeds 250,000 mg/kg (561, 726). Multiple studies confirm ginger's safety across various concentrations (100, 333, and 2,000 mg/kg) and experimental periods, demonstrating that it is non-toxic even during pregnancy in rats and gynecological procedures, as shown in clinical studies (561).

However, ginger tea exposure *in utero* may lead to increased early embryo loss and growth in surviving fetuses. Some minor adverse effects have been noted in humans. In a clinical trial with 12 healthy volunteers taking 400 mg of ginger orally three times a day for two weeks, one participant reported mild diarrhea during the initial days. Doses exceeding 6,000 mg may cause stomach irritation and heartburn. Additionally, inhalation of ginger dust can trigger IgE-mediated allergies (727).

Ginger powder (10%) mixed with a diet, along with two ginger compounds (genistein and 6-gingerol), demonstrated anti-genotoxic effects. Additionally, 6-gingerol showed *in vitro* radioprotective activity (728). The toxicity of gingerol compounds is considered low, with no hepatotoxic effects observed (648). Few studies report interactions between ginger and medications. One study found that ginger did not affect the coagulation status or the pharmacokinetics and dynamics of warfarin in patients (727). The anti-HTN efficacy and plasma concentration of losartan were significantly increased in hypertensive rats when combined with *Z. officinale* and *H. sabdariffa*. This finding suggests potential herb-drug interactions, warranting further research in humans or human liver microsomes (729).

## 6 Policy implications

The Federal Ministry of Health (FMoH) reports that Ethiopia is currently experiencing a triple burden of diseases affecting people of all ages, with non-communicable diseases (NCDs) contributing significantly. The proportion of NCDs has increased from 17% to 35% over the past 20 years. Key risk factors contributing to the disability-adjusted life years (DALYs) lost include malnutrition, dietary risks, alcohol use, and tobacco use. Younger age groups often exhibit higher rates of lifestyle-related risk factors for chronic diseases, leading to increased premature mortality. Adolescents are particularly prone to sedentary lifestyles. Additionally, nearly half of CVD patients lack sufficient knowledge about CV risk factors. There remains a need for ongoing lifestyle and health-related behavior modification (730).

In Ethiopia, the national prevalence of HTN and DM in 2022 was found to be 19.2% and 2.8%, respectively. Significant regional variations were noted, with the highest prevalence of HTN in Addis Ababa (30.6%) and DM in the Somali region (8.7%) (731). To address these issues, the FMoH aims to increase the proportion of hypertensive adults and DM patients with controlled BP and blood sugar levels from 26% to 60% and from 24% to 60%, respectively, by 2025. Additionally, the FMoH seeks to reduce the risk of premature mortality from major NCDs from 18% to 14% by the same year. The ministry plans to achieve these goals through the implementation of policies, legislation, and regulations targeting unhealthy diets, khat use, tobacco, and alcohol consumption (730). We support these strategies, as high salt and fat intake, micronutrient deficiencies, excessive alcohol consumption, and smoking are known risk factors for NCDs, particularly CVDs, including HTN (4, 22). Furthermore, evidence suggests that chewing khat can directly increase BP (130–134).

The FMoH promotes interventions to reduce exposure to environmental and occupational risk factors for NCDs (730). However, mere promotion may not be sufficient to address critical issues like sound and air pollution in Ethiopia. Research shows that exposure to noise and air pollution, including toxins, radiation, and chemicals, can increase BP (732, 733). Therefore, this paper strongly recommends that the Ethiopian government develop and enforce regulations on these environmental issues and implement intervention measures at the kebele level.

One initiative by the FMoH to combat NCDs is the strengthening of nutrition service delivery. They also plan to reduce the prevalence of unsafe and illegal food products in the market from 40% to 30% (730). Ethiopia is rich in various plants with nutritional and medicinal values, which the health sector should focus on. There is a growing interest in natural products, particularly those with antioxidant properties, for disease management. Recent dietary recommendations emphasize increasing the intake of antioxidant-rich fruits and reducing energy-dense snacks. Diet is crucial for BP control, and dietary approaches to prevent HTN are gaining attention. Bioactive phytoconstituents in foods and MPs, such as alkaloids, peptides, flavonoids, anthocyanins, phenolic acids, and polyphenols, are recognized for their preventive and therapeutic benefits for CV health. Nutraceuticals like curcumin, conjugated linolenic acid, psyllium fiber, and polyunsaturated FAs have also been noted for their anti-obesity effects (50, 595).

Among dietary therapeutic strategies, using functional foods that offer health benefits beyond basic nutrition is a viable option (355). In Ethiopia, several anti-HTN plants, such as papaya (Paw Paw Tree), Habhab (Watermelon), Tiringo (Citron), Tikur gebs (Black Barley), Telba (Flaxseed), Gebto (White Lupin), Shiferaw (Moringa), and avocado, are considered functional foods or nutraceuticals with various health-promoting effects. Edible plants like Komtatie (bitter orange), Mekmeko (spinach rhubarb), and Dokma (water berry) also exhibit anti-HTN properties.

Other anti-HTN plants, including Key shinkurt (Red onion), Nech shinkurt (Garlic), Lomi (Lemon and Lime), Dimbilal (Coriander), Roka (Tamarind), Tossign, Abish (Fenugreek), Zinjibil (Ginger), Ensilal (Fennel), Tikurazimud (Black cumin), Tenadam (Rue), Sigametebesha (Rosemary), and Qundo berbere (Pepper tree), are traditionally used as condiments, spices, or flavoring agents in Ethiopian cuisine. Additionally, Kedkedie (sour tea), a traditional beverage and dietary therapy for high BP, shows potential as a nutraceutical and dietary additive for HTN management (636).

In addition to their anti-HTN effects, many of the mentioned plants exhibit antioxidant, antiinflammatory, antiproliferative, and other beneficial activities. These plants have demonstrated efficacy against various conditions, including DM, AS, DL, HTN, obesity, and cancer. Several of these plants are high in dietary fiber, and their intake has been inversely associated with CHD and stroke. Healthful snacking with lower-energy-density foods like watermelon and papaya can enhance satiety and reduce overall energy intake, helping to manage weight.

Given that these plants are consumed as food or used in cooking, they are expected to have low toxicity and only moderate side effects. Therefore, it is crucial to raise awareness about the benefits of these potential food supplements, nutraceuticals, and MPs for reducing NCDs in Ethiopia, especially among pre-diabetic or pre-hypertensive individuals. Increasing their cultivation, preferably in organic forms, would be beneficial for the country's health.

In Ethiopia, there is a concerted effort to strengthen the integration of modern medicine and TM by implementing activities to incorporate TM into primary health care. Strategic activities include (1) improving the conservation and documentation of MPs and TM knowledge and practices, (2) promoting research and development of TMs, including clinical trials, and engaging academia and research institutions, and (3) establishing an incubation center for the laboratory formulation of scientifically validated TMs (730).

This review highlights the importance of these activities. Academia and research institutions should focus on developing anti-HTN vegetables, fruits, and cereals into herbal supplements in forms such as capsules, pills, or tablets. Commercial products like Kedkedie (sour tea) are already available as dietary supplements (295). Employing food technologies such as spray drying and freeze-drying to enhance and preserve the L-citrulline content in watermelon can make its consumption more convenient, potentially improving adherence to daily intake. Watermelon microencapsulation could offer a more effective method for improving vascular health in individuals with cardiometabolic risk factors (235).

Microencapsulation involves packaging extracts and active ingredients in microparticle matrices to create a physical barrier that controls the release of the active compounds. For example, microencapsulated Moringa leaf extract has demonstrated significant anti-hyperglycemic, vasodilator, and diuretic activities (409). Nanotechnology drug delivery systems show promise in CV studies due to their improved pharmacokinetic profiles, biocompatibility, low toxicity, and greater efficiency in targeting mitochondrial dysfunctions. Incorporating Moringa leaf extract with silver nanoparticles has been shown to enhance anti-oxidant activity (386).

Utilizing MPs in the form of oils can be highly effective. For example, the essential oil extracted from Nana (spearmint) has shown significant bioactivity, including antioxidant and antiproliferative effects. This suggests that such bioactive oils could be valuable in both pharmaceutical industries and food production technologies (382).

The use of plant proteins in therapeutic applications is also on the rise. Peptides derived from food proteins, such as those with ACEI and α-amylase inhibition activities, are promising ingredients for functional foods. Hydrolysis of plant proteins, such as those from Ayderke (Physic nut), using enzymatic systems like alcalase, results in bioactive hydrolysates with ACEI activity (328). Similarly, Moringa protein hydrolysate and black cumin seed proteins have demonstrated anti-HTN effects through ACEI activity (5, 427).

Given the diverse MP resources in Ethiopia, academic and research organizations should advocate for networking pharmacology. Establishing reverse pharmacology and networking pharmacology laboratories, along with collaboration with international institutions, could enhance the application of MPs. Training, workshops, and conferences on these topics would be beneficial for advancing research and practical applications in Ethiopia.

## 7 Limitation of the study

Unpublished data from MSc theses and PhD dissertations were excluded from this paper due to ethical concerns. Strict exclusion criteria were avoided to offer readers the most comprehensive information and to acknowledge the efforts of the researchers. Many of the studies reviewed did not involve the isolation, structural elucidation, or characterization of bioactive compounds. Additionally, clinical trials on the MPs studied are scarce, and there is a lack of multiple anti-HTN evaluations and exploration of the mechanisms of action for the extracts and compounds of these plants.

## 8 Conclusion

In Ethiopia's TM, plants play a crucial role in healing, particularly among patients with CV conditions who often turn to herbal remedies more than other forms of complementary and alternative medicine. This research highlights that several MPs with natural compounds have been identified and studied for their potential in developing anti-HTN drugs. Evidence-based approaches are increasingly incorporating these herbs and plants in the prevention and Rx of CVDs. Most research focuses on the effects and mechanisms of these plant extracts using *in vitro*, *in vivo*, and *ex vivo* models. The main anti-HTN mechanisms identified include antioxidant properties, vasorelaxation, NO production, CCB, K^+^-channel activation, suppression of RAAS, intracellular cGMP activation, diuretic effects, and SNS inhibition. Given these diverse mechanisms, herbal medicines are likely to attract more attention in the future, although their current use is hindered by a lack of sufficient clinical trials. Educating patients about the safety and benefits of herbs like black cumin, coriander, garlic, ginger, watermelon, fennel, moringa, avocado, rue, tossing, and fenugreek is important. Among the plants studied, less than half have had their bioactive compounds fully characterized. Promising compounds for anti-HTN therapy include chlorogenic acid, achyranthine, quercetin, allicin, hesperidin, diosmetin, citral, hibiscus acid, eriocitrin, isoorientin, gramine, secoisolariciresinol diglucoside, kaempferol-3-O-glucoside, thymoquinone, luteolin, scirpusin B, chrysin, β-caryophyllene, carnosic acid, rosmarinic acid, γ-sitosterol, diosgenin, gingerols, and β-sesquiphellandrene. The pharmaceutical industry should focus on developing effective drug candidates from these phytochemicals. The demonstrated anti-HTN properties of these MPs underscore the importance of traditional knowledge in HTN Rx. However, around 35 MPs discussed in this review have not been tested for their BP-lowering effects. More research is needed on these plants, including studies on their pharmacological activities, mechanisms of action, bioactive compounds, toxicology, and large-scale clinical trials to confirm their potential in addressing HTN-related conditions and mortality.

## 9 Future development directions

Traditional botanical studies on MPs highlight new opportunities for investigating their anti-HTN properties. Therefore, thorough ethnobotanical surveys are needed to record indigenous knowledge of MPs used to treat HTN. By scientifically validating this traditional knowledge, we can discover new anti-HTN compounds. It is essential to collaborate with local healers and communities to safeguard these traditional practices, potentially by creating digital records of MPs, their applications, and preparation methods. However, the growing demand for MPs in herbal medicine and drug development poses a risk to their sustainability. Thus, it is vital to protect endangered, vulnerable, and overexploited species for future generations, who will be better equipped to manage and utilize these resources responsibly.

Plant-derived natural compounds have long been, and will remain, crucial as sources of therapeutic agents and as models for designing, semisynthesizing, and synthesizing drugs for treating both human and animal diseases. We recommend further studies to assess the pharmacological properties and potential toxic effects of extracts and isolated secondary metabolites from these plants. It is anticipated that plants will continue to produce novel biomolecules, leading to the discovery of innovative Rxs for CVDs. Additional bioactivity-guided fractionation, identification, and characterization of these secondary metabolites should be pursued. Investigating the relationship between the chemical structures of phytochemicals and their biological activity can enhance drug development or synthetic alterations. Comprehensive pharmacological studies are also needed to understand how extracts and bioactive compounds lower HTN. Screening these extracts and compounds for synergistic effects in combination with other plants or anti-HTN drugs is essential. A repository of phytochemicals with anti-HTN potential should be created to facilitate synthetic modifications or pharmaceutical applications. After success in preclinical studies, clinical trials must be conducted to confirm their effectiveness in humans. Future research, including extended randomized trials, could help clarify the long-term effects of MPs.

Nutraceutical products can be developed from MPs by standardizing their extracts or powders for use in dietary supplements and functional foods. These nutraceuticals should be promoted not only for HTN management but also for their broader health benefits, such as antiinflammatory and antioxidant effects. Additionally, incorporating these nutraceuticals into national dietary guidelines can support preventive healthcare through local biodiversity. Ensuring the safety and non-toxic nature of MPs while identifying potential adverse effects or contraindications is essential. To address current challenges, accelerating drug discovery by merging ethnopharmacology with modern computational tools is crucial. The use of reverse pharmacology on traditional MPs and integrating network pharmacology to understand complex biological interactions is strongly recommended for advancing drug discovery today.

## Glossary

*α*ENaC: alpha epithelial sodium channel
5-HT: serotonin
ACE: angiotensin converting enzyme
ACEI: angiotensin converting enzyme inhibitor
Ach: acetylcholine
ADH: anti-diuretic hormone
ADMET: absorption, distribution, metabolism, excretion and toxicity
ALT: alanine transaminase
Ang: angiotensin
AR: arteriolar resistance
ARB: angiotensin receptor blocker
AS: atherosclerosis
AST: aspartate aminotransferase
BB: beta-blocker
BK: bradykinin
BMI: body mass index
BP: blood pressure
Ca: calcium
CA: cardiac arrhythmia
CAI: carbonic anhydrase inhibitor
cAMP: cyclic adenosine monophosphate
CBVD: cerebrovascular disease
CC: calcium channel
CCB: calcium channel blocker
cGMP: cyclic guanosine monophosphate
CHD: coronary heart disease
CHF: congestive heart failure
CHT: cardiac hypertrophy
CO: cardiac output
COX: cyclooxygenase
CRC: concentration-response curve
CV: cardiovascular
CVD: cardiovascular disease
CVS: cardiovascular system
DAG: diacylglycerol
DBP: diastolic blood pressure
DL: dyslipidemia
DM: diabetes mellitus
DPP-4: dipeptidyl peptidase-4
ECs: endothelial cells
EDHFs: endothelium-derived hyperpolarizing factors
EDRF: endothelium-derived relaxing factor
EDV: end-diastolic volume
EF: endothelial function
eNOS: endothelial nitric oxide synthase
ET: endothelin
FAs: fatty acids
FDA: Food and Drug Administration
FMoH: Federal Ministry of Health
FTIR: fourier-transform infrared
GABA: gamma-aminobutyric acid
GFR: glomerular filtration rate
GRAS: generally regarded as safe
GT: glucose tolerance
H_2_S: hydrogen sulfide
HA: heart attack
HbA1c: hemoglobin A1c (Glycated hemoglobin)
HC: hypercholesterolemia
HDLC: high-density lipoprotein cholesterol
HF: heart failure
HO-1: heme oxygenase 1
HP: hyprepolarization
HR: heart rate
HTN: hypertension
ICAM-1: intracellular adhesion molecule 1
IKK-B: inhibitor of the nuclear factor kappa-B sub-unit beta
iNOS: inducible nitric oxide synthase
INR: international normalized ratio
IP_3_: inositol-1,4,5-triphosphate
IR: insulin resistance
IRS-1: insulin receptor substrate 1
K: potassium
LC_50_: median lethal concentration
LD_50_: medial lethal dose
LDLC: low-density lipoprotein cholesterol
LFT: liver function test
MAP: mean arterial blood pressure
MC: myocardial contractility
MCV: mean corpuscular volume
MI: myocardial infarction
miRNA: microRNA
MP: medicinal plant
MS: metabolic syndrome
NA: noradrenaline
NCDs: non-communicable diseases
NE: norepinephrine
NKCC: Na^+^/K^+^/2Cl^-^ co-transporter
NO: nitric oxide
NOAEL: no-observed-adverse-effect-level
NOEL: no-observed-effect level
Nrf2: nuclear factor-erythroid 2 related factor 2
NS: nephrotic syndrome
OS: oxidative stress
PDE: phophodiesterase
PE: phenylephrine
PG: prostaglandin
PI3-k/Akt: phosphotidylinositol 3-kinase (Protien kinase B)
PKC: protein kinase-C
PNS: parasympathetic nervous system
PPAR: peroxisome-proliferator-activated receptors
PT: prothrombine time
PVD: peripheral vascular disease
PVR: peripheral vascular resistance
RAAS: renin-angiotensin-aldosterone system
RBC: rRed blood cell
RCT: randomized control trial
RI: renal insufficiency
ROCC: receptor-operated calcium channel
ROS: reactive oxygen species
RSnH: hydropolysulfide
Rx: treatment
sGC: soluble guanylyl cyclase
SHR: spontaneously hypertensive rat
SM: smooth muscle
SNS: sympathetic nervous system
SV: stroke volume,
T2DM: Type 2 diabetes mellitus
T3: triiodothyronine
T4: thyroxine
TC: total cholesterol
TG: triglyceride
VCAM-1: vascular cell adhesion molecule-1
VD: vascular dysfunction
VDCC: voltage-dependent calcium channel
VF: vascular function
VGCC: voltage-gated calcium channel
VR: venous return
VSCC: voltage-sensitive calcium channel
VSMCs: vascular smooth muscle cells
WBC: white blood cell
WHO: World Health Organization
Zn: zinc
