# Supplementary material for: Medicinal plants administered to control hypertension in Ethiopia: ethnomedicine, pharmacology, nutraceutical, phytochemistry, toxicology, and policy perspectives
Source: Front Cardiovasc Med. 2025 Sep 5;12:1514911. doi: 10.3389/fcvm.2025.1514911 (PMC12446316; doi:10.3389/fcvm.2025.1514911)
Supplement: Supplementary file 1 [file Datasheet1.pdf]

Supplementary material 1: Medical plants used to manage hypertension by the local community in Ethiopia

S. No.	Scientific Name	Common Name in English	Vernacular Name	Family	Part/s Used	Method of Preparation and Application	Ref. No.
1	*Acanthospermum hispidum* DC.	Starbur	Sitar ber (Am) Armagusa (Ao)	Asteraceae	Leaf	The leaves crushed, boiled & one tea cup taken orally via drinking within 12 h difference for a week	[55]
2	*Achyranthes aspera* L.	Prickly chaff flower	Telenji (Am)	Amaranthaceae	Root, shoot tip	The root is sniffed through the nostrils	[56]
3	*Acokanthera schimperi* (A.DC.) Benth. & Hook.f. ex Schweinf**.**	Arrow Poison Tree	Kerero (Am)	Apocynaceae	Leaf	Not mentioned	[57]
4	*Ajuga integrifolia* Buch.-Ham.	Bracted Bugleweed	Tut Astil or Akorarach (Am), Armagusa (Ao), Anamuro (Sa)	Lamiaceae	Leaf, Root	Roots & leaves boiled with tea & a cup of solution is taken orally, OR, fresh leaf is pounded, decocted, & taken with tea or coffee via drinking	[57, **58**, 59, 60]
5	*Ajuga remota* Benth.		Akorarach (Am), Armagusa (Ao)	Lamiaceae	Leaf	The leaf is decocted or the squeezed leaf is made to juice & taken orally	[61]
6	*Allium cepa* L.	Red onion	Key shinkurt (Am)	Amaryllidaceae	Bulb, seed	The chopped bulb macerated in water, filtered & taken orally via drinking; Fresh seed is crushed & immersed in little water for 1 day and then filtrated by clean cloth and taken orally before food	[62, 63]
7	*Allium sativum* L.	Garlic	Nech shinkurt (Am), Tsaida shingurti (T), Tuma (Ka)	Amaryllidaceae	Bulb, seed	Frequent eating of the fresh bulb with injera, OR, eat the fresh bulb at the bed time, OR, mixed with *Allium porrum* L.,	[57, 64, 65]
8	*Aloe megalacantha* Baker subs. Megalacantha	Large-toothed Aloe	Eret (Am)	Asphodelaceae	Exudates	Powder of exudate in water solution is taken orally	[**66**]
9	*Aloe secundiflora* Engl.		Eret (Am)	Asphodelaceae	Not Mentioned	Not mentioned	[**67**]
10	*Apodytes dimidiata* E.Mey. ex Arn.	White pear	Cheleleqa, Donga (Am), Chalalaqa (Ao), Dongicho (Sa)	Metteniusaceae	Leaf	Decoction made with tea	[68]

11	*Artemisia absinthium* L.	Wormwood	Ariti (Am), Aguffa/natra (Ao) Naatiruwa (Dw)	Asteraceae	Leaf	Pounded & chewed, OR, liquid dosage form taken orally	[62, 65, 69]
12	*Artemisia annua* L.	Sweet wormwood		Asteraceae	Leaf	Fresh or dried leaf extract is consumed orally together with daily diet.	70
13	*Artemisia schimperi* Sch. Bip.ex Engl.	African wormwood	Harrittaa (Ao)	Asteraceae	Leaf	Fresh or dried leaf extract is consumed together with daily diet.	70
14	*Asparagus aethiopicus* L.	Asparagus fern	Yeset-kest (Am)	Asparagaceae	Root	Not mentioned	[71]
15	*Balanites aegyptiaca* (L.) Delile.	Desert date	Kudkuda, jemo, or bedeno (Am), Dhumuko(Hm)	Zygophyllaceae	Bark	Inside of the bark is peeled off, infusion is made with water, filtered & taken orally via drinking	[72]
16	*Bersama abyssinica* Fresen.	Winged bersama	Azamir (Am), Dobi warabechaa (Ao)	Francoaceae	Leaf, root	Dried/fresh sample crushed & mixed with honey to take orally once daily for 3 consecutive days	[56, 73, 74]
17	*Cadaba farinosa* Forssk.		Dhela (Hm)	Capparaceae	Root	Roots are chopped, boiled with meat soup & taken orally via drinking	[72]
18	*Calpurnea aurea* (Aiton) Benth.	Cape Laburnum	Digitta (Am), Cekkatta (Sa), Cheka (Ao)	Fabaceae	Leaf, Seed	Seed is crushed & sniffed through the nostrils, OR, dried, crushed & swallowed by touching nose, OR, the leaf is decocted	[61, 75, 76, 77]
19	*Carica papaya* L.	Papaya	Papaya (Am), Paappaayyaa (Ao), Paappaye (Sa)	Caricaceae	Leaf, seed	Dried, pounded, & boiled fresh leaf mixed with sugar is taken orally for some time (often mixed with fresh leaves of *Ajuga integrifolia*), OR, seed is ground, blended with honey & taken with empty stomach	[56, 60]
20	*Catha edulis* (Vahl) Endl.	Bushman's tea	Chat (Am) Chatyae (Ar)	Celastraceae	Leaf	Fresh leaves are chewed with or without sugar	[57, 78]
21	*Centaurium pulchellum* (Sw.) hayek ex Hand.-Mazz., Stadlm., Janch. & Faltis	Lesser centaury		Gentianaceae	Root	The root is boiled in water and taken orally	[79]
22	*Cinnamomum verum* J. Presl	Cinnamon	Kerefa (Am)	Lauraceae	Root	Not mentioned	[57]

23	*Citrullus lanatus* (Thunb.) Matsum. & Nakai	Watermelon	Habhab (Am)	Cucurbitaceae	Fruit	Squeezing it & taking the juice orally	[80]
24	*Citrus aurantium* L.	Bitter orange	Komtatie (Am), Qomxaaxxee (Ao)	Rutaceae	Fruit	Drink the juice or suck the fruit content when suffered by HTN	[81, 82]
25	*Citrus aurantiifolia* (Christm.) Swingle	Lime	Lomi (Am), Tutto (Ar)	Rutaceae	Fruit	Fruit & fruit juice are taken orally	[76, 78]
26	*Citrus limon* (L.) Osbeck	Lemon	Lomi (Am)	Rutaceae	Fruit	Fresh juice is mixed with tomato & taken orally	[63, 83, 84]
27	*Citrus medica* L.	Citron	Tiringo (Am)	Rutaceae	Fruit	Eaten & taken orally	[83, 85]
28	*Coccinia grandis* (L.) Voigt	Scarlet gourd	Werkbemeda (Am)	Cucurbitaceae	Root	Not mentioned	[57]
29	*Coriandrum sativum* L.	Coriander	Dimbilal (Am)	Apiaceae	Seed, fruit	Not mentioned	[57]
30	*Crepis rueppellii* Sch. Bip.		Yemidir gusmt (Am)	Asteraceae	Root	Root is boiled in water & taken as tea at bed time	[86]
31	*Crinum abyssinicum* Hochst. ex A. Rich.		Yejib shinkurt (Am)	Amaryllidaceae	Shoot tip	Fresh form is squeezed & mixed the liquid with water, & taken orally via drinking	[76]
32	*Croton macrostachyus* Hochst. ex Delile	Broad-leaved croton	Bisana (Am)	Euphorbiaceae	Leaf, latex	Crushed, squeezed & taken via nasal route	[87]
33	*Cucumis ficifolius* A. Rich.		Yemidir enbuay (Am) Holoto (Ao)	Cucurbitaceae	Root	Fresh or dried finger strip size of the root is pounded, soaked in water, decanted and then mixed with honey and drunk	**70**
34	*Cymbopogon citratus* (DC) Stapf.	Lemongrass	Tej-sar (Am) Hitichuta (Ka)	Poaceae	Leaf	Not mentioned	[65]
35	*Datura stramonium* L.	Jimsonweed	Atefaris or Astenager (Am) Asangra (Ao)	Solanaceae	Leaf	The dried leaf is smoked through intranasal route	[61]
36	*Dorstenia barnimiana* Schweinf.		Work bemeda (Am)	Moraceae	Root	Not mentioned	[**88**]
37	*Dovyalis abyssinica* (A. rich.) Warb.	Abyssinian Gooseberry	Yehabesha Koshim (Am)	Salicaceae	Leaf, root, stem, stem tuber	Root & stem tuber are smashed, mixed & taken with alcohol via drinking, OR, the leaf become boiled & the liquid is	[57, 87, 89]

						taken orally, OR, fresh bark chopped, infusion is made, mixed with honey & taken orally via drinking	
38	*Embelia schimperi* Vatke		Enqoqo (Am) Anku (Ao) Dupho/C'omat'o (Sk)	Myrsinaceae	Fruit	Fresh fruit of *Embelia schimperi* together with whole plant of *Ruta chalepensis* and root of *Rumex abyssinicus* are pounded together and the water extract is drunk with honey or any appropriate drink.	**70**
39	*Ferula communis* L.	Giant Fennel	Doge (Am)	Apiaceae	Leaf	Crush leaves added to boiled tea & taken orally via drinking	[89]
40	*Foeniculum vulgare* Mill.	Fennel	Ensilal (Am)	Apiaceae	Leaf	Fresh form is boiled & taken orally via drinking	[57, 60, 76]
41	*Hagenia abyssinica* (Bruce) J.F.Gmel.	African redwood	Kosso (Am)	Rosaceae	Leaf, fruit, flower	Fresh fruit & leaf is boiled with little water & mixed with alcohol given orally; Fresh flower & fruit soaked with water is given orally	[75, 83, **90**]
42	*Hibiscus sabdariffa* L.	Roselle (sour tea)	Kedkedie (Am)	Malvaceae	Flower	Not described	[56, 91]
43	*Hordeum vulgare* L.		Tikur Gebs (Am)	Poaceae	Seed	Taken as a fermented barley drink. Germinated barley & Mashilla (Sorghum sp.) are baked together like bread. This is broken up & fermented together with beqil (malt starter), brewed & distilled & taken orally via drinking by a shot glass	[92]
44	*Jatropha curcas* L.	Physic nut	Ayderke (Am)	Euphorbiaceae	Seed	Honey paste of the seed powder taken orally	[93]
45	*Leucaena leucocephala* (Lam.) de Wit	White leadtree	Lukina (Am), Lalombaka (Hm)	Fabaceae	Whole plant	Different parts mixed, crushed, & macerated/infused in water, filtered & mixed with honey & milk & then taken orally via drinking	[72]
46	*Linum usitatissimum* L.	Flaxseed	Telba (Am)	Linaceae	Seed, root	Not described	[57]
47	*Lupinus albus* L.	White lupin	Gibto (Am)	Fabaceae	Seed, fruit	The dried seed is roasted & one spoon of the flour mixed with one glass of water then drunk during pain, OR, roast	[57, 73, 87, 94, 95]

						the seed, soak in water, & mix with pepper & take orally twice daily, OR, pounding & cooking of seed & taken via the nose, OR, soaking seed with water for 3–5 days, decanting the water, & eating &/or preparing in the form of alcohol & drunk	
48	*Lupinus termis* Forssk.	White lupin	Gibto (Am)	Fabaceae	Seed	Not mentioned	[57]
49	*Melia azedarach* L.	Chinaberry tree	Mimi-zaf (Am) Fayo (Ao)	Meliaceae	Leaf	Leaves are pounded & the juice is taken orally	[96]
50	*Mentha* × *piperita* L.	Peppermint	Nana (Am)	Lamiaceae	Leaf	The juice of the squeezed leaf is taken orally	[61, 75, 97]
51	*Mentha spicata* L.	Spearmint	Naanna (Ka)	Lamiaceae	Leaf	Mixed with *Carissa spinarum* & *Citrus aurantifolia*	[65]
52	*Meriandra dianthera* (Roth ex Roem. & Schult.) Briq.	Bengal sage	Mesaguh (T)	Lamiaceae	Leaf	Crushed, filtered & drunk the fluid	[84, 98]
53	*Moringa oleifera* Lam.	Drumstick tree	Sheferaw (Am)	Moringaceae	Leaf	Dried, crush, & taken orally	[63]
54	*Moringa stenopetala* (Baker f.) cufod.	African moringa or cabbage tree	Sheferaw (Am), Kelanqi (Hm), Shifera (Sa), Halako (Ha) Halakuwa (Dw)	Moringaceae	Leaf, seed, fruit	Drying , pounding, & filtering it after adding water, OR, squeeze fresh leaf then drink, OR, crush then boil, filter, & drink, OR, fresh leaves are boiled, allowed to cool, & the filtrate is drink, OR, dried, make as a tea & drinking, OR, fresh form is boil with *Allium cepa* & *Capsicum annuuam*, add oil & take orally	[57, 69, 72, 75, 76, 80, 99, 100, 101]
55	*Nigella sativa* L.	Black cumin (Black seed)	Tikurazimud (Am)	Ranunculaceae	Seed	Not mentioned	[57]
56	*Ocimum lamiifolium* Hochst.		Damakase (Am)	Lamiaceae	Leaf	Fresh leaves are pounded & juice is prepared, and then taken orally three times a day for three days	[57, 75, 97]
57	*Ocimum urticifolium* Roth		Demakase (Am)	Lamiaceae	Leaf	Inhaled (oral & nasal)	[80]

58	*Otostegia integrifolia* Benth.	Abyssinian rose	Tinjute (Am) Chendog (T)	Lamiaceae	Leaf, stem	Leaves are boiled in water and a cup of solution is taken every morning until recovery	[57, 84]
59	*Passiflora edulis* Sims	Passion fruit	Hoophe (Sa)	Passifloraceae	Fruit	Two ripe fruits are eaten every morning for about 6 months	[60, 68]
60	*Persea americana* Mill.	Avocado	Avocado (Am) Abukato (Sa)	Lauraceae	Fruit	Dried, crushed, powdered, mixed with coffee & drunk	[75, 102]
61	*Phragmanthera macrosolen* (Steud. ex A. Rich.) M.G.Gilbert		Teketila (Am)	Loranthaceae	Leaf	Pounding or crushing, & drinking	[87]
62	*Phragmanthera regularis* (Steud. ex Sprague) M.G.Gilbert		Teketila (Am)	Loranthaceae	Leaf	Pounding or crushing, drinking	[87]
63	*Plumbago zeylanica* L.	Ceylon leadwort	Amera (Am)	Plumbaginaceae	Root	Not mentioned	[57]
64	*Premna schimperi* Engl.		Chocho (Am) Uregessa (Sh)	Lamiaceae	Leaf	Not mentioned	[64]
65	*Rhamnus prinoides* L'Hér.	Dogwood	Gesho (Am)	Rhamnaceae	Root & Leaf	Not mentioned	[100]
66	*Rosa abyssinica* R.Br. ex Lindl.		Kega (Am)	Rosaceae	Fruit	Powdered fruits are mixed with water & drunk, OR, the fruit is boiled & taken orally	[87, **103**]
67	*Rosmarinus officinalis* L.	Rosemary	Sigametebesha (Am)	Lamiaceae	Leaf	Fresh form is crushed, boiled & taken as a tea	[94, 101]
68	*Rubus apetalus* Poir.		Enjori (Am)	Rosaceae	Stem	Eating	[87]
69	*Rumex abyssinicus* Jacq.	Spinach rhubarb	Mekemeko (Am)	Polygonaceae	Root	Fresh/dry root is boiled with water & taken with milk, OR, dried root is crushed, mixed with water & add sugar, boil & then drink, OR, crush root, mix with bulbs of *A. sativum*, & then add the mixture into boiled water & drink the hot decoction in a cup, OR, fresh root juice is given to drink with coffee	[57, 73, 75, 77, 81, 83, 84, 89, 92, 104]

70	*Rumex nepalensis* Spreng.	Nepal Dock	Tult (Am)	Polygonaceae	Leaf, root	Fresh leaves are boiled & drunk daily, OR, about the size of fingertip of root is chewed & the juice is taken orally	[75, 78, 94]
71	*Ruta chalepensis* L.	Fringed rue	Tenadam (Am)	Rutaceae	Leaf, fruit	Not mentioned	[57, 73]
72	*Salvia tiliifolia* Vahl	Lindenleaf sage	Aqorarach (Am)	Lamiaceae	Leaf	Fresh leaf juice is mixed with little water & taken orally	[83]
73	*Satureja punctata* R.Br. ex Briq.		Lomishet (Am)	Lamiaceae	Leaf	Prepared as tea & taken orally as needed	[56, 94]
74	*Schinus molle* L.	Pepper tree	Qundo berbere (Am)<br>Tselim berbere (T)	Anacardiaceae	Stem	The stem is chewed	[84]
75	*Solanum nigrum* L.	Black nightshade	Awut (Am)<br>Tut'naye (Sa)	Solanaceae	Leaf	Leaves boiled thoroughly & eaten	[85]
76	*Spinacia oleracea* L.	Spinach	Kel (Am)	Amaranthaceae	Leaf	Eat frequently	[56]
77	*Syzygium guineense* (Willd.) DC.	Water berry	Dokma (Am)	Myrtaceae	Leaf	Not mentioned	[105]
78	*Tamarindus indica* L.	Tamarind	Roka (Am)<br>Humer (T)	Fabaceae	Fruit	Not mentioned	[106]
79	*Thymus schimperi* Ronniger		Tossign (Am)<br>Xosingii (Ao)<br>Tesni/Thasne (T)<br>Zazanchut (Ka)<br>Oyssamaataa/Zinbbanu wa (Dw)	Lamiaceae	Leaf, stem, root, Whole plant	Fresh form of leaf is crushed, squeezed & the fluid is taken, OR, Taken in tea form once daily for 6 months, OR, crushed normal (dry) leaf is boiled & taken in the form of tea with a tea glass, OR, dried stem & leaf powder boiled with tea & taken orally, OR, fresh leaf juice is given with tea, OR, root dried, powdered, & drink with tea using sugar as additive	[57, 59, 65, 69, 75, 87, 92, 99, 101, 104, 107, 108, 109]
80	*Thymus serrulatus* Hochst. ex Benth.		Tossign (Am)	Lamiaceae	Leaf	Fresh leaves are soaked with warm water, filterate is drunk	[109, 110]
81	*Trigonella foenum-graecum* L.	Fenugreek	Abish (Am)	Fabaceae	Seed	Extract of the dried & powdered seed is taken orally	[57, 75]

82	*Verbascum sinaiticum* Benth.		Daba Keded (Am)	Scrophulariaceae	Root	Chewing	[87]
83	*Vernonia amygdalina* Delile	Bitter leaf	Girawa (Am)	Asteraceae	Leaf	Not mentioned	[57]
84	*Zehneria scabra* Sond.		Haregresa (Am)	Cucurbitaceae	Leaf	Not mentioned	[57]
85	*Zingiber officinale* Roscoe	Common ginger	Zinjibil (Am)<br>Gengible (T)	Zingiberaceae	Rhizome	The rhizome is chewed	[57, 84]

Am: Amharic, Ao: Afaan Oromo, T: Tigregna, Sa: Sidamu-afoo, Ka: Kambattissa, Ar: Amaro, Ha: Halaba, Sh: Shinasha, Dw: Dawero, Sk: Shekinano
