# Supplementary material for: Medicinal plants administered to control hypertension in Ethiopia: ethnomedicine, pharmacology, nutraceutical, phytochemistry, toxicology, and policy perspectives
Source: Front Cardiovasc Med. 2025 Sep 5;12:1514911. doi: 10.3389/fcvm.2025.1514911 (PMC12446316; doi:10.3389/fcvm.2025.1514911)
Supplement: Supplementary file 2 [file Datasheet2.pdf]

Supplementary material 2: Additional (other) local and traditional uses of Medical plants used to control hypertension in Ethiopia

S. No.	Botanical Name	Illnesses/Symptoms Claimed to Be Treated Traditionally	Ref. No.
1	*Acanthospermum hispidum* DC.	Tetanus, itching skin, joint and shoulder pain	[**126, 127, 128**]
2	*Achyranthes aspera* L.	Stomachache, abdominal cramp in woman after birth, Rhesus factor incompatibility in pregnancy, nose bleeding, stabbing pain, uvulitis, tonsillitis, swelling, wound, bleeding after skin cut, bleeding after delivery, blood clotting, snake bite, gonorrhea, excessive menstruation, eye problem/infection, bone fracture, paralysis, tape worm, retained placenta, arthritis, herpes zoster, anthrax, hemorrhoids, urinary retention, fever, eye dusts, ear mites, skin rash, sudden sickness, conjunctivitis, & impetigo	[59, 61, 73, **74**, 76, 77, 78, 81, 83, 84, 89, 95, 98, 99, 10, 108]
3	*Acokanthera schimperi* (A.DC.) Benth. & Hook.f. ex Schweinf.	Scabies, stabbing pain, headache, arrow poison, hemorrhoids, gonorrhea, syphilis , wound, intestinal parasite, evil eye, hepatitis, spider poison, amebiasis, psychiatric disease, insecticide, snake bite, & herpes virus	[55, 60, 61, 64, 73, 77, 81, 83, 87, 92, 106]
4	*Ajuga integrifolia* Buch.-Ham.	Stomachache, cold, gout, evil eye, ascariasis, tape worm, rheumatism, anthrax, Tinea capitis, tonsillitis, malaria, pneumonia, swelling, & epilepsy	[59, 65, 78, 98, 99, 101, 104]
5	*Ajuga remota* Benth.	Common cold, pneumonia, bronchitis, abdominal pain, colic, & diabetes mellitus	[**129**]
6	*Allium cepa* L.	Poisoning, typhoid fever, urethra problem, earache, Tinea versicolor, stomach complaints, headache, asthma, malaria, taeniasis, pneumonia, common cold, & whooping cough	[59, 69, 73, 80, 87, 89, 92, 100, 104]
7	*Allium sativum* L.	Stomachache, ascariasis, general malaise, flu or common cold, toothache, malaria, tonsillitis, diabetes mellitus, athletes foot, gonorrhea, elephantiasis, diarrhea, rheumatism, snake bite & snake repellant, typhoid fever, jaundice, coccoides, hemorrhoids, sudden illness, asthma, wound , headache, chest pain, cough, amebiasis, pneumonia, evil eye, lung cancer/ tuberculosis, gastritis, ring worm, eye infection, paralysis, itching/scabies, rabies, sun-stroke, & heartburn	[55, 59, 60, 61, 63, 65, 69, 73, 76, 78, 80, 84, 89, 98, 101, 108]
8	*Aloe megalacantha* Baker subs. Megalacantha	Skin infection, eye infection, stomach ulcer, anthrax, colon cleaner, leg & back pain, malaria, diabetes mellitus, dandruff, impotence, wound, amebiasis, ascariasis, abdominal pain, evil eye, snake bite, & urinary retention	[66, **67**, 84, 98]
9	*Aloe secundiflora* Engl.	Skin infections, wound healing, rheumatism, ectoparasite, malaria, diarrhea, arthritis, backache, cold, flu, cough, chest problems, eye infections, kidney problems, liver problems, oral thrush, bile problems, ringworm, stiff muscles, stomachache, diaphragm problems, swelling of legs, tuberculosis, typhoid fever, ulcer, boils, & vomiting	[66, **67**]

10	*Apodytes dimidiata* E.Mey. ex Arn.	Stomachache, growth retardation, & urinary retention	[60, 83]
11	*Artemisia absinthium* L.	Sour throat, dandruff, cough, fever, stomachache, infant growth, severe abdominal cramp, uvula infection, evil eye, tonsillitis, & syphilis	[59, 65, 68, 69, 80, 99, **103**]
12	*Artemisia annua* L.	Cardiac problems, renal problems, malaria, & breast cancer	[**70**, 130]
13	*Artemisia schimperi* i Sch. Bip.ex Engl.	Cardiac problems, renal problems, stomachache, & bleeding	[**70**, 131]
14	*Balanites aegyptiaca* (L.) Delile.	Rabies, headache, abdominal pain, toothache, rheumatism, anthelmintic, purgative, vermifuge, emetic & in the Rx of boils, leucoderma, herpes, malaria, wounds, syphilis, cold & liver, Tinea nigra, bloody diarrhea, dandruff, intestinal constipation, & tuberculosis	[55, 64, 76, 79, 80, 99, 100, 106]
15	*Bersama abyssinica* Fresen.	Mouth inflammation, cancer, eczema/skin disease, snake bite , devil sickness, ascariasis, stomachache, rabies, & wound	[55, 60, 62, 65, 73, 76, 99, 100, 104]
16	*Cadaba farinosa* Forssk.	Malaria, eye sickness, lung infection, flue, swelling on body, head injury, breast cancer, dyspepsia, typhoid fever, angina pectoris, extra pulmonary tuberculosis, anthrax, arthritis, impotence, infant sickness, mastitis & contagious agalactia, bovine pastuerollosis, & gastrointestinal illness and intestinal parasites	[72, 132, 133]
17	*Calpurnea aurea* (Aiton) Benth.	Scabies, diabetes mellitus, body lice, malaria, diarrhea, vomiting, impotency, jaundice, ascariasis, amebiasis, giardiasis, rabies, bilharziasis, erthroblastosis, expel foreign things from eye, prolonged embryo in uterus, wound, skin infection, abdominal pain, syphilis, gonorrhea, mental disorder, snake bite, herpes zoster, hemorrhoids, Tinea capitis, excessive bleeding after birth, swelling, tuberculosis, & insecticide	[59, 61, 63, 69, 76, 77, 81, 83, 84, 86, 87, 98, 99, 106]
18	*Carica papaya* L.	Gastritis, malaria, gastro-esophageal reflux diseases, amebiasis, intestinal worms**,** cough**,** stomachache, abortion, trypanosomiasis, liver infection, jardiasis, anemia, swelling on the skin, wound, diabetes mellitus, diarrhea, ring worm, & herpes virus	[60, 61, 63, 65, 68, 69, 76, 80, 81, 82, 87, 95, 98, 106]
19	*Catha edulis* (Vahl) Endl.	Ear infection, stomach disorder, anthrax, asthma, stomachache, body odour, tonsillitis, gonorrhea, cough, malaria, psychiatric disease, intestinal parasite, & urinary retention	[59, 69, 73, 76, 82, 87, 92, 99, 104, 108]
20	*Centaurium pulchellum* (Sw.) hayek ex Hand.-Mazz., Stadlm., Janch. & Faltis	Gastric & abdominal pain, renal colic, diabetes mellitus, rheumatic pains and for the elimination of stones from the kidney and urethera; healing agent for wounds in ointments for sciatica	[**79**]
21	*Cinnamomum verum* J. Presl	Taeniasis, asthma, common cold, & fever	[92, 134]
22	*Citrullus lanatus* (Thunb.) Matsum. & Nakai	Stomach complaints, mastitis and contagious agalactia	[80, 132]
23	*Citrus aurantium* L.	Amebiasis, wound, dandruff, & swelling	[63, 101]

24	*Citrus aurantiifolia* (Christm.) Swingle	Abdominal pain, common cold, cough, anti-emetic, anthrax, wound, Tinea versicolor, cancer, tetanus, constipation, skin cutting, gonorrhea, scabies, poison, snake bite, Tinea capitis, acne, & indigestion	[59, 62, 65, 68, 69, 73, 76, 75, 87, 94, 97, 98, 99, 100]
25	*Citrus limon* (L.) Osbeck	Amebic dysentery (amebiasis), liver disease, stomachache, gum bleeding, skin burn, coccidiosis, hemorrhoids, cough, tetanus, stop vomiting, asthma, evil spirit, & tuberculosis	[64, 73, 80, 82, 83, 84, 85, 89, 101, 106]
26	*Citrus medica* L.	Pain attack, loss of appetite, gum bleeding, & indigestion	[63, 73, 100]
27	*Coccinia grandis* (L.) Voigt	Malaria & liver disease	[**135**]
28	*Coriandrum sativum* L.	Abdominal pain, diarrhea, loss of appetite, ascariasis, & malaria	[65, 69, 76, 77, 101]
29	*Crepis rueppellii* Sch. Bip.	Snake bite, liver infection, diarrhea, gonorrhea, stomachache, vomiting, anthrax, dysentery with blood, & wart	[69, 81, 86]
30	*Crinum abyssinicum* Hochst. ex A. Rich.	Swelling of stomach, diabetes mellitus, & cancer	[55, 76, 86]
31	*Croton macrostachyus* Hochst. ex Delile	Black leg (Tinea nigra), ring worm (such as dandruff), tapeworm, febrile illness, malaria, retained placenta, leprosy, wound, stop bleeding, dermal swelling, vitiligo, obesity, snake bite, gonorrhea, lymphadenitis, severe abdominal cramp, ascariasis, liver infection, stomachache, diarrhea, headache, rheumatism, evil eye, jaundice, eye disease, eczema/ atopic dermatitis/skin rash, hemorrhoid, skin cancer, rabies, intestinal worms, bloating, paralyzed leg, bloating, mosquito repellant, Tinea versicolor, Tinea corporis, Tinea capitis, epistaxis (nose bleeding), herpes zoster, shivering/inflammation, tonsillitis, asthma, spleenomegally, & epilepsy	[55, 59, 60, 61, 63, 64, 65, 68, 69, 73, **74**, 76, 77, 80, 81, 82, 86, 87, 92, 94, 98, 100, 101, 106]
32	*Cucumis ficifolius* A. Rich.	Sudden stomachache, dry cough, diarrhea, wound, chest pain, hemorrhoid, & rabies	[**136**, 137, **138**, **139**]
33	*Cymbopogon citratus* (DC) Stapf.	Cough, GIT disorder, boost immunity of breastfeeding infants, abortifacient, malaria, evil eye, stomachache, & headache	[59, 60, 65, 69, 73, 82, 100]
34	*Datura stramonium* L.	Rabies, toothache (odontalgia), asthma, diabetes mellitus, hepatitis, baldness, ring worm (such as dandruff), gum problem, deafness, tumor, wound, hemorrhoid, weight loss in children, expel foreign things from eye, scabies, nasal bleeding, anal prolepses, rheumatic pain, hallucinogenic, herpes zoster, & anthrax	[55, 61, 63, 65, 69, 73, 76, 77, 81, 82, 83, 87, 92, 98]
35	*Dorstenia barnimiana* Schweinf.	Hemorroid, cancer, ejeseb, malaria, evil sprit, rabies, syphilis, unhealthy weight loss, dysentery, fever with rush on the body, hepatitis, & leprosy	[**74**, 86, 89]
36	*Dovyalis abyssinica* (A. rich.) Warb.	Tumor, joint pain, tonsillitis, fibril illness, boil, hemorrhoid, cancer, abdominal pain, asthma, ascariasis, gum bleeding, amebiasis, tape worm, toothache	[60, 63, 65, 73, 81, 83, 87, 89, 98, 101]
37	*Embelia schimperi* Vatke	Tapeworm, hanger, glandular, gonorrhea, jaundice, all stomach disease and parasite, & asthma	[**70**, 134, **136**, 137]
38	*Ferula communis* L.	Cough, wart on hand, impotency, gonorrhea, increase sexual needs, evil sprit, lung cancer, erthroblastosis, night blindness, unable to urinate, intestine pain	[59, 73, **74**, 81, 87, 89, 108]

39	*Foeniculum vulgare* Mill.	Stomachache, urinary retention, diuretic, laxative, clean stomach, rheumatism, intestinal problem, tonsillitis, gonorrhea, wart, diabetes mellitus, cough, asthma, bloating, headache, toothache, & kidney infection	[55, 59, 61, 68, 69, 73, 76, 81, 83]
40	*Hagenia abyssinica* (Bruce) J.F.Gmel.	Swelling of stomach, tapeworm (taeniasis), allergic dermolitia, wound, stomach distention, & malaria	[55, 60, 61, 73, 80, 108]
41	*Hordeum vulgare* L.	Broken bones, worn out tissues, diarrhea, cold, gastric ulcer, & trypanosomiasis	[55, 63, 65, 83, 86]
42	*Jatropha curcas* L.	Tape worm, clotting blood, wound, & rabies	[69, 77]
43	*Leucaena leucocephala* (Lam.) de Wit	Intestinal parasite, irregular menstruation, & loss of appetite	[72]
44	*Linum usitatissimum* L.	Wound, breast pain, uterine pain, stomachache, constipation, amebiasis/giardiasis, gastritis, anemia, chest/back pain, diarrhea, eye disease, intestinal wound, demulcent, & stomach ulcer	[62, 63, 65, 68, 69, 83, 87, 89, 92, 100]
45	*Melia azedarach* L.	Taeniasis, abortion, malaria, intestinal worms, toothache, dandruff, anti-insecticide, wound, & abdominal pain	[59, 63, 65, 76, 81, 98, 100]
46	*Mentha × piperita* L.	Common colds, & diarrhea	[61, 81]
47	*Mentha spicata* L.	Cough, cold, & headache	[59, 73]
48	*Meriandra dianthera* (Roth ex Roem. & Schult.) Briq.	Trachoma, diarrhea, malaria	[84, 99, 118]
49	*Moringa oleifera* Lam.	Amebiasis	[118]
50	*Moringa stenopetala* (Baker f.) cufod.	Trypanosomiasis, malaria, stomachache, anthrax, diarrhea, vomiting, rheumatism, abdominal colic, kidney infection, cold, & diabetes mellitus	[69, 72, 76]
51	*Nigella sativa* L.	Leprosy, streptothricosis, headache, abdominal pain or stomachache, cough, skin fungus, asthma, runny rose, common cold, intestinal parasite, abdominal colic, diarrhea, respiratory inflammation, pneumonia, & peptic ulcer disease	[61, 63, 64, 65, 76, 77, 85, 97, 100, 101, 106]
52	*Ocimum lamiifolium* Hochst.	General malaise, acute viral infection, cough, headache, sun-strike, febrile illness, eye disease, common cold/influenza, diarrhea, poor appetite, flatulence, skin diseases, evil eye, gastritis, wound, diabetes, tonsillitis, fungal infection, & unidentified GIT disorder	[59, 60, 61, 63, 68, 69, 72, 76, 78, 92, 104]
53	*Ocimum urticifolium* Roth	Oral inflammation, fever, abdominal complaints, allergy, headache, common cold, & febrile illness	[55, 65, 68, 80, 83]
54	*Otostegia integrifolia* Benth.	Febrile illness, evil eye, acute mountain sickness, malaria, epidemic & common cold, Coccidia, stomachache, lung diseases, vomiting, evil spirit, ascariasis, insecticide, nausea, diarrhea, & dysentery	[59, 64, 73, 77, 81, 83, 87, 98, 106, 108]
55	*Passiflora edulis* Sims	Pneumonia	[69]

56	*Persea americana* Mill.	Dandruff, diarrhea, rheumatism, Kidney infection, hemorrhoid, cease bleeding, & diabetes mellitus	[68, 69, 81, 85, 87]
57	*Phragmanthera macrosolen* (Steud. ex A. Rich.) M.G.Gilbert	Swelling & general malaise	[76, **140**]
58	*Phragmanthera regularis* (Steud. ex Sprague) M.G.Gilbert	Intestinal infection	[**141**]
59	*Plumbago zeylanica* L.	Gonorrhea, irritative swell in the skin, abdominal pain, arthritis, neck pain, toothache, chronic cough, asthma, impotence, gland tuberculosis, malaria, bone tuberculosis, heart disease, hemorrhoid, wound, scorpion poison, stomach tumor, tonsillitis, snake bite, anthrax, teeth infection, & diarrhea	[61, 64, **74**, 77, 81, **90**, 99, 106]
60	*Premna schimperi* Engl.	Tissue cancer, eye disease, ascariasis, severe abdominal pain, malaria, Tinea pedis, hemorrhoids, wound, inflammation of skin, toothache, & diarrhea	[55, 59, 65, 69, 73, 77, 81, 100]
61	*Rhamnus prinoides* L'Hér.	Tonsillitis, sexually transmitted diseases (such as gonorrhea), wound, scabies, fever in children, liver problem/hepatitis, skin diseases/itching/skin rash, uvulitis, snake bite, herpes, ring worm, swollen lymph nodes, dysentery, Tinea capitis, & anthrax	[55, 60, 62, 63, 64, 69, 72, 73, 78, 81, 83, 85, 92, 95, 98, 106]
62	*Rosa abyssinica* R.Br. ex Lindl.	Evil sprit, stomachache, toothache, ascariasis, tension/dizziness, gastritis, tape worm, & malaria	[59, 68, 73, 81, 89, **90**, 100, **103**]
63	*Rosmarinus officinalis* L.	Headache, stomachache, & toothache	[59, 76, 83, 94, 101]
64	*Rubus apetalus* Poir.	Tuberculosis, gonorrhea, & gastritis	[60, **90**]
65	*Rumex abyssinicus* Jacq.	Eye bruise, blackleg, scabies, jaundice, gonorrhea, lung tuberculosis, hepatitis/liver infection, diabetes mellitus, nephropathy, all intestinal parasites, tinea versicolor, amebiasis, itching skin, vitiligo, common cold, pharyngitis, goiter, diarrhea, headache, expel delayed embryo in the uterus, teeth infections, & stomach pain	[59, 60, 61, 65, 69, 73, 76, 77, 80, 83, 92, 98, 99, 106]
66	*Rumex nepalensis* Spreng.	Colic, blackleg, stabbing pain, urinary retention, Tinea capitis/dandruff, tonsillitis, anorexia, gastritis, hemorrhoid, nephropathy, stomachache, severe abdominal/stomach pain, acute mountain sickness, hemorrhage, arthritis, abortion, to induce uterine contraction, wound, amebic dysentery, diarrhea, anthrax, leshimaniasis, excess bleeding during giving birth, ring worm, abdominal impelling, abdominal swelling, evil eye, retained placenta, wart, Rhesus factor problem in pregnancy, fire burn, & malaria	[59, 61, 65, 69, 73, **74**, 77, 78, 81, 86, 87, **90**, 92, 98, 104]
67	*Ruta chalepensis* L.	Stomachache, cold disease, detoxify poison, painkiller for various ailments, ear infection, common cold, malaria, evil eye, fever, severe abdominal cramp, trypanosomiasis, diabetes mellitus, gonorrhea, ascariasis, asthma, stroke, swelling, vomiting, colic in baby, cough, & tonsillitis	[55, 60, 61, 63, 64, 65, 69, 73, 76, 80, 87, 92, 99, **103**]
68	*Salvia tiliifolia* Vahl	Tonsillitis & febrile illness	[83]
69	*Satureja punctata* R.Br. ex Briq.	Febrile illness, fever, leech infection, & cold	[83, 94]

70	*Schinus molle* L.	Wound on rectal area, jaundices, sore throat, abdominal pain, tonsillitis, toothache, malaria, cough, tuberculosis, diarrhea, & herpes virus	[59, 63, 68, 73, 77, 81, 89, 98, 106]
71	*Solanum nigrum* L.	Malaria (*P. vivax*), cancerous sores, leucoderma & wounds, considered tonic for virility in men & for dysmenorrhea in females, dysentery, sore throat, spider poison, diarrhea, hemorrhoid, stomachache, difficult urination**,** kidney disease, scabies (itching), dactylitis, herpes zoster, liver problem, & gastritis	[60, **79**, 81, 85, 87, 99, 108]
72	*Spinacia oleracea* L.	COVID-19	[**142**]
73	*Syzygium guineense* (Willd.) DC.	Obesity, diarrhea, stomachache, toothache, colic pain, kidney infection, liver cirrhosis, tonsillitis, hemorrhoid, malaria, internal worms, snake bite, gonorrhea, leprosy, & vomiting	[60, 61, 68, 69, 78, **90**, 100]
74	*Tamarindus indica* L.	Vermifuge, dermal infection, ascariasis, stomachache, diarrhea, spleenomegally, & abdominal problems	[61, 64, 69, **79**, 100, 106]
75	*Thymus schimperi* Ronniger	Toothache, tonsillitis, lung tuberculosis, vomiting, diabetes mellitus, whooping cough, abdominal pain, & cough	[69, 73, 83, 92, 98, **103**, 104, 107, 108, 109]
76	*Thymus serrulatus* Hochst. ex Benth.	Liver ailments, renal disease, & Tinea capitis	[61, 109, 110]
77	*Trigonella foenum-graecum* L.	Varicose vein, diarrhea, nephropathy, abdominal complaints including abdominal colic, fattening, toothache, melasma, gastritis, evil eye, broken leg, wound, cough, demulcent, swelling, typhoid fever, pneumonia, peptic ulcer disease, spinal pain, & tonsillitis	[63, 64, 65, 69, 73, 80, 87, 92, 97, 98, 101, 104]
78	*Verbascum sinaiticum* Benth.	Nightmare, blackleg, eye disease, allergic dermolitia, retained placenta, asthma, mental stress/psychiatric disease, stomachache, diarrhea, evil sprit, evil eye, snake bite, leech infection, swelling, lymphadenitis, nose bleeding (epistaxis), loss of weight, poisoning , skin disease, gastritis, fire burn, tonsillitis, toothache, external wound, hemorrhoids, anthrax, & febrile illness	[59, 64, 73, 76, 80, 81, 83, 87, 89, **90,** 92, 95, 98, 99]
79	*Vernonia amygdalina* Delile	Bloating, urine retention, abdominal pain/stomachache, malaria**,** helminthiases (such as ascariasis), watery diarrhea, alcohol addicts, typhoid fever, rheumatism, cold, retained placenta, wound, bleeding, fattening, toothache, eczema, dandruff, impotency, worms, abortion, tonsillitis, amebiasis, giardiasis, liver disease, gout, & shivering	[55, 59, 60, 61, 65, 69, 76, 81, 85, 86, **90**, 92, 99, 100]
80	*Zehneria scabra* Sond.	Deformed lips, urinary retention, diarrhea, headache, fever, kidney infection, liver cirrhosis, jaundice, rabies, gonorrhea, intestinal parasites, malaria, wart, eye disease (conjunctivitis, trachoma), to induce uterine contraction, anemia, swelling, wound, febrile illness, ring worm (such as dandruff), Tinea capitis, stomachache, sun-strike, abdominal colic, & cough	[59, 61, 69, 73, 73, 77, 81, 83, 86, 87, 92, 95, 97, 107, 108]

81	*Zingiber officinale* Roscoe	Tonsillitis, pain management for cancer patients, early stage trachoma, stomachache, abdominal cramp, common cold, influenza, urethral infection, all intestinal problems, bone tuberculosis, male sexual impotence, cough, taeniasis, vomiting, diarrhea, mouth odor, & corneal opacity	[55, 60, 63, 64, 68, 69, 77, 78, 87, 92, 98, 101, 106]
